# Lymphotoxin-LIGHT Pathway Regulates the Interferon Signature in Rheumatoid Arthritis

**DOI:** 10.1371/journal.pone.0112545

**Published:** 2014-11-18

**Authors:** Jadwiga Bienkowska, Norm Allaire, Alice Thai, Jaya Goyal, Tatiana Plavina, Ajay Nirula, Megan Weaver, Charlotte Newman, Michelle Petri, Evan Beckman, Jeffrey L. Browning

**Affiliations:** 1 Translational Medicine, Biogen Idec, Cambridge, Massachusetts, United States of America; 2 Immunobiology, Biogen Idec, Cambridge, Massachusetts, United States of America; 3 Global Clinical Operations, Biogen Idec, Cambridge, Massachusetts, United States of America; 4 Johns Hopkins University School of Medicine, Baltimore, Maryland, United States of America; INSERM U1094, University of Limoges School of Medicine, France

## Abstract

A subset of patients with autoimmune diseases including rheumatoid arthritis (RA) and lupus appear to be exposed continually to interferon (IFN) as evidenced by elevated expression of IFN induced genes in blood cells. In lupus, detection of endogenous chromatin complexes by the innate sensing machinery is the suspected driver for the IFN, but the actual mechanisms remain unknown in all of these diseases. We investigated in two randomized clinical trials the effects on RA patients of baminercept, a lymphotoxin-beta receptor-immunoglobulin fusion protein that blocks the lymphotoxin-αβ/LIGHT axis. Administration of baminercept led to a reduced RNA IFN signature in the blood of patients with elevated baseline signatures. Both RA and SLE patients with a high IFN signature were lymphopenic and lymphocyte counts increased following baminercept treatment of RA patients. These data demonstrate a coupling between the lymphotoxin-LIGHT system and IFN production in rheumatoid arthritis. IFN induced retention of lymphocytes within lymphoid tissues is a likely component of the lymphopenia observed in many autoimmune diseases.

ClinicalTrials.gov NCT00664716.

## Introduction

Systemic lupus erythematosus (SLE), rheumatoid arthritis (RA), Sjogren’s syndrome, systemic sclerosis, myositis and multiple sclerosis patients have circulating blood cells with elevated levels of RNA from IFN-induced genes, i.e. an ‘IFN signature’ [1]–[3]. A number of observations point towards a role for IFN in some autoimmune diseases. Notably, risk alleles for SLE include several genes involved in IFN responses. Multiple immunological activities are enhanced by IFN and rodent models of lupus can be accelerated by exogenous IFN. Several rare diseases with lupus-like aspects have mutations in components of the IFN response and are termed ‘interferonopathies’ [4]. Thus, there is very active interest in whether inhibition of IFN signaling has therapeutic benefit [5]. However, the questions of whether the IFN signature is tightly coupled to the pathology in human disease, which immunological detection systems are engaged and what are the actual cellular sources of the IFN, remain unanswered. Moreover, type I (IFN-α, β, ε, τ and ω), type II (IFN-γ) and type III (IFN-λ) IFNs can induce similar patterns of gene expression despite being produced by different spectra of cell types and being under fundamentally different regulation. The varying distribution of receptors for each IFN type also dictates responsive populations and these aspects further confound the problem.

We have investigated the effects of inhibition of the lymphotoxin-LIGHT system in RA using a soluble lymphotoxin-beta receptor (LTBR, TNFRSF3) immunoglobulin fusion protein called baminercept. LTBR is a central component of a signaling system whereby lymphocytes instruct stromal cells to differentiate into specialized vasculature and certain reticular networks [6]–[9]. These components form the gateways for lymphocyte entry into organized lymphoid tissues and the reticular scaffolds that guide and position cells for optimal encounters with antigen. As such, adaptive immune responses within the lymphoid organs are impaired to varying degrees in the absence of LTBR signaling. Additionally, the differentiation of critical sentinel macrophages in the subcapsular sinus of the lymph node (LN) and the splenic marginal zone depend on LTBR signaling [10]. More recently, it has become clear that LTBR signaling is interwoven with aspects of myeloid cell homeostasis as well as more innate elements of the immune system such as communication between dendritic cells, innate lymphoid cells and epithelial surfaces especially in mucosal environments [11]–[15]. Baminercept binds to both LTBR ligands, namely, a membrane bound heterotrimeric lymphotoxin (LT) form LTα1β2 and the ligand called LIGHT (TNFSF14). LIGHT interacts with both LTBR and an additional receptor called HVEM (TNFRSF14) and it has pro-inflammatory roles as well being implicated in aspects of T cell survival [16]. Therefore, baminercept is a dual pathway inhibitor blocking signaling triggered by both membrane LT and LIGHT ligands.

Unexpectedly, we found that baminercept reduced the IFN signature in RA patients. The reduced IFN signature in RA patients following baminercept treatment is the first time outside of high dose steroid therapy that an IFN signature was decreased by a pharmacological treatment not targeting IFN itself. Taken together with the known effects of LTBR inhibition, these studies not only link the LTBR axis to IFN production in man, but also provide potential insight into the nature of the IFN signature.

## Results

### Baminercept reduces the IFN signature in RA patients

**Figure 1:**
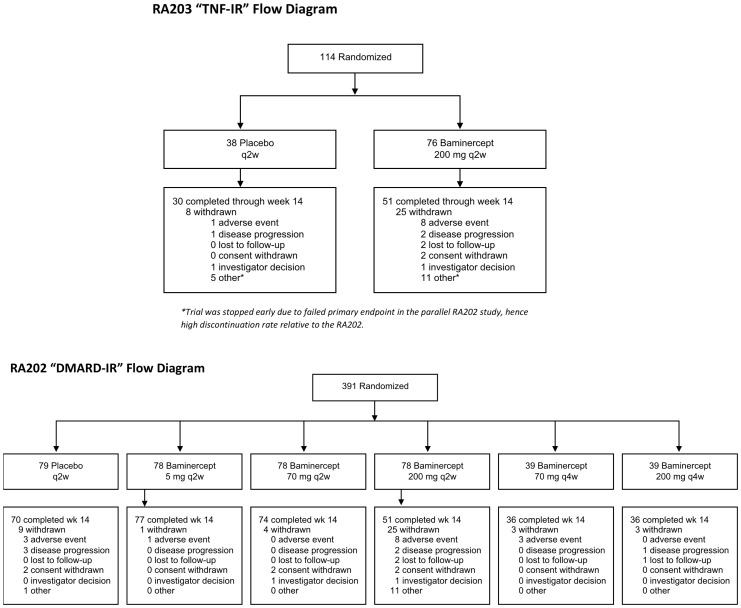
Flow diagrams for the two clinical trials assessing the effects of baminercept treatment on rheumatoid arthritis patients.

Two randomized phase IIb controlled studies of the effects of baminercept in rheumatoid arthritis were conducted. One study enrolled patients with an inadequate response to disease-modifying antirheumatic drug therapy (DMARD-IR) and the other involved patients with an inadequate response to tumor necrosis factor inhibition (TNF-IR) (flow diagrams Figure 1, patient demographics defined in Supplemental Table 1 in File S1). To examine whether baminercept treatment had an impact on the immune system, the transcriptional profiles of whole blood RNA from all RA patients at 0 and 14 weeks were assessed using Affymetrix microarrays. An unsupervised analysis revealed multiple drug-induced changes that fell into three major clusters. First baminercept treatment led to an increased B cell signature. Second, patients with elevated expression at baseline of a collection of IFN response genes had the signature decreased by baminercept treatment and, third, expression of some genes associated with NK cells were decreased following treatment.

**Figure 2:**
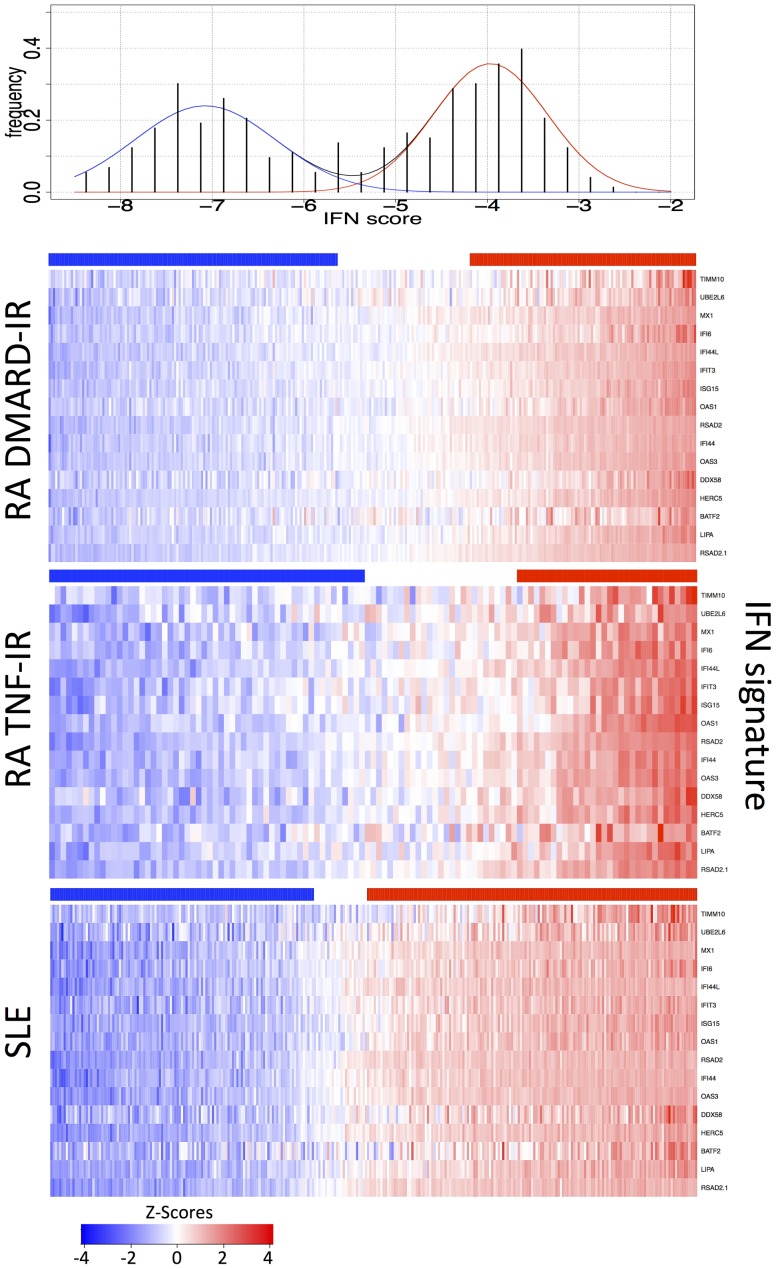
Comparison of the IFN signature in DMARD-IR and TNF-IR RA patients. Baseline heat maps of the RA DMARD-IR, TNF-IR and the SLE cohorts studied in this work. Red indicates increased expression of a panel of 15 IFN inducible genes showing similar percentages of IFN signature positive patients in each RA subgroup (the gene RSAD2 is represented twice). Bars above each map show the clustering as IFN positive (red) or negative (blue) based on assignment to two normal distributions as shown in the top panel with p<0.05. Color bar ranges are as stated for SLE and DMARD-IR, but −3 to 3 for TNF-IR (as per figure 3).

At baseline, roughly 25% of the RA patients in both the DMARD-IR and TNF-IR groups had a high IFN signature (Figure 2) and a 15-gene IFN score was calculated from the genes shown in Figure 2 (Supplemental Table 2 in File S1). Many IFN response gene sets and scores have been utilized in the literature including other sets reported by our group [17]. In general, we found similar results regardless of the selected genes. The IFN signature has been best characterized in SLE and for comparison a control cohort of 292 SLE patients from the Johns Hopkins clinic was analyzed using an identical platform. As expected about 50% of the SLE patients had an elevated IFN signature and the IFN signature in RA patients was slightly weaker than in SLE consistent with a previous study (Supplemental Figure 1 in File S1) [1], [3]. Therefore, in terms of the IFN signature, the RA patients in these studies compare favorably to previous analyses.

**Figure 3:**
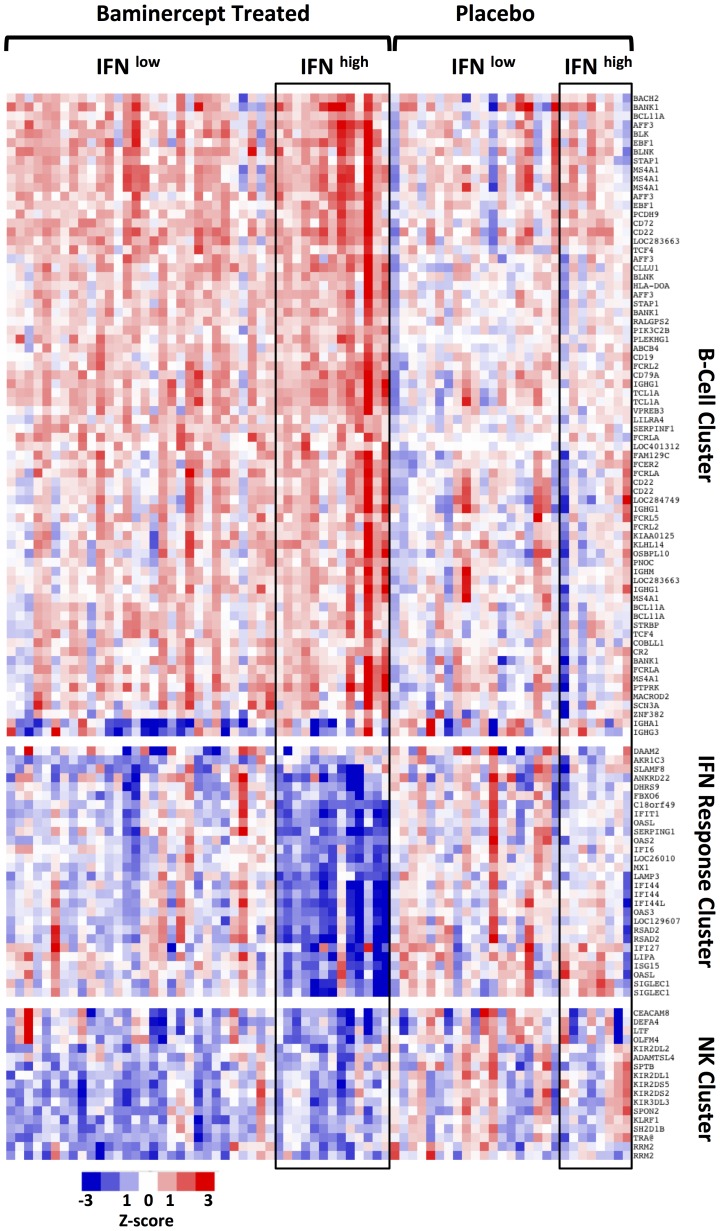
Baminercept induced changes in total blood RNA expression. Heat map showing the change in gene expression after 14 week of either placebo or baminercept treatment. Patients were forced into 4 groups based on treatment and baseline IFN signature. Each of the three gene clusters defined from initial unsupervised clustering are presented separately. The three clusters are characterized by genes associated with B cells, IFN response or NK cells, although some other genes are also present within each category. List only includes genes whose changes were significant (p<0.05), passed FDR and had greater than a 1.5 fold difference in a separate paired sample analysis.

We divided the patients from the TNF-IR study into four groups- placebo and baminercept treated with baseline low or high IFN signature scores. Figure 3 shows a heat map of the expression changes after 14 weeks for all the genes identified in the unsupervised analysis. There was a general increase in a wide range of B cell associated genes, yet interestingly, IgA1 and IgG3 expression decreased. Baminercept can disrupt follicular dendritic cell networks and germinal center reactions and perhaps this decrease reflects impaired class switching and reduced numbers of circulating B cells or plasma cells expressing these immunoglobulins. The second cluster of genes is comprised of genes induced by IFN. Patients with a baseline high IFN score displayed substantial decreases in the IFN score following treatment. The third cluster contains multiple NK related genes and these often decreased following baminercept treatment.

**Figure 4:**
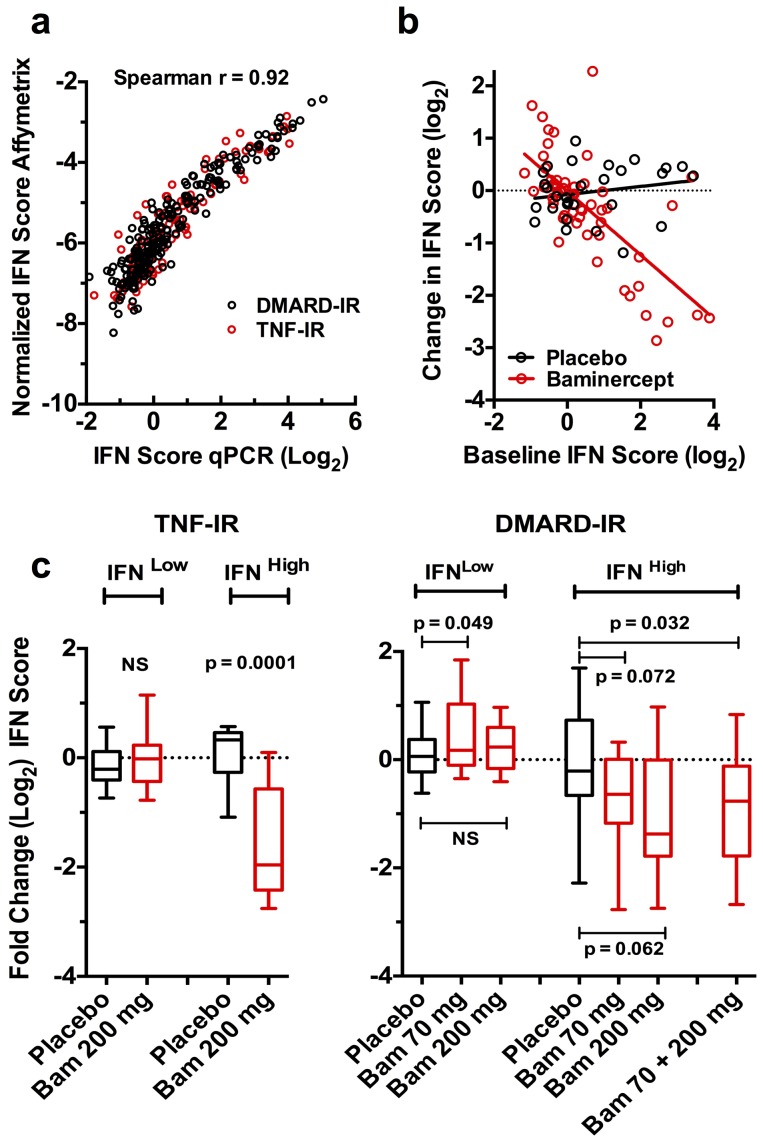
Blockade of the lymphotoxin-LIGHT pathway with baminercept reduces the blood RNA IFN signature in RA patients. a). Analysis of the individual baseline IFN scores as determined using the 15 gene microarray data and a three-gene qPCR score showing excellent correlation. b). Analysis of the change in the 3-gene qPCR IFN score as a function of baseline IFN score following 14 weeks of treatment with 200 mg baminercept q2w in TNF-IR patients, significance is calculated using a linear model of change in IFN score as an interaction of baseline IFN score and treatment (placebo or baminercept). The significance for baseline IFN is p = 2×10^−7^ and for the interaction term p = 2.3×10^−7^. Treatment alone is marginally significant p = 0.0506. c). Change in the qPCR-based IFN score at 14 weeks in patients with low vs. high baseline IFN scores (low <1, high >1). Red boxes represent baminercept (Bam) treated patients receiving either 70 or 200 mg q2w (DMARD-IR) or 200 mg q2w (TNF-IR) while black boxes indicate placebo treated patients; n = 20, 50, 11 and 12 (TNF-IR) and 49, 44, 50, 20, 28, 18 and 38 (DMARD-IR) patients in each category in the order listed. P values are from a Mann-Whitney test of placebo vs. baminercept treated patients.

To further document the IFN signature, the expression of three IFN stimulated genes, Ly6E, ISG15 and OAS1 was determined by quantitative PCR (qPCR) and an IFN score calculated (Supplemental Table 2 in File S1). The qPCR and microarray scores correlated well (Figure 4a). Figure 4b shows the change in the TNF-IR study following 14 weeks of placebo or baminercept treatment in the 3-gene PCR based IFN score as a function of the pre-treatment IFN score. This analysis revealed a significant interaction between the pre-treatment IFN and treatment, interaction p value = 2×10^−7^. A substantial reduction in the IFN signature was also observed at 6 weeks. To extend this observation, the 3-gene qPCR IFN signature was determined for patients in DMARD-IR study. Patients were binned into baseline IFN^high^ and IFN^low^ groups based on the 3-gene qPCR IFN score of greater or less than one. The DMARD-IR study had 6 treatment cohorts and only the 70 and 200 mg q2w cohorts were analyzed by qPCR. Baminercept treatment led to a trend towards a reduced IFN signature in both cohorts and combining the two cohorts showed significant reduction (Figure 4c). The biomarker data indicated approximate saturation of the pharmacodynamic response in both these cohorts justifying combining the data (see below and serum LIGHT measurements Supplemental Figure 2 in File S1). We questioned whether the incidence of infectious events could impact the observation and there was little indication that infection rates were substantially increased or decreased following baminercept treatment (Supplemental Table 3 in File S1). Since baminercept potentially dampens the immune system, an increased rate of infection was possible and therefore treatment could have increased the IFN signature. As an increased IFN signature was not observed, infection is not a likely confounder for this result.

**Figure 5:**
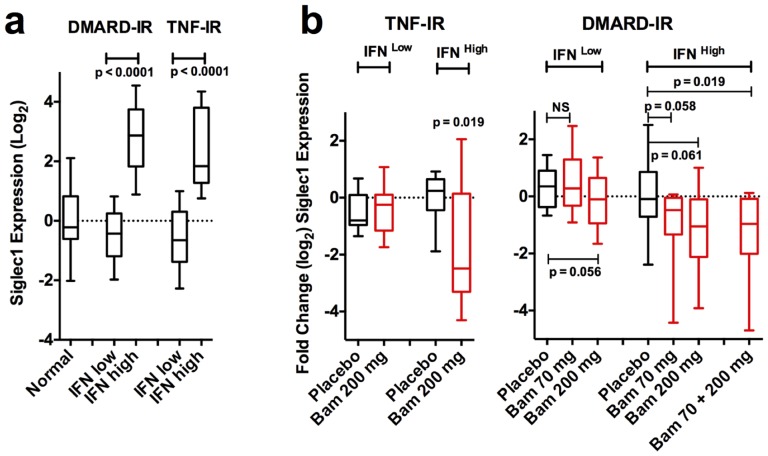
Baminercept treatment lowered RNA expression of the monocyte-associated gene SIGLEC1 in the blood. a). Expression of the monocyte associated gene SIGLEC1 (qPCR determination) is elevated in patients with an elevated IFN signature (qPCR IFN score cut point of 1). b.) SIGLEC1 expression (log_2_) was reduced by treatment with baminercept (n’s and boxes as per figure 4). P values are from a Mann-Whitney test of placebo vs. baminercept treated patients.

To validate further the ability of baminercept treatment to affect an IFN signature, we examined by qPCR the expression of SIGLEC1, another IFN induced gene. In contrast to the genes in the 3-gene panel, it is expressed exclusively in monocytes and, moreover, it is a potential marker of SLE disease severity [18]. Analysis of the SIGLEC1 RNA expression using the qPCR data showed that SIGLEC1 expression was elevated in the IFN high group and baminercept treatment reduced its expression confirming the 3-gene signature analysis (Figure 5a,b). Expression (qPCR) of genes specific for monocytes (SLAMF7, SPARC), DC (CD1E) and plasmacytoid DC (pDC) (CLEC4C and LILRA4) was independent of IFN status.

There is considerable overlap in the expression profiles resulting from type 1 (IFNs α, β and ω) and type II IFN (IFNγ) and, furthermore, each IFN is capable in many contexts of inducing the expression of the other IFN class [19]. An 8 gene IFNγ signature was defined based on genes preferentially induced in blood cells by IFNγ [20]. In our data, there was no significant correlation between the basic IFN and IFNγ signatures suggesting that type I IFNs are dominating in RA (Supplemental Figure 3 in File S1). Two genes, GBP1 and GBP2, were induced selectively by IFNγ in salivary gland epithelial cells, yet in our blood data the GBP1/2 score correlated very well with the basic IFN signature (Supplemental Figure 3 in File S1). In other studies, the GBP1/2 genes are induced by type I IFN in blood cells both in vitro and in vivo in IFN treated hepatitis C and melanoma patients. Our data are consistent with exposure to type I IFN in RA.

Elevated levels of type I IFN or an IFN-like inducing activity can be found in the sera of a subset of the SLE patients with a transcriptional IFN signature; however, in RA the results range from not detectable to low levels relative to SLE sera [21], [22]. We examined serum IFN levels using highly sensitive A549 (more type I IFN selective) or WISH (similar sensitivity to type I and II IFN) cell based reporter assays with an ELISA-based Mx1 protein readout. Analysis of 64 baseline sera from the TNF-IR study including all of the patients with elevated baseline IFN signatures did not reveal IFN activity, whereas substantial activity was readily found in the sera from some SLE patients. Therefore, the blood RNA IFN signature in RA is likely derived from local exposure in organs to IFN.

### IFN signature is associated with lymphopenia in both RA and SLE

**Figure 6:**
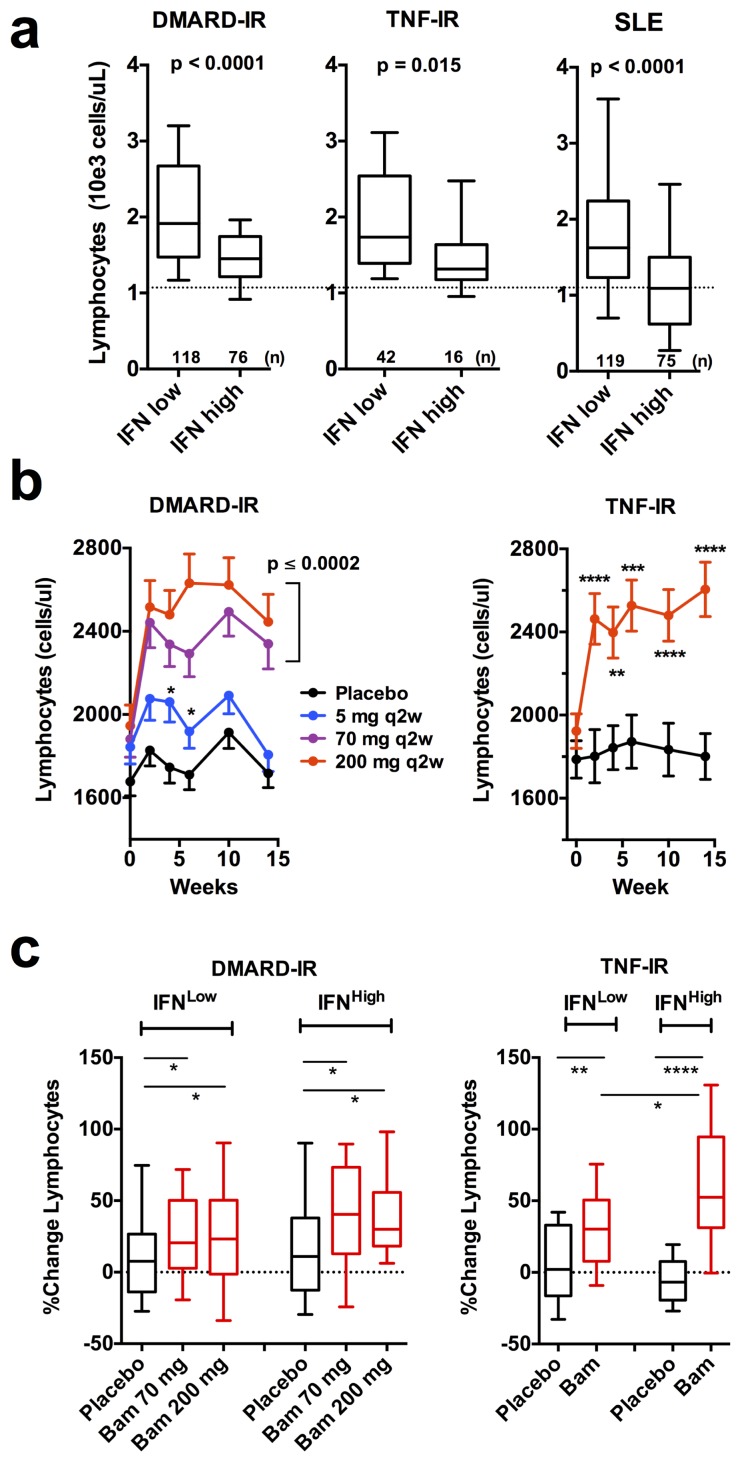
IFN signature positive RA patients are lymphopenic and baminercept treatment resulted in lymphocytosis. a). Patients were segregated based on low and high microarray IFN scores (<−6.5 and>−4.5) and baseline blood lymphocyte counts are plotted. b). Time course of the effects on absolute lymphocyte counts during 14 weeks of baminercept or placebo treatment (means, +/− SEM). All time points in two highest dosed cohorts in DMARD-IR were significant (p<0.0002), otherwise, significance is indicated by p-values * <0.05, ** <0.01, *** <0.001 and **** <0.0001. c). Patients were grouped into baseline qPCR IFN signature low or high as in Fig. 1c. Percent change in lymphocyte counts following 14 weeks of treatment with placebo or baminercept is plotted (significance Mann-Whitney in all cases).

SLE patients with a high IFN signature tend to be lymphopenic [23]. Using the 15 gene microarray-based IFN signature to group patients at baseline into a high or low IFN status, we observed that both IFN high RA and SLE patients were lymphopenic (Figure 6a). The degree of lymphopenia in IFN high RA patients was not as pronounced as in the comparable SLE group possibly paralleling the relative intensities of the IFN signatures in these two diseases.

In rodents, blockade of the LTBR system leads to lymphocytosis within several weeks most likely due to loss of high endothelial venule addressin expression and reduced entry into the lymph nodes and mucosal environments [24]. Treatment with baminercept led to increased lymphocyte and monocyte counts in the blood of patients with full effect observed within 2–5 weeks (Figure 6b and Supplemental Figures 4 and 5 in File S1). The 5 mg q2w dose was partially active and similar results were seen with both the 70 and 200 mg q2w doses indicating approximate saturation. It is believed that one driver for lymphopenia in SLE may be chronic IFN exposure and prolonged lymphocyte retention within the lymph nodes [25]. To assess whether reduced IFN exposure could contribute to the baminercept induced lymphocytosis, we compared the change in lymphocyte counts in the IFN low and high subsets. Lymphocyte counts increased in both groups following baminercept treatment; however, the magnitude of the change was greater in much of the IFN high subset in the TNF-IR study and trended higher in DMARD-IR (Figure 6c). These results are consistent with contributions to baminercept induced lymphocytosis from both altered addressin expression and reduced IFN exposure.

**Figure 7:**
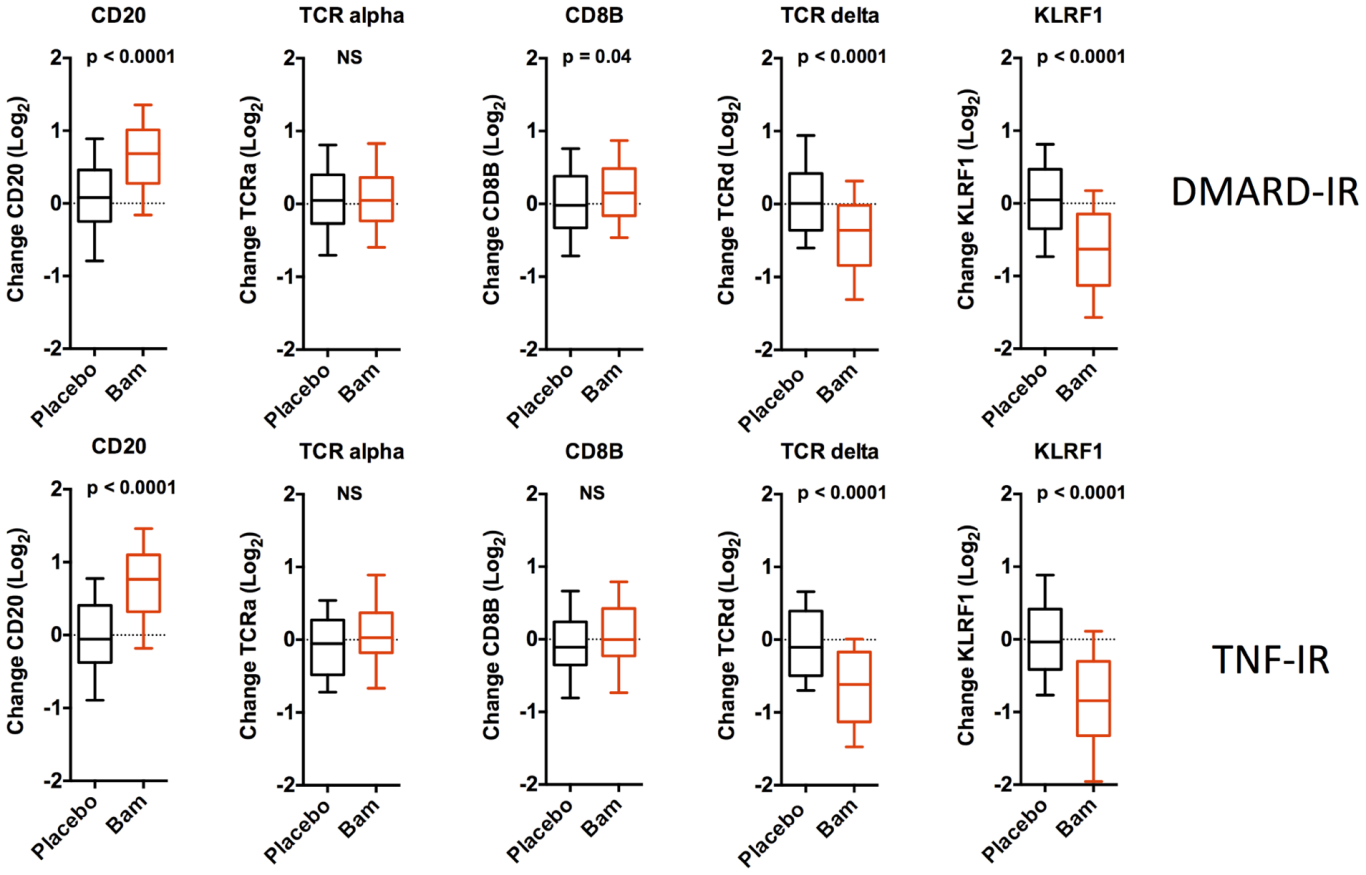
Baminercept alters the levels of RNAs representative of lymphocyte subsets in the blood. Baminercept treatment increased the blood RNA expression (qPCR determination) of genes representing B cells (CD20), T cells (TCRA, CD8B), whereas expression of markers for γδ-T cells (TCRD) and NK cells (KLRF1) decreased. In the DMARD-IR study, data from both 70 and 200 mg 2qw cohorts are pooled.

In mice, lymphocytosis following LTBR blockade results from elevated T and B cells counts [24]. In these clinical studies, FACS analysis of a small subset of the patients (TNF-IR) showed that B cell numbers were increased roughly 70% over baseline compared to a 24% increase in T cells (Supplemental Figure 6 in File S1). The elevated B cell signature based on the data in figure 3 correlated roughly with the extent of lymphocytosis (r = 0.338, p<0.0001,) and therefore it is likely that the B cell signature reflects differential lymphocytosis in the baminercept treated cohorts. Furthermore, PCR quantitation of the levels of genes uniquely expressed in lymphocyte subsets revealed some interesting details (Figure 7). RNA levels of CD20 (B cells) increased consistent with the general lymphocytosis, but TCRδ and some natural killer cell specific genes, KLRF1 as well as KLRD1, KIR2DS1 and KIR3DL1 decreased substantially (only a subset of genes identified from the transcriptional profiling were included in the qPCR array). Thus the qPCR data confirmed the decreased NK signature shown in figure 3. Comparison of the change in absolute lymphocyte counts with the change in RNA levels showed a positive correlation with the expression of CD20, TCRα and CD8B RNA (Spearman r values of 0.56, 0.47 and 0.49 respectively), but little correlation with TCRδ (γδ T cells), KLRF1 (NK) and Defensin 3A (immature neutrophils) (r = 0.14, 0.02 and 0.01 respectively). The change in KLRF1 was not coupled to baseline IFN status. In a FACS analysis, B cells and CD4 cell numbers increased, while NK cell numbers trended towards a decrease (Supplemental Figure 6 in File S1). Therefore baminercept appeared to reduce the numbers of immature neutrophils, TCRγδ and NK cells in the blood by a mechanism distinct from the lymphocytosis effects and prior studies in rodents have implicated LTBR in the biology of both TCRγδ and NK cells [26]–[31]. The decrease in immature neutrophils is intriguing given their propensity to generate chromatin nets and the recent suggestion that nets are a source of citrullinated antigens in RA [32], [33].

Given the emphasis on plasmacytoid DC as the source of the IFN signature in SLE, we examined RNA levels in the blood of two known pDC markers, CLEC4C (BDCA2) and LILRA4 (ILT7). LILRA4 levels increased following baminercept treatment, yet CLEC4C levels were unchanged (Supplemental Figure 7 in File S1). CLEC4C appears to be a relatively specific pDC marker, whereas LILRA4 is highly expressed by both pDCs and memory B cells (Immunological Genome). Therefore these data suggest that baminercept does not affect pDC trafficking and increased LILRA4 expression is consistent with the increased numbers of B cells in the blood.

### Relationship between IFN status and clinical parameters

After 3 months of treatment, changes in disease status were assessed using the American College of Rheumatology (ACR) scoring system. Overall, baminercept was well tolerated (Supplemental Table 3 in File S1); yet, in neither trial did baminercept treatment substantially increase the ACR scores (Supplemental Table 4 in File S1). Baminercept treatment resulted in a trend towards reduced swollen joint counts (SJC28) in both studies (Supplemental Figure 8 in File S1). Inhibition of TNF or IL-6 significantly decreases ESR and serum CRP levels; however, baminercept did not appreciably lower either serum parameter (Supplemental Figure 9 in File S1).

When patients were grouped based on baseline qPCR IFN status, there was no obvious trend towards a greater reduction in SJC28 in either IFN subgroup in the DMARD-IR study (Supplemental Figure 8 in file S1). The TNF-IR study was too small for such subgrouping. As these studies examined almost 800 patients, an analysis of gender effects was possible and baseline IFN scores were slightly lower in RA males (p = 0.04) and trended slightly lower in males in SLE. No correlation was observed between the IFN high and low groups with baseline swollen joint counts (SJC28), the Disease Activity Score 28 ESR (DAS28 ESR), C-reactive protein, erythrocyte sedimentation rates (ESR), rheumatoid factor titer or anti-CCP positivity (Supplemental Figure 10 in File S1). Baminercept treatment did not affect total serum IgG, IgM and IgA levels nor were preexisting tetanus toxoid titers altered.

## Discussion

We show here that inhibition of the LT/LIGHT pathway reduced not only the global IFN signature, but also RNA levels of SIGLEC1, an IFN-regulated gene expressed only in monocytes. RA and SLE patients with an IFN signature were more lymphopenic and baminercept treatment reversed the lymphopenia. These results demonstrate at the clinical level a fundamental linkage between the LT/LIGHT axis and IFN responses as well as between IFN and lymphopenia. To date, only high dose steroids and anti-interferon-α antibodies have been shown to reduce the signature and the inhibition by anti-IFNα antibodies appears to be partial [34]–[39]. Neither therapy informs on the nature of the underlying IFN biology. Importantly, a reduction in the IFN signature did not result in diminished arthritis suggesting that the pathology driving the IFN signature is not tightly coupled to the local joint disease. This conclusion needs to be qualified because the trials were insufficiently powered for the IFN positive subset, the treatment duration was relatively short and the levels of inflammatory disease were modest as indicated by the low baseline CRP levels.

The question of what biology is being reflected by the blood IFN signature in RA as well as in other autoimmune diseases remains unanswered. In SLE, the IFN signature is correlated in general with a distinct serological profile, renal disease, and progression to atherosclerosis and disease severity, yet its presence is not obviously linked to any particular immunology [23], [40]. Our RA studies and those of others did not reveal obvious differences based on IFN status between the DMARD-IR and TNF-IR cohorts or their clinical or serological features [41], [42]. A positive IFN signature in RA has been linked to a poor response to rituximab [43] and a weak or variable correlation with response to TNF inhibition [22], [44], [45]. In both systemic onset juvenile idiopathic arthritis patients and in Sjogren’s syndrome, TNF inhibition led to an increased IFN signature supporting the hypothesis that TNF- and IFN-driven pathologies may lie at opposite poles of the autoimmune disease spectrum [46], [47]. The ability of baminercept to dampen an IFN signature may have been a liability in a TNF dominated setting such as RA.

SLE patients are often lymphopenic and lymphopenia correlated with an elevated IFN signature in both SLE and systemic sclerosis patients [23], [48]. Here, we confirmed this association in our SLE cohort and extended it to include RA patients. Lymphopenia could reflect perturbed bone marrow hematopoiesis accompanying systemic inflammation and/or increased retention of lymphocytes in the lymph nodes [49], [50]. In the latter mechanism, IFN triggers complex formation between CD69 and sphingosine-1-phosphate receptors thereby inhibiting cell egress into the cortical sinuses [25]. Indeed, administration of IFNβ to multiple sclerosis patients or mice results in lymphopenia [51], [52]. Increased retention of lymphocytes in the IFN-rich lymphoid microenvironments would allow more dwell time for productive encounters. Baminercept-induced lymphocytosis most likely has a contribution from restricted entry into the LN and mucosal compartments due to loss of addressin positive high endothelial venules. Baminercept could also reduce lymphopenia by shortening retention times in the LN following reduced IFN exposure. Our data are consistent with contributions from both trafficking and retention components and support the hypothesis that IFN-driven lymphocyte retention in the lymphoid tissues is a substantial component of lymphopenia in autoimmune disease.

The effect of baminercept on the blood IFN signature was largely unanticipated; however, observations from several experimental systems demonstrate that the LT/LIGHT network is entwined with IFN responses to infection [53]. First, murine CMV infection of the spleen induces an early IFN response derived from reticular stromal cells in the marginal zone [31], [54], [55]. Various aspects of lymphoid reticular stroma are critically dependent upon LTBR signaling and loss of LTBR signaling ablated the initial IFN response to murine CMV infection. Second, SIGLEC1-positive sentinel macrophages in both the LN and splenic environments survey lymph or blood for immune complexes, lipids, particulates, viruses and sialylated antigens [56]–[58]. In the case of immune complexes or complement tagged antigens, they capture these elements and route them into the follicle for presentation to B cells. The differentiation state of these sentinel macrophages is LTBR-dependent [59], [60]. Following VSV infection, virus replicates within the LN subcapsular macrophages and the splenic marginal zone metallophilic macrophages triggering IFN production and an effective host response [60]–[62]. Within the spleen, marginal zone metallophilic macrophages are the major local producers of type I IFN in response to an intravenous HSV challenge [63] and they also generate IFN in response to *Campylobacter jejuni* infection [64]. In a related observation, listeria infection of LTBR-deficient mice failed to generate an IFN response in the spleen [65]. Other LT-dependent sources could include both dendritic cells [11], [66]–[68] as well as follicular dendritic cells where TLRs could be activated following internalization and recycling of antigen [69].

Why is this linkage between the LT and IFN systems manifesting itself in RA? In SLE, a subset of IFN signature high patients has measureable IFN circulating in the blood [21], [70]. However, IFN was not detected in the blood of our RA patients and therefore the exposure to IFN must occur while leucocytes traffic through the organs. In RA, robust evidence for a substantial IFN signal in the joints is lacking [3], [71], [72]. One hypothesis is that lymphocytes become “imprinted” by IFN while trafficking through organized lymphoid microenvironments. Many if not all autoimmune diseases have undercurrents of systemic disease as evidenced by the involvement of additional organ systems, e.g. the lungs in RA, the CNS in SLE, Sjogren’s and sarcoidosis, etc. We speculate that LNs draining organs with articular or extra-articular disease, as in lung or glandular involvement in RA, produce IFN as a consequence of sensing signals such as dead cell debris, chromatin complexes, neutrophil nets or antigens as complement tagged or immunoglobulin complexes [69], [73]. In this scenario, the amount of IFN exposure would reflect the magnitude of the systemic involvement and may coincide with LN reactivity or even gross lymphadenopathy. Indeed, some association was noted in SLE between lymphoadenopathy and an increased IFN signature [23]. We speculate that baminercept’s effects on the IFN signature are due to alteration of the sentinel functions of the lymphoid microenvironments albeit via myeloid or stromal elements.

In conclusion, inhibition of the LTR signaling in RA patients reduced IFN imprinting. The IFN signature is linked to the lymphopenia in RA and SLE supporting a role for IFN in lymphocyte retention in lymphoid organs. Experimentally, the use of viral challenges has revealed much of the linkage between LTBR and IFN responses and, while autoimmune diseases do not have obvious ongoing viral infections, parallels have been drawn between immune responses to virus and chromatin in SLE [74]. Thus, these observations may be highlighting a potential coupling mechanism between tissue damage, debris recognition and an overactive self-reactive immune response. Disruption of LTBR signaling could be a new tool for the investigation and potentially the treatment of certain subgroups in autoimmune diseases.

## Experimental Procedures

### Patients and Trials

Baminercept is a fusion protein of the extracellular domain of human LTBR coupled to the hinge and Fc domain of human IgG1 [75]. DMARD-IR (104RA202, NCT00664716, EUdraCT 2006-005466-39) was a multicenter, phase IIb, randomized, double-blinded placebo-controlled study of RA patients who had had an inadequate response (IR) to a disease modifying anti-rheumatic drug. In this study 391 RA patients were treated for 14 weeks with subcutaneous injections of placebo q2w (79 patients), 5 mg baminercept (BG9948) q2w (78), 70 mg q2w (78), 70 mg q4w (39), 200 mg q2w (78) or 200 mg q4w (39). 365 patients completed the study. Study was conducted between July 2007 and October 2008 at 58 sites in Argentina, Brazil, Hungary, Mexico, Poland, Romania, Russia and United Kingdom and investigators are listed in the supplemental materials.

TNF-IR (104RA203, NCT00458861, EUdraCT 2006-005467-26) was a multicenter, phase IIb, double-blinded, placebo-controlled study of RA patients who lacked an adequate response to TNF-blocking therapy and had discontinued TNF blocking treatment for at least 90 days. The study dosed 114 patients with subcutaneous injections q2w of either placebo (38) or baminercept 200 mg (76). This study was terminated early due to poor efficacy in the DMARD-IR study; however, 81 patients completed the 3 months of dosing and another 15 patients received at least 2 months of treatment. Study was conducted between March 2007 and October 2008 at 40 sites in the United States, Canada, Belgium and United Kingdom.

Investigators for both baminercept studies are listed in the supplemental materials in File S1. RA patients in both studies were eligible if they met the American College of Rheumatology (ACR) criteria for rheumatoid arthritis and had active RA for at least 6 months. Patients had to have been receiving 10–25 mg methotrexate per week for at least 3 months with a stable dose for the last 4 weeks before entry. Methotrexate therapy was maintained for the duration of the studies. Patients had to have more than 8 swollen and tender joints (66/68 joint count) and either a CRP≥1.5 ULN or ESR ≥28 mm/hr at screening. The protocols for these trials re available as supporting information; see Protocol S1 and S2. There is no intent to publish further clinical data from these studies and the ACR scores (primary trial endpoint) are presented within the supplemental data.

SLE data were from registry (called SPARE) representing a collection of 292 SLE patients from the Hopkins Lupus Center in the US. Patients were eligible if they were aged 18–75 years and met the American College of Rheumatology Revised Criteria for Classification of Systemic Lupus Erythematous. Baseline data were used in this study and patients were under standard clinical practice. Normal controls for RNA analyses were composed of healthy volunteer donors from Biogen Idec. Control group had equal numbers of males and females and was not exactly gender balanced with the predominantly female composition of both the RA and SLE cohorts.

### Ethics Statement

The RA studies were approved by the appropriate institutional review boards or ethics committees and all patients provided written informed consent (see supplementary materials in File S1 for a complete listing). All patients from the Hopkins Lupus Center (Johns Hopkins University School of Medicine) provided written informed consent to participate in the SPARE registry and the Johns Hopkins institutional review board approved the study.

### Analyses of whole blood RNA

Patient whole blood was collected into PaxGene tubes and analyzed using conventional Affymetrix microarrays and qPCR was performed on the RNA samples using the Fluidigm analyzer. Details for RNA analyses as well as the IFN reporter assays are provided in the Supplemental Materials in File S1. Baseline transcriptional profiling datasets are deposited at GEO, GSE45291.

## Supporting Information

File S1 **Supporting files.** Supplemental Methods and Investigator Listing. Supplemental Table 1, Demographics of the Patients in the RA studies and the SLE registry. Supplemental Table 2, Measurement of IFN signatures. Supplemental Table 3, Incidence of adverse events by preferred term for the combined placebo-controlled studies. Supplemental Table 4, Lack of an appreciable effect of baminercept treatment on ACR scores as assessed at 14 weeks. Supplemental Figure 1, Comparison of IFN signatures in RA and SLE. Supplemental Figure 2, Elevation of serum LIGHT levels in RA patients following baminercept treatment. Supplemental Figure 3, Characteristics of the IFN signatures observed in this study. Elevation of serum LIGHT levels in RA patients following baminercept treatment. Supplemental Figure 4, Characteristics of baminercept induced lymphocytosis in RA patients. Supplemental Figure 5, Relationship between IFN signature and baminercept treatment on blood neutrophil and monocyte counts. Supplemental Figure 6, FACS analysis of the effects of baminercept treatment on peripheral blood lymphocyte subsets. Supplemental Figure 7, Quantitative PCR analysis on whole blood RNA of the effects of baminercept treatment on gene markers of myeloid subsets. Supplemental Figure 8, Effects of baminercept treatment on the Swollen Joint Count 28 (SJC28) scores in the DMARD-IR and TNF-IR studies. Supplemental Figure 9, Little effect of baminercept treatment on CRP levels and Erythrocyte Sedimentation Rates (ESR) in the DMARD-IR and TNF-IR studies. Supplemental Figure 10, The IFN signature status in RA patients does not correlate with clinical or serological parameters.
(PDF)

Checklist S1 **CONSORT Checklist.**
(PDF)

Protocol S1 **Trial Protocol.**
(PDF)

Protocol S2 **Trial Protocol.**
(PDF)
