# Supplementary material for: Lymphotoxin-LIGHT Pathway Regulates the Interferon Signature in Rheumatoid Arthritis
Source: PLoS One. 2014 Nov 18;9(11):e112545. doi: 10.1371/journal.pone.0112545 (PMC4236099; doi:10.1371/journal.pone.0112545)

Supplemental Methods:

Analyses of whole blood RNA:

Whole blood was collected from all patients into PaxGene tubes at pre-treatment (week 0), after 6 and 14 weeks of treatment in TNF-IR and at 0 and 14 weeks in DMARD-IR. RNA was extracted, processed and hybridized to Affymetrix microarrays using the globin-signal reduction protocol as described (1,2). The RNA was profiled using the same high-throughput Affymetrix chip HTHGU133plusPM platform. RNA profiles from each of the two trials were normalized and QC'ed separately using the GCRMA method implemented in BioConductor. This cross-trial normalization was only employed for the comparison of IFN signatures between RA and SLE and the correlation between IFN status and lymphopenia. Samples with high variation in normalized un-scaled standard error and relative log error were removed. We also excluded from further analysis samples with high RNA degradation rates (>4). Transcriptional profiles from a total of 759 samples from 389 patients (DMARD-IR) and 325 samples from 110 patients (TNF-IR) passed control. Gene expression signatures for IFN and Type II IFN were created based on the genes listed in supplementary figure 1. Scores were the geometric means of the microarray signals after normalization. To compare expression values across the RA and SLE studies, the individual values for β–actin expression were subtracted from the normalized gene score. Baseline transcriptional profiling datasets are deposited at GEO, GSE45291.

RNAs from all TNF-IR patients and the 0, 70 and 200 mg q2w cohorts from DMARD-IR were analyzed at weeks 0 and 14 by qPCR using the Fluidigm® analyzer. SLE patient samples were not analyzed by qPCR. A total of 659 RNA samples passed quality control including 17 controls, 436 DMARD-IR (207 data pairs week 0-14) and 207 TNF-IR (94 data pairs weeks 0-14) . Primer sets for the Fluidigm® analyses are listed below. Cycle thresholds were averaged from quadruplicate analyses and normalized to UBC and YWHAZ. The values were then normalized to the mean of the values from 17 control subjects. The 3-gene qPCR IFN score was the average of ISG15, Ly6E and OAS1 values and the range of each of the individual values was roughly similar indicating approximately equal weighting.

IFN Reporter Assay

An assay similar to one previously published was employed (3). Confluent layers of A549 or WISH cells were cultured in 96 well plates in test serum at a 1:5 dilution for 24 hours. The cells were lysed in a detergent solution and the lysate was assayed for Mx1 content using a conventional ELISA with a pair of Mx1 specific antibodies (developed at Biogen Idec). ED_{50} for IFNα4, IFNβ and IFNγ was 1, 15 and 400 IU/ml respectively using the A549 cells and 5, 9 and 9 IU/ml with WISH cells. The A549 based assay had a lower limit of quantitation of about 0.3-1.0 IU/ml or about 1.5-5 pg/ml and has been validated for detection of IFNβ (Avonex®) in human serum. This level of sensitivity is similar to assays based on MHC class I expression or better than typical antiviral or anti-proliferative readouts (3). Reference human α and ω interferons were obtained from PBL Interferon Source and IFN-β was from Biogen Idec.

Statistics

Analyses were performed using Prism software (GraphPad). Unless specified otherwise, two-tailed Mann-Whitney tests were used to determine significance and either p values are noted or indicated as * $p < 0.05$, ** $p < 0.01$, *** $p < 0.001$, **** $p < 0.0001$. Correlations were assessed with the Spearmen rank analysis. In the DMARD-IR trial, there were 391 patients with 365 (93%) completing. The TNF-IR trial was terminated early due to the lack of efficacy in DMARD-IR. In that study, 114 patients were dosed of which 81 completed. An additional 15 patients completed at least 2 months weeks of dosing and, in those cases, the last observation was carried forward for all lymphocytosis, qPCR, CRP, ESR and SJC analyses. In the DMARD-IR study, qPCR data from one patient each from the placebo and 70 mg q2w groups was excluded as the baseline IFN signature score was greater than +5. These patients were also identified by a ROUT outlier analysis for non-parametric data (Q = 0.1%) and these were the only two RA patients with such high baseline scores in both trials.

Supplemental Methods Continued:

Primer sets for quantitative PCR used in this paper. Five probe sets had some failed reactions: CLIC3 (94% successful), KIR2DL1 (74%), KIR2DS1 (99%), LILRA4 (89%) and UBD (92%), n =301 patients.

Gene	Common Name	Target	Forward	Reverse	Probe
Cd1e	CD1E	DC	GGAAGACACGCCTAAACCAACT	GTTTAAGAAGATGCAGACAAGGATTTT	6FAM-TGGTGACATTTGCTTTAC
Cd8b	CD8 beta	CD8 T Cells	CGCAGGACAGCATCACCAT	TCCAGCAGACCCCTGCATAC	6FAM-TGTTGGTTTGCACCTTT
Clec4c	BDCA2	pDC	AAAGAACCACACCCCGAAAGT	GGTGGGTGCAGAAGCTCTTG	6FAM-ACATCTTTGGAGAAAGTGAT
Clic3	CLIC3	NK	CCAGCGTCTCCTCCAGAAAG	CGTTTTCCACGCCAAGACA	6FAM-CCTCGATCTGCAGCGTG
Defa3	Defensin A3	Imm Neutrophil	AGCCCCGGAGCAGATTG	TGCAAGGGAAACAACCACTTC	6FAM-AGCGGACATCCC
G1p2	ISG15	IFN ISG	TGGCGGGCAACGAATT	GGGTGATCTGCGCCTTCA	6FAM-TGAGCAGCTCCATGTC
Gapdh	GAPDH	Housekeeping	ACCACCAGCCCCAGCAA	GGGACTCCCCAGCAGTGA	6FAM-AGCACAAGAGGAAGAGA
Ifi27	IFI27	IFN ISG	CAGTGCCATGGGCTTCACT	ACATCATCTTGGCTGCTATGGA	6FAM-CGGGAATCGCCTCGT
Itgae	Integrin alphaE	Integrin alphaE	AACAGCTCCATGCTGCTCTAGAT	GACCCAGCATCCTTTGCATT	6FAM-ATCCTGAAGGAAAAAG
Itgb7	Integrin Beta 7	Integrin Beta 7	CAGCATGGACAGGTGAGGATT	AGAGTGAATTGGACGCCAAGA	6FAM-CCATTCTGTGGCATCC
Kir2dl1	KIR2DL1	NK	GTTTTCCCTCCTTCAAATAAACATG	TTAGGCAAGAAAAGAGTCCCATTAC	6FAM-CTGCCCTCATGGTTT
Kir2ds1	KIR2DS1	NK	TCCCGGAGCTCCTATGACAT	GCAGGGAGCCTACGTTCATG	6FAM-TACCATCTATCCAGGGAAG
Kir3dl1	KIR3DL1	NK	AAAGTTGTCTCCTGCCCATGA	GGGCTGTTGTCTCCCTAGAAGA	6FAM-CACCACAGTCAGGCC
Klrd1	KLRD1	NK	CCGGTGCAACTGTTACTTCATTT	GATGCCGACTTTCGTTCCA	6FAM-CAGTGAACAGAAAAC
Klrf1	KLRF1	NK	AAAGGGACCAGCTAAAGAAAACAG	TGCTGCAGGTTTCAGAGAAAATT	6FAM-TGTGCTGCCATTAAG
Lilra4	ILT7	pDC	CCCCCTCCTTGGTCAGAAG	AAGGTGACCCTGCTGTGTCAGT	6FAM-AAAGTGAACATCGGGTCC
Ly6e	LY6E	Monocytes, IFN ISG	TCCCAGAAGGCGTCAATGTT	CGCACTGAAATTGCACAGAAA	6FAM-TGGCTTCCATGGGC
Ms4a1	CD20	B Cells	ATGAAAGGCCCTATTGCTATGC	CAGTGAAGACATCCTCCTGAAGAG	6FAM-TCTGGTCCAAAACC
Oas1	OAS1	IFN ISG	CACAGCCCAGGGATTTCG	TTGTCCAGTAGATGCAGAGTTGCT	6FAM-CTTGGAATTAGTCATAAACTAC
Rsad2	RSAD2	IFN ISG	GTTGACATGGAGGCAGTGCTT	CAGCCTCATGTGGCCAGAT	6FAM-CATTGCTTTGTTCGCCTAT
Siglec1	SIGLEC1	Monocytes IFN ISG	CAAGGGAGACTGGGAAATGTAGTTT	ATTCCCAACAATGTCAAAAGTCTCA	6FAM-AGGACATTTGGAATTGGA
Slamf7	SLAMF7	Monocytes, Act Mac, DC	TGTGCATGGCCCAAGGA	TGCAGCACAAGTGCATAAATGA	6FAM-AGGACCTCCAGCCAGG
Sparc	SPARC	Monocytes	ACCGATTCACCAACTCCACTTT	CAGCCAGGAAGGCCAAAA	6FAM-CTTTCTACATCTCACTCTTG
Tnfrsf10c	TRAIL-R3	Neutrophils	AAAGTTCGTCGTCGTCATCGT	GCAGTGGTGGCAGAGTAAGCT	6FAM-CGGTCCTGCTGCCAG
Tra@	TCR alpha	αβ T Cells	CTGACCCTGCCGTGTACCA	TCGGTGAATAGGCAGACAGACTT	6FAM-CTGAGAGACTCTAAATCCAGT
Trad	TCR delta	γδ T Cells	CCGAGAAGGTGAACATGATGTC	CAGTCTTTGCAAACAGCATTCG	6FAM-TCACAGTGCTTGGGC
Ubc	Ubiquitin C	Housekeeping	CACTTGGTCCTGCGCTTGA	AGTGCAATGAAATTTGTTGAAACC	6FAM-TGTCTAAGTTTCCCCTTTTA
Ubd	Ubiquitin D	Inflammation	TGCCGTAATCTGCCATCATC	CCAGATTGTGACTTGCAATGGA	6FAM-TCCCATCTTCCAGTCTC
Ywhaz	Monooxygenase	Housekeeping	TGGAAAAAGGCCGCATGAT	TGGGATGCAAGCAAAGGAA	6FAM-TTTCTGGCTCCACTCAG

References Supplemental Methods

1). Allaire NE, Rieder LE, Bienkowska J, Carulli JP. (2008) Experimental comparison and cross-validation of Affymetrix HT plate and cartridge array gene expression platforms. *Genomics* 92:359-65

2). Allaire, NE., Bienkowska, J., Brock, G., Carulli, J. (2013) Optimization of a high-throughput whole blood expression profiling methodology and its application to assess the pharmacodynamics of interferon (IFN) beta-1a or polyethylene glycol-conjugated IFN beta-1a in healthy clinical trial subjects. *BMC Res Notes* 6, 8

3). Vallittu A-M, Eralinna J-P, Ilonen J, Salmi AA and Waris M. (2007) MxA protein assay for optimal monitoring of IFN-beta bioactivity in the treatment of MS patients. *Acta Neurol Scand* 118, 1-17

4). Da Silva, A. J., M. Brickelmaier, G. R. Majeau, A. V. Lukashin, J. Peyman, A. Whitty, and P. S. Hochman. (2002) Comparison of gene expression patterns induced by treatment of human umbilical vein endothelial cells with IFN-alpha 2b vs. IFN-beta 1a: understanding the functional relationship between distinct type I interferons that act through a common receptor. *J Interferon Cytokine Res* 22: 173-188

Supplemental Methods Continued:

RA202 Investigators

Study Number	Country	Investigator	Institution and IRB	Site Address	City	State
104RA202	ARGENTINA	Eimon, Alicia	CEMIC	Galvan 4102	Buenos Aires	
104RA202	ARGENTINA	Venarotti, Horacio	Atencion Integral en Reumatologia	Av Cabildo 1131	Buenos Aires	
104RA202	ARGENTINA	Martins, Silvia	Hospital Espanol	Belgrano 2975	Buenos Aires	
104RA202	ARGENTINA	Spindler, Alberto	Centro Medico Privado de Reumatologia	Lavalle 506	Tucuman	
104RA202	ARGENTINA	Tate, Guillermo	Organizacion Medica de Investigacion (OMI)	Uruguay 725 PB	Buenos Aires	
104RA202	ARGENTINA	Velasco, Jorge	CER Instituto Medico	Vicente Lopez 1441	Quilmes	
104RA202	ARGENTINA	Lazaro, Alicia	CIDEA	Paraquay 2035 2 Sub 3 Cuerpo	Buenos Aires	
104RA202	ARGENTINA	Alvarellos, Alejandro	Hospital Privado S.A.	Av. Naciones Unidas 346 3piso	Cordoba	
104RA202	ARGENTINA	Zoruba, Diana	Grupo Medico Lomes de San Isidro	Monsenor Magilamo 3041	San Isidro	
104RA202	BRAZIL	Silva, Marilia	HEC -Hospital Evangelico de Curitiba	Alameda Augusto Stelfield 2034	Curitiba	
104RA202	BRAZIL	Zerbini, Cristiano	Hospital Heliopolis	Unidade de Gestao Assistenical Av Almirante de la Mare	Sao Paulo	
104RA202	BRAZIL	Azevedo, Mario Newton	Universidade Federal do Rio de Janeiro - Hospital Universita	Departamento de Reumatologia Av. Rodolfo Rocco,	Rio de Janeiro	
104RA202	BRAZIL	Keiserman, Mauro	Hospital Sao Lucas da PUCRS	Uniao Brasilleira de Educacao Av. Ipiranga 6690	Porto Alegre	
104RA202	BRAZIL	Brenol, Joao Carlos	Hospital de Clínicas de Porto Alegre	Rua Ramiro Barcelos, 2350	Porto Alegre	
104RA202	BRAZIL	Levy, Roger	Centro de Ensino e Pesquisas do Pro Cardiaco - PROCEP	Departamento de Pesquisa Rua General Polidorio, 192	Rio de Janeiro	
104RA202	BRAZIL	Lage, Lais Verderame	Fundacilao Faculade de Medicina	Avenida Doutar Amaido n455	Sao Paulo	
104RA202	BRAZIL	Radominski, Sebastiao	Hospital de Clinicas da Universidade Federal do Parana	Cenro de Estudos em Terapias I Cenro de Esturdos em Terapias Inovadoras- CETI	Curitiba	
104RA202	BRAZIL	Pollak, Daniel Feldman	Instituto Paulista de Reumatologica	Rua Dr. Bacelar, 208	Sao Paulo	
104RA202	BRAZIL	Scheimberg, Morton	Hospital Abreu Sodre	Associacao de Assistencia a Cr Avenida Professor Ascendino Reis	Sao Paulo	
104RA202	BRAZIL	Ximenes, Antonio Carlos	Hospital Geral de Goiania	Pesquisa Clinica/Reumatolo Av. Anhanguera	Goiania	
104RA202	HUNGARY	Szekanecz, Zoltan	University of Debrecen	3rd Clinic - Internal Medicine Moricz Zs. krt 22	Debrecen	
104RA202	HUNGARY	Geher, Pal	Polyclinic of the Hospitaller Brothers of St John of God	2nd Department of Rheumatology Frankel Leo ut 17-19	Budapest	
104RA202	HUNGARY	Ferincz, Vera	Zala Megyei Korhaz	Department Of Neurology Zrinyi u. 1,	Zalaegerszeg	
104RA202	HUNGARY	Szombati, Istvan	Diagnostic Units Hungary	SMO Becsi ut 61	Budapest	
104RA202	HUNGARY	Gyrogy Kiss, Csaba	Pecsi Tudomanyegyetem	Department of Rheumatology Rheumatologiai es Immunologiai	Pecs	
104RA202	HUNGARY	Keszthelyi, Peter	Pandy Kalman County Hospital	Rheumatology Semmelweis u. 1	Gyula	
104RA202	HUNGARY	Toth, Edit	Flor Ferenc County Hospital	Reumatology Semmelweis ter 1.	Kistarcsa	
104RA202	HUNGARY	Rojkovich, Bernadette	Budai Irgalmasrendi Korhaz	I szamu Reumatologiai Osztaly Frankei Leo ut 33	Budapest	
104RA202	HUNGARY	Ujfalussy, Ilona	Allami Egeszsegugyi Kozpont	Reumatologiai Osztaly Podmaniczky u. 109-111	Budapest	
104RA202	MEXICO	Sanchez, Jorge Guerrero	Instituto Nacional de Ciencias	Medicas Y Nutricion "Dr Salvad Vasco de Quiroga 15	Mexico City	
104RA202	MEXICO	Xibille, Daniel Xavier Friedmann	Hospital Inovamed	Administradora de Clinicas, SA Calle Cuauhtemoc #203-109	Cuernava	
104RA202	MEXICO	Cons, Francisco Fidencio Molina	OSTEO MED, SC	Clinica de Artritis y Osteopor Alvaro Obregon 1257-1	Mexicalli	
104RA202	MEXICO	Villarreal, Arturo Anselmo Rios	Hospital y Clinica OCA	S.A. de C.V. Pablo A. Gonzalez 709	Monterrey	
104RA202	MEXICO	Abud, Carlos Mendoza	Hospital Central "Dr. Ignacio Morones Prieto"	Departmento de Reumatologia Av. Venustiano Carranza 2395	San Luis Potosi	
104RA202	POLAND	Filipowicz-Sosnowska, Anna	Instytut Reumatologiczny	Klinika Reumatologi ul. Spartanska 1	Warsaw	
104RA202	POLAND	Jeka, Slawomir	Szpital Uniwersytecki nr 2 im.dr J. Biziela	Praktyka Lekarza Rodzinnego Na ul. Szcztyna 20	Bydgoszczy	
104RA202	POLAND	Dudek, Anna	Centrum Leczenia Chorob Cywilizacjnych	Al. Komisji	Warsaw	
104RA202	POLAND	Szczepanski, Leszek	Osrodek Badan Klinicznych	ul. Krucza 5	Lublin	
104RA202	POLAND	Leszczynski, Piotr	Indywidualna Specjalistyczna Praktyka Lekarska	Piotr Leszczynski ul. Hetmanska 55/1	Poznan	
104RA202	POLAND	Chudzik, Dariusz	Niepubliczny Zakland Opieki Zdrowotnej (NZOZ) Reumed	Department of Rheumatology ul. Konrada Wallenroda 2F/4	Lublin	
104RA202	POLAND	Pokrzywnicka-Gajek, Ines	Centrum Medyczne Osteomed sp. z o.o.	ul. Al. Krakowska 110/114	Warsaw	
104RA202	POLAND	Jeka, Slawomir	Oddzial Reumatologii z Pododdzialem Rehabilitacji	Szpital Wojewodzki im. Dr J. B ul. Ujejskiego 75	Bydgoszcz	
104RA202	ROMANIA	Udrea, Gabriela	Dr. I. Cantancuzino Hospital	Clinic of Intrenal Medicine an 5-7 Ion Movila Street	Bucharest	
104RA202	ROMANIA	Cristei, Dorcia	Promedica Policlinic	Rheumatolgy Clinic 2 Independentei Street	Braila	
104RA202	ROMANIA	Bolosiu, Horatiu	Emergency Clincial County Hospital Cluj	Rheumatology Clinic 2-4 Clinicilor Street	Cuji Napoca	
104RA202	ROMANIA	Pavel, Mariana	Emergency Hospital "St Apostol Andrei" Galati	177 Brailei Street	Galati	
104RA202	ROMANIA	Rezus, Elena	Investigati Medicale Praxis	33 Independentei Blvd	Iasi	
104RA202	ROMANIA	Codreanu, Catalin	Prof.Dr.Ion Stoia Centre of Rheumatic Diseases	5 Thomas Masaryk Street	Bucharest	
104RA202	ROMANIA	Chicea, Liana	Clinical County Hospital Sibiu	2-4 Corneliu Coposu Blvd	Sibiu	
104RA202	ROMANIA	Mociran, Eugenia	Emergency Clinical County Hospital Baia-Mare	31 Geroge Cosbuc Street	Baia Mare	
104RA202	ROMANIA	Ianuli, Adelina	Ianuli MedConsult	50 Nicolae Filipescu Street	Bucharest	
104RA202	RUSSIAN FEDERATION	Barbarash, Olga	Kemerovo Regional Clinical Hospital	Cardio-Rheumatology Department State Institution of Healthcare	Kemerovo	
104RA202	RUSSIAN FEDERATION	Chernykh, Tatyana Mikhalovna	Voronezh Regional Clinical Hospital #1	Rheumatology Department 151, Moskovskiy prospect	Voronezh	
104RA202	RUSSIAN FEDERATION	Glasunov, Alexandr Vladimirovich	City Clinical Hospital #81	Chair of therapy on the case o 10, Ulitsa Lobninskaya	Moscow	
104RA202	RUSSIAN FEDERATION	Konenkova, Ludmilla Petrovna	Scientific Institute of Clincial Immunology of Siberia	Rheumatology Department 14, Ulitsa Yadrintsovskaya	Novosibirsk	
104RA202	RUSSIAN FEDERATION	Stetsiouk, Olga Ulyanovna	Smolensk State Medical Academy of Roszdrav	Center of Clinical Trials and 28 Ulitsa Krupskoy	Smolensk	
104RA202	RUSSIAN FEDERATION	Nasonov, Evengy Lvovich	Russian Academy of Medical Sciences	Institute of Rheumatology 34a Kashirskoe shosse	Moscow	
104RA202	RUSSIAN FEDERATION	Shmidt, Eugeniya Isakovna	Moscow City Clinical Hospital #1	Rheumatology Department 10, Building 8	Moscow	
104RA202	RUSSIAN FEDERATION	Yakushin, Sergey Stepanovich	Ryazan Regional Clinical Cardilogical Dispensary	Cardiology Department 96 Ulitsa Stroikova	Rjazan	

Supplemental Methods Continued:

Study Number	Country	Investigator	Institution and IRB	Site Address	City	State
104RA202	RUSSIAN FEDERATION	Nikulenkova, Natalya	Vladimir State Regional Clincial Hospital	Rheumatology Department 41 Sudogodskoe shosse	Vladimir	
104RA202	RUSSIAN FEDERATION	Mazurov, Vadim	Saint Petersburg Medical Academy of Postgraduate Studies	Department of Therapy N1 (Rheu 41, Kirochnaya Street	Saint Petersburg	
104RA202	RUSSIAN FEDERATION	Sokolova, Ludmila	St. Petersburg State Healthcare: City Polyclinic 40	Department of Therapy 1st Floor	Saint Petersburg	
104RA202	RUSSIAN FEDERATION	Sorotskaya, Valentina	Tula Regional Hospital	Ulitsa Yablochkova, 1 a	Tula	
104RA202	RUSSIAN FEDERATION	Uspenskiy, Yuriy	St Petersburg State Medical Academy	Named after I I Mechnikov	St. Petersburg	
104RA202	RUSSIAN FEDERATION	Sokolova, Vera	North West Regional Medical Center of Roszdrav	Rhuematology Department Kodetskaya Liniya, 13-15	St.Petersburg	
104RA202	UNITED KINGDOM	Emery, Paul	Chapel Allerton Hospital	Academic Unit Of Muscular Dise 2nd Floor	Leeds	West Yorkshire
104RA202	UNITED KINGDOM	George, Emmanual	Arrowe Park Hospital	Rheumatology Dept Arrowe Park Road	Wirral	Cheshire
104RA202	UNITED KINGDOM	Mckenna, Frank	Trafford General Hospital	Rheumatology Department Davyhulme	Manchester	
104RA202	UNITED KINGDOM	Isaacs, John	Unversity of Newcastle upon Tyne	Department of Rheumatology 4th Floor Cookson Building	Newcastle upon Tyne	
104RA202	UNITED KINGDOM	Scott, David	King's College Hospital	Clinical and Academic Rheumato Denmark Hill	London	

Supplemental Methods Continued:

RA203 Investigators

Study Number	Country	Investigator	Institution and IRB	Site Address	City	State
104RA203	BELGIUM	Malaise, Michel	Centre Hospitalier Universitaire	Department of Rheumatology Domaine Universitaire du Sart Tilman	Liege	
104RA203	BELGIUM	Williame, Lucas	Academisch Ziekenhuis Middelheim	Lindendreef 1	Antwerp	
104RA203	BELGIUM	Durez, P	University Hospital Erasme	Department of Neurology Route de Lennik 808	Brussels	
104RA203	CANADA	Chow, Andrew	Credit Valley Hospital	2300 Eglinton Avenue West	Mississauga	Ontario
104RA203	CANADA	Grant, Eric	St. John Regional Hosptial	Department of Medicine 400 University Avenue	St. John	New Brunswick
104RA203	CANADA	Khraishi, Majed	St. Claire's Mercy Hospital	Le Merchant Road	St. John's	Newfoundland and Labrador
104RA203	CANADA	Russell, Anthony	University of Alberta	Rheumatic Disease Unit 562 Heritage Building	Edmonton	Alberta
104RA203	CANADA	Thorne, Carter	The Arthritis Program Research Group, Inc.	43 Lundy's Lane	Newmarket	Ontario
104RA203	CANADA	Rodrigues, Jude	Clinical Research & Arthritis Centre	1720 Howard Avenue	Windsor	Ontario
104RA203	CANADA	McCarthy, Timothy	Manitoba Clinic	790 Sherbrook Street	Winnipeg	Manitoba
104RA203	UNITED KINGDOM	Emery, Paul	Leeds University	Department of Rheumatology Research Unit	Leeds	
104RA203	UNITED KINGDOM	Mckenna, Frank	Trafford General Hospital	Rheumatology Department Davyhulme	Manchester	
104RA203	UNITED KINGDOM	Isaacs, John	Unversity of Newcastle upon Tyne	Department of Rheumatology 4th Floor Cookson Building	Newcastle upon Tyne	
104RA203	UNITED KINGDOM	Scott, David	King's College Hospital	Clinical and Academic Rheumato Denmark Hill	London	
104RA203	UNITED KINGDOM	Sheeran, Tom	Cannock Chase Hospital	Dept. of Rheumatology Brunswick Road	Cannock	
104RA203	UNITED KINGDOM	Chattopadhyay, Chandrabhusan	Wrightington Hospital	Clinical Trials Department Hall Lane	Wigan	
104RA203	UNITED STATES	Baldassare, Andrew	Arthritis Consultants	522 N. Ballas Road	St. Louis	Missouri
104RA203	UNITED STATES	Fiechtner, Justus	Fiechtner Research Inc.	3394 E. Jolly Road	Lansing	Michigan
104RA203	UNITED STATES	Alloway, Jeff	Physicians East, PA	2780-D Dickson Avenue	Greenville	North Carolina
104RA203	UNITED STATES	Busch, Howard	Family Arthritis Center	1025 Military Trail	Jupiter	Florida
104RA203	UNITED STATES	Caldron, Paul	Arizona Arthritis and Rheumatology Associates, PLLC	10599 North Tatum Blvd.	Paradise Valley	Arizona
104RA203	UNITED STATES	Chubick, Andrew	Arthritis Center of Texas	712 N. Washington Ave.	Dallas	Texas
104RA203	UNITED STATES	Codding, Christine	Health Research of Oklahoma	1211 N. Shartel	Oklahoma City	Oklahoma
104RA203	UNITED STATES	Cohen, Stanley	Metroplex Clinical Research Center	8144 Walnut Hill Lane	Dallas	Texas
104RA203	UNITED STATES	Furie, Richard	NJ-LIJ Health Systems	Division of Rheumatology & All Division of Rheumatology & Allergy-Clinical Immunology	Lake Success	New York
104RA203	UNITED STATES	Gaylis, Norman	Arthritis and Rheumatic Disease Specialties	2845 Aventura Boulevard	Aventura	Florida
104RA203	UNITED STATES	Genovese, Mark	Stanford University	Medical Center, Rheumatology	Palo Alto	California
104RA203	UNITED STATES	Greenwald, Maria	Osteoporosis Medical Center	39700 Bob Hope Drive	Rancho Mirage	California
104RA203	UNITED STATES	Kremer, Joel	The Center for Rheumatology	1367 Washington Avenue	Albany	New York
104RA203	UNITED STATES	Luggen, Michael	Cincinnati Rheumatic Disease Study Group	311 Straight Street	Cincinnati	Ohio
104RA203	UNITED STATES	Mease, Phillip	Seattle Rheumatology Associates	1101 Madison Street	Seattle	Washington
104RA203	UNITED STATES	Rigby, William	Dartmouth Hitchcock Medical Center	One Medical Center Drive	Lebanon	New Hampshire
104RA203	UNITED STATES	Ruderman, Eric	Northwestern University	Division of Rheumatology	Chicago	Illinois
104RA203	UNITED STATES	Shergy, William	Rheumatology Associates	201 Sivley Road	Huntsville	Alabama
104RA203	UNITED STATES	Fife, Rose	Indiana University	School of Medicine Outpatient Clinical Research Facility	Indianapolis	Indiana
104RA203	UNITED STATES	Saadeh, Constantine	Amarillo Center for Clinical Research	6842 Plum Creek Dr.	Amarillo	Texas
104RA203	UNITED STATES	Kavanaugh, Arthur	University of California,San Diego	Division of Rheumatology 9320 Campus Point Drive	La Jolla	California
104RA203	UNITED STATES	Dao, Kathryn	Texas Health Research and Education Institute	9301 N. Central Expressway	Dallas	Texas
104RA203	UNITED STATES	Murphy, Frederick	Altoona Osteoarthritis and Rheumatoid Arthritis Center	175 Meadowbrook Lane	Duncansville	Pennsylvania
104RA203	UNITED STATES	Berney, Seth	LSU Health Science Center	Center for Excellence for Arth 1501 Kings Highway	Shreveport	Louisiana
104RA203	UNITED STATES	Wolfe, Sanford	Stat Research	One Elizabeth Place	Dayton	Ohio
104RA203	UNITED STATES	Warrick, Jay	Rheumatology Consultants	4707 Papermill Drive	Knoxville	Tennessee
104RA203	UNITED STATES	Birbara, Charles	Clinical Pharmacology Study Group	26 Queen Street	Worcester	Massachusetts
104RA203	UNITED STATES	Forstot, Joseph	RASF Clinical Research Center	1050 NW 15th Street	Boca Raton	Florida
104RA203	UNITED STATES	Freeman, Pamela	Rheumatalogy Associates of Central Florida	3160 Southgate Commerce Blvd.	Orlando	Florida
104RA203	UNITED STATES	Wei, Nathan	Arthritis & Osteoporosis Center of Maryland	71 Thomas Johnson Drive	Frederick	Maryland
104RA203	UNITED STATES	Lies, Richard	Wichita Clinic	3311 E. Murdock	Wichita	Kansas
104RA203	UNITED STATES	Kolba, Karen	Pacific Arthritis Center Medical Group	607 E. Plaza Dr.	Santa Maria	California
104RA203	UNITED STATES	Schechtman, Joy	Sun Valley Arthritis Center, Ltd.	6818 W. Thunderbird Rd.	Peoria	Arizona
104RA203	UNITED STATES	Chase, Walter	Capital Medical Clinic	1301 West 38th Street	Austin	Texas
104RA203	UNITED STATES	Roschmann, Robert	Rheumatology, PC	2490 S. 11th Street	Kalamazoo	Michigan
104RA203	UNITED STATES	Hou, Antony	Boling Clinical Trials	510 N. 13th Avenue	Upland	California
104RA203	UNITED STATES	Weitz, Michael	Center for Arthritis and Rheumatic Diseases	6150 Sunset Drive	South Miami	Florida
104RA203	UNITED STATES	Miniter, Michael	Quad City Rheumatology, S.C.	4362 - 7th Street	Moline	Illinois
104RA203	UNITED STATES	Wellborne, Frank	Houston Institute for Clinical Research	7777 Southwest Freeway	Houston	Texas
104RA203	UNITED STATES	Sherrer, Yvonne	Center for Rheumatology Immunology and Arthritis	5333 North Dixie Highway	Fort Lauderdale	Florida
104RA203	UNITED STATES	Johnson, David	Bluegrass Internal Medicine	1000 Breckenridge Street	Owensboro	Kentucky

Supplemental Table 1:

Demographics of the Patients in the RA Studies and the SLE Registry

Clinical Characteristics of RA Study Patients

	TNF-IR*		DMARD-IR					
**Variable**	**Placebo**	**200 mg eow**	**Placebo**	**5 mg eow**	**70 mg eow**	**200 mg eow**	**70 mg monthly**	**200 mg monthly**
n	38	76	79	78	78	78	39	39
age years, mean (range)	54.3 (30-74)	53.6 (24-74)	52.3 (23-72)	50.3 (19-77)	49.9 (25-75)	49.9 (27-71)	51.9 (23-73)	48.1 (25-72)
% white	82	83	94	86	85	86	92	85
% female	82	80	86	86	86	85	87	90
Disease Duration years	9.3 (7.7)	12.9 (10.5)	7.2 (8.3)	7.8 (7.1)	7.3 (6.2)	8.3 (7.7)	8.4 (6.2)	6.3 (6.2)
SJC28	11.5 (6.2)	11.6 (6.5)	11.5 (6.1)	12.8 (5.0)	12.9 (5.6)	11.5 (4.9)	12.9 (5.0)	13.5 (5.7)
TJC28	14.6 (7.0)	15.2 (8.0)	17.5 (7.1)	17.3 (4.9)	20.0 (5.6)	14.6 (6.2)	17.8 (6.8)	17.4 (6.2)
CRP mg/liter	1.85 (1.6)	1.7 (2.2)	1.9 (2.7)	2.3 (2.2)	1.8 (2.2)	1.9 (2.2)	1.7 (1.4)	1.9 (1.9)
ESR mm/hr	42.0 (23.9)	45.1 (26.3)	48.9 (25.4)	58.3 (31.2)	55.5 (27.0)	48.8 (23.4)	51.6 (26.5)	49.3 (29.7)
DAS28 ESR	6.4 (1.1)	6.4(1.1)	6.7 (0.8)	6.8 (0.9)	6.9 (0.7)	6.6 (0.7)	6.8 (0.8)	6.7 (0.9)
HAQ-DI	1.5 (0.7)	1.6 (0.6)	1.6 (0.5)	1.6 (0.5)	1.5 (0.6)	1.7 (0.6)	1.6 (0.6)	1.6 (0.7)
Rf % Positive	63	61	78	77	82	81	74	87
Anti-CCP % Positive	66	66	80	79	79	81	79	82

*This study was terminated slightly early due to lack of efficacy in the DMARD-IR study. Of the 114 patients enrolled, 81 completed the full 14 weeks of dosing. Unless noted, mean and SD

Clinical Characteristics of SLE Study Patients

**Variable**	
n	292
age, mean (s	46 (11.9)
% Female	91.1
Ethnic Origin	
African-Am	33.90%
Caucasian	58.90%
Other	7.20%
SLEDAI ≥2	29%
Anti-DNA ≥1(	22%
C3 > 79	11%
C4 < 12	10%
Proteinuria >	11%

Supplemental Table 2

Measurement of IFN signatures

a). Genes used to form the Interferon transcriptional signature scores. The 8 gene IFN signature was a subset of the genes defined by Waddell et al (1) and exhibited internal correlation within these datasets. The 2 gene GBP1/2 signature was described by Rose et al (2) Asterisk indicates two or more separate primers.

Interferon 15 Gene Affymetrix	Interferon 3 gene qPCR	Interferon-gamma 8 gene Affymetrix	Interferon-gamma 2 gene Affymetrix
BATF2	ISG15	ASPH	GBP1*
DDX58	OAS1	CEBPA	GBP2
HERC5	Ly6E	CIITA	
IFI6		CLEC10A	
IFI44L		FCGR1A	
IFIT3		IL6R	
IFI44		LIMK2	
ISG15		SLC1A5	
LIPA			
MX1			
OAS1			
OAS3			
RSAD2 *			
TIMM10			
UBE2L6			

1). Waddell, S. J., S. J. Popper, K. H. Rubins, M. J. Griffiths, P. O. Brown, M. Levin, and D. A. Relman. 2010. Dissecting interferon-induced transcriptional programs in human peripheral blood cells. *PLoS One* 5: e9753.

2). Hall, J. C., L. Casciola-Rosen, A. E. Berger, E. K. Kapsogeorgou, C. Cheadle, A. G. Tzioufas, A. N. Baer, and A. Rosen. 2012. Precise probes of type II interferon activity define the origin of interferon signatures in target tissues in rheumatic diseases. *Proc Natl Acad Sci U S A* 109: 17609-17614.

Supplemental Table 3:

Incidence of adverse Events by Preferred Term for the combined Placebo-Controlled Studies		
**Variable**	**Placebo**	**Baminercept**
number of subjects dosed	117 (100)	388 (100)
Events	67 (57)	234 (60)
Rheumatoid Arthritis	21 (18)	35 (9)
Headache	8 (7)	27 (7)
URTI	2 (2)	23 (6)
Nausea	7 (6)	19 (5)
Cough	5 (4)	18 (5)
Pyrexia	0 (0)	17 (4)
Influenza	2 (2)	16 (4)
Myalgia	3 (3)	14 (4)
Nasopharyngitis	3 (3)	14 (4)
Diarrhoea	6 (5)	13 (3)
Injection Site Eryhema	0 (0)	12 (3)
Injection Site Pain	4 (3)	12 (3)
Dizziness	4 (3)	10 (3)
RTI	2 (2)	10 (3)
Chills	2 (2)	9 (2)
Hypertension	4 (3)	9 (2)
Fatigue	3 (3)	8 (2)
Bronchitis	1 (<1)	7 (2)
Injection Site Puritis	1 (<1)	7 (2)
Migrane	2 (2)	7 (2)
ALT Increased	1 (<1)	6 (2)
AST Increased	0 (0)	6 (2)
Asthenia	1 (<1)	6 (2)
Dyspepsia	1 (<1)	6 (2)
Vomiting	0 (0)	6 (2)
Arthralgia	1 (<1)	5 (1)
Increased Body Temperature	1 (<1)	5 (1)
Leukocytosis	0 (0)	5 (1)
Abdominal Pain Upper	1 (<1)	4 (1)
Back Pain	0 (0)	4 (1)
Gastritis	1 (<1)	4 (1)
Injection Site Haematoma	1 (<1)	4 (1)
Injection Site Rash	0 (0)	4 (1)
Insomnia	1 (<1)	4 (1)
Oedema Perripheral	0 (0)	4 (1)
Rash	2 (2)	4 (1)
Rhinitis	0 (0)	4 (1)
UTI	5 (4)	4 (1)

Numbers in parentheses are percentages.
A subject was counted only once within each preferred term

Supplemental Table 4:

Lack of an appreciable effect of baminercept treatment on ACR scores as assessed at 14 weeks.

Table 3: Clinical ACR Responses at 14 Weeks						
		DMARD-IR			TNF-IR	
	ACR 20	ACR50	ACR70	ACR 20	ACR 50	ACR 70
Placebo	32	11	4	13	5	3
5 mg q2w	36	14	4			
70 mg q2w	40	14	8			
200 mg q2w	32	12	6	14	11	1
70 mg monthly	36	8	3			
200 mg monthly	38	18	10			

In TNF-IR, numbers included all 114 patients who started dosing and missing data were handled using non-responder imputation methodology. Of these 114 subjects, 81 (71%) completed the entire treatment period with the majority of the discontinuations due to early trial termination. None of the effects of baminercept treatment on ACR scores reached significance, Cochran-Mantel-Haenszet Test.

Supplemental Figure 1

Comparison of IFN signatures in RA and SLE

a). Comparison of the baseline numerical 15-gene microarray IFN score in normal, DMARD-IR, TNF-IR and SLE patients.

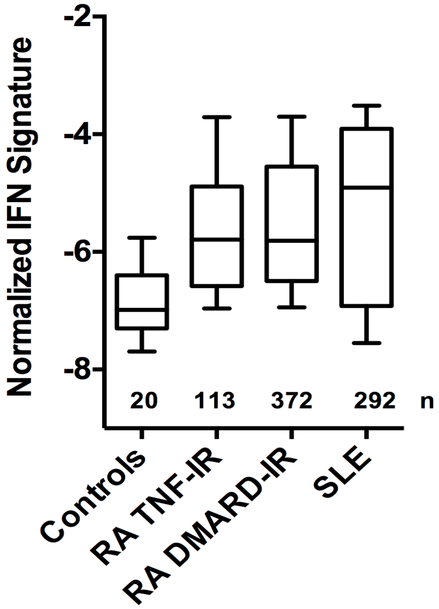

Supplemental Figure 2:

Elevation of serum LIGHT levels in RA patients following baminercept treatment

All baminercept doses greater than 5 mg q2w led to a similar elevation of serum LIGHT (Tnfsf14) levels. LIGHT levels were similarly elevated at 6 weeks of treatment (not shown). Prior pilot work had shown that LIGHT levels were elevated about 1.5 fold in most RA patients with a mean of 140 pg/ml in rough agreement with prior published data for arthritis patients (1-2). In our DMARD-IR and TNF-IR studies, the mean values at baseline were slightly lower. Serum levels were determined using a commercial ELISA kit (R&D Systems) where the lower limit of quantitation was 16-30 pg/ml. The assay is complicated by two factors, first, the presence of baminercept partially interferes with LIGHT detection such that the actual values could be as much as 20-30 fold higher. Second, the natural soluble decoy receptor, DcR3, which has been observed in blood from RA patients (3, completely blocks the ability of the ELISA to detect LIGHT (tested using recombinant DcR3-Fc fusion protein, although the bivalent DcR3-Fc may not reflect the presumably monovalent natural form). Baminercept has a much higher affinity for LIGHT and can displace DcR3-Fc from LIGHT thus rendering it detectable by the ELISA. Therefore, the elevation in LIGHT levels could result from both complexation with the baminercept Fc fusion protein and extension of the serum half-life as well as displacement from potential DcR3-LIGHT complexes in circulation at baseline. Box and whiskers show 10-90% range.

1). Edwards, J. R., S. G. Sun, R. Locklin, C. M. Shipman, I. E. Adamopoulos, N. A. Athanasou, and A. Sabokbar. 2006. LIGHT (TNFSF14), a novel mediator of bone resorption, is elevated in rheumatoid arthritis. *Arthritis Rheum* 54: 1451-1462.

2). Chandran, V., R. J. Cook, J. Edwin, H. Shen, F. J. Pellett, S. Shanmugarajah, C. F. Rosen, and D. D. Gladman. 2010. Soluble biomarkers differentiate patients with psoriatic arthritis from those with psoriasis without arthritis. *Rheumatology (Oxford)* 49: 1399-1405

3). Hayashi, S., Y. Miura, K. Tateishi, M. Takahashi, and M. Kurosaka. 2010. Decoy receptor 3 is highly expressed in patients with rheumatoid arthritis. *Mod Rheumatol* 20: 63-68.

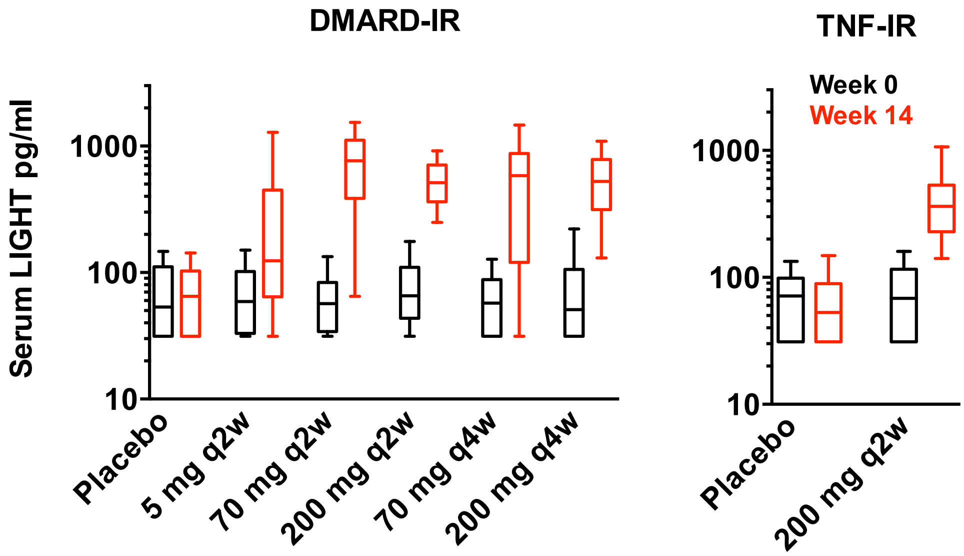

Supplemental Figure 3:

Characteristics of the IFN signatures observed in this study.

a). Lack of correlation between the 15 Gene conventional IFN and the 8 gene type II IFN signatures in RA and SLE patients (Spearman rank r). It is not clear if the differences in absolute type II IFN signatures between diseases are due to technical normalization issues.

b). Good correlation between the 15 Gene conventional IFN and a 2 gene (GBP1/2) IFN signature in RA and SLE patients.

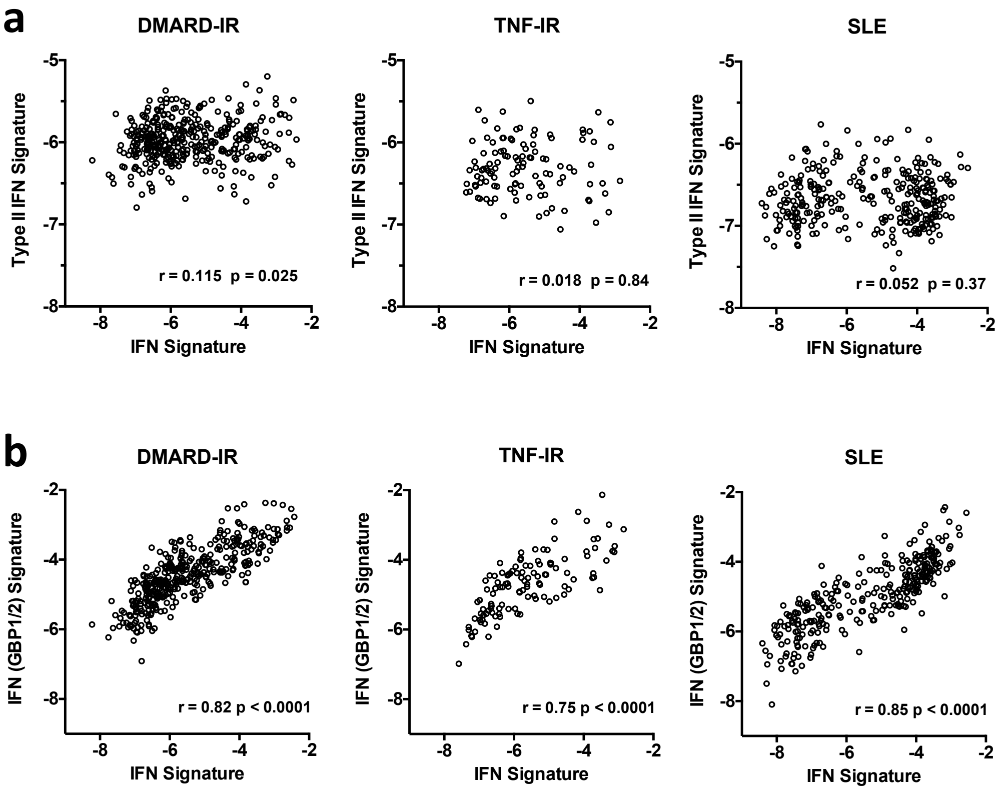

Supplemental Figure 4:

Characteristics of baminercept induced lymphocytosis in RA patients

Percent change at week 14 in total white blood cells (WBC), lymphocyte and monocyte numbers increased with baminercept treatment, while neutrophil, basophil and eosinophil counts were relatively unchanged or shifted significantly in only the DMARD-IR study. In the DMARD-IR study, patients treated with 70 or 200 mg q2w were pooled to form the "Bam" group.

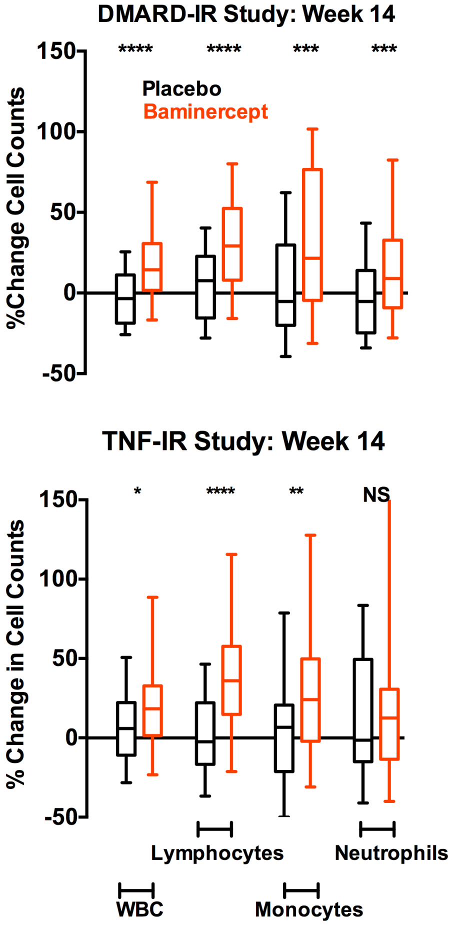

Supplemental Figure 5:

Relationship between IFN signature and baminercept treatment on blood neutrophil and monocyte counts

(a). Some decrease in neutrophil counts in IFN signature high patients only in the DMARD-IR study. Baminercept treatment did not appreciably alter the neutrophil counts. Significance was determined with the Mann-Whitney test. In the DMARD-IR study, data from patients treated with 70 or 200 mg q2w were pooled to form the "Bam" group.

(b). Monocyte cell counts were slightly lower in IFN signature high patients in the DMARD-IR group. Baminercept treatment elevated monocyte numbers patients with both high and low IFN signatures.

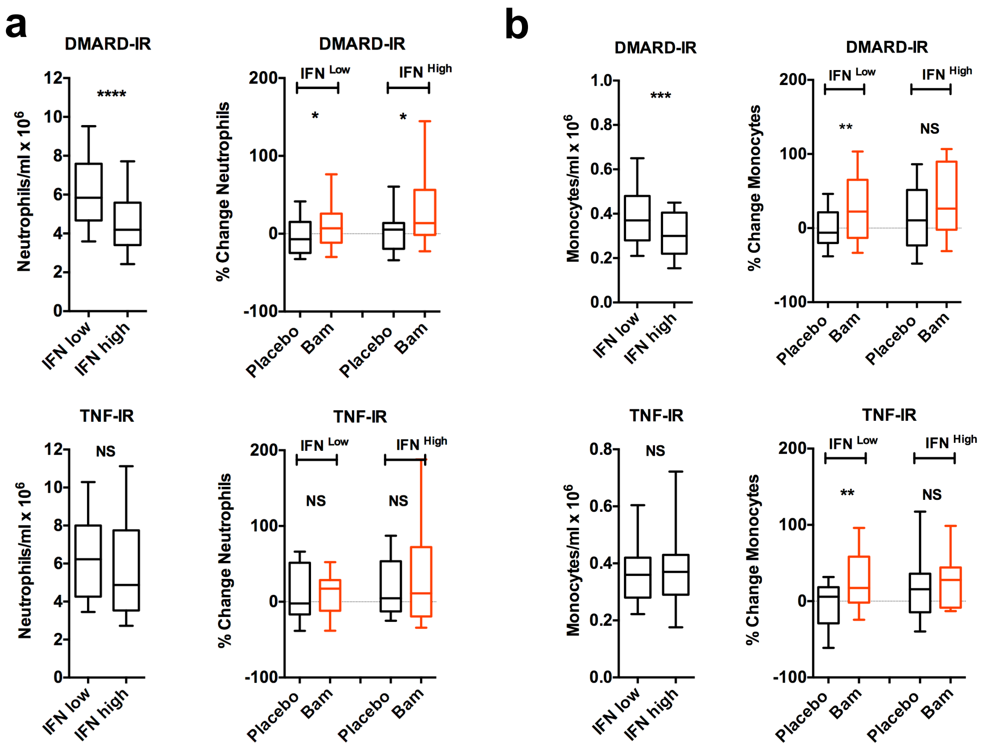

Supplemental Figure 6:

FACS analysis of the effects of baminercept treatment on peripheral blood lymphocyte subsets

FACS analysis was performed on a subset of the patients. Data are consistent with increased numbers of circulating T and B cells with the CD4+ T cells contributing more than the CD8+ T cells. CD16+, CD56+ NK cells decreased slightly in both studies in agreement with the qPCR analysis. Starting with whole blood, RBCs were lysed and the cells stained for the conventional markers. FACS counting beads were used to obtain absolute cell counts. Data are plotted as means and SEM of the absolute cell numbers and significance was determined with the Mann-Whitney test. FACS analyses were done at 2 central centers.

Given the limited nature of the data, we combined all of the patients treated with high dose baminercept (70 or 200 q2w cohorts) from both studies. Data included 20 placebo, 8 70 mg q2w and 16 200 mg q2w patients (DMARD-IR) and 7 placebo and 15 200 mg q2w patients (TNF-IR). Not all patients had FACS analyses from all three time points, among the 27 placebo treated patients, 66-68 out of 81 possible analyses (depending on the marker cocktail) and from the 39 baminercept treated patients 94-95 out of 117 possible analyses were complete.

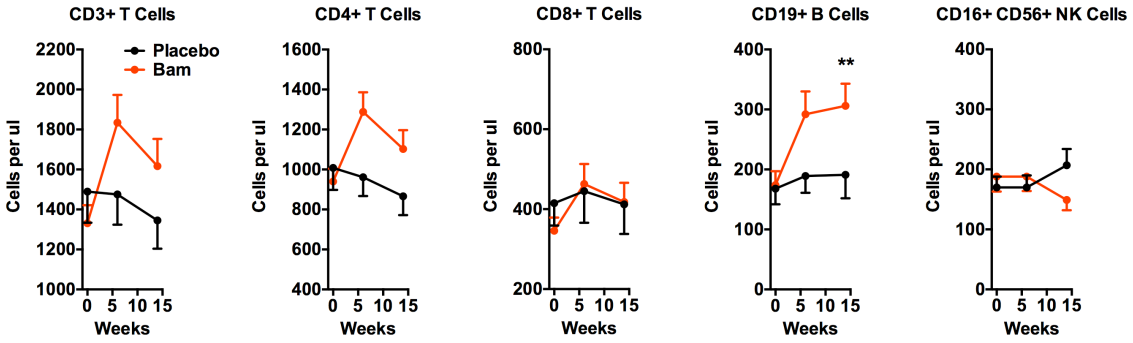

Supplemental Figure 7:

qPCR analysis on whole blood RNA of the effects of baminercept treatment on gene markers of myeloid subsets.

Expression of genes specific for myeloid subsets as defined by Abbas et al and Bennett et al or the Immunological Genome database: immature neutrophils, Defensin A3; plasmacytoid DC, CLEC4C (BDCA2) and LILRA4 (ILT7) and Dendric cells, CD1E: monocytes and DC, SPARC and activated monocytes SLAMF7.   Baminercept treatment in the DMARD-IR study included only the 70 and 200 mg q2w cohorts. Levels of Defensin A3 RNA decreased which may have been an artificial consequence of baminercept induced lymphocytosis. Likewise, ILT7 (pDC) increased with treatment potentially consistent with the general baminercept induced lymphocytosis (change in ILT7 correlated with the change in lymphocyte counts, Spearman r = 0.34). The lack of a parallel change in CLEC4C is not understood. Significance was determined with a Mann-Whitney test.

Abbas, A. R., K. Wolslegel, D. Seshasayee, Z. Modrusan, and H. F. Clark. 2009. Deconvolution of blood microarray data identifies cellular activation patterns in systemic lupus erythematosus. *PLoS One* 4: e6098

Bennett, L., A. K. Palucka, E. Arce, V. Cantrell, J. Borvak, J. Banchereau, and V. Pascual. 2003. Interferon and granulopoiesis signatures in systemic lupus erythematosus blood. *J Exp Med* 197: 711-723

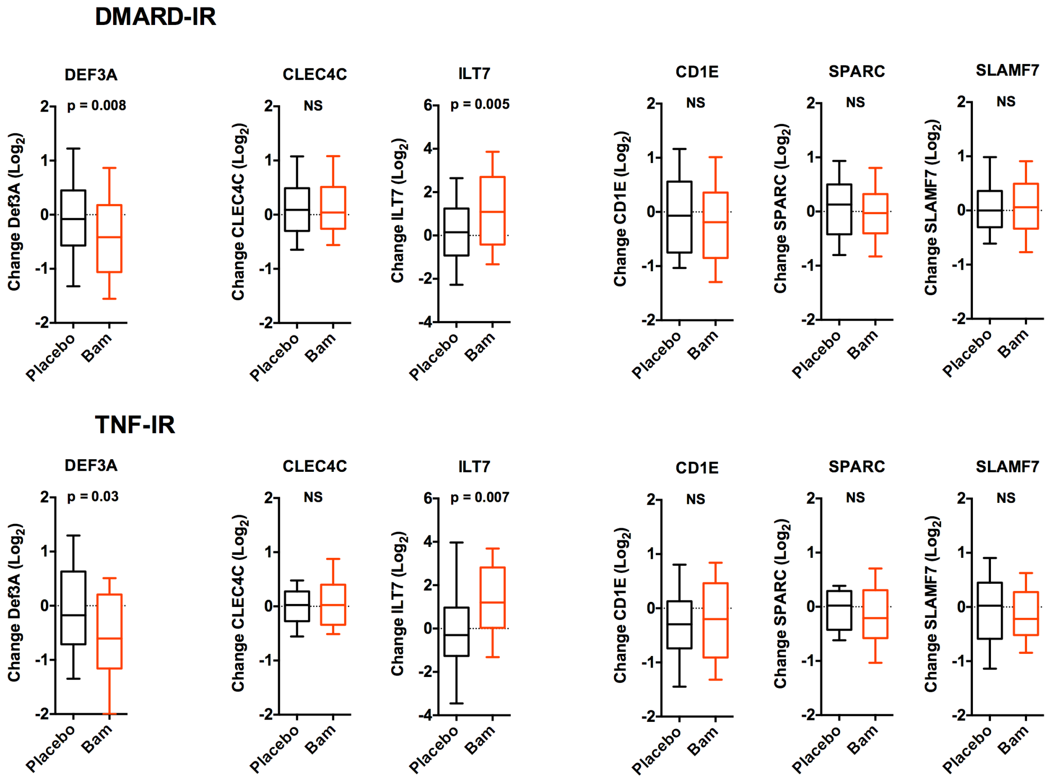

Supplemental Figure 8:

Effects of baminercept treatment on the Swollen Joint Count 28 (SJC28) scores in the DMARD-IR and TNF-IR studies.

(a) Baminercept treatment resulted in a trend towards reduced SJC28 numbers in the both studies. Statistical significance was determined with a two-tailed Mann-Whitney Test. In the DMARD-IR study, patients treated with 70 or 200 mg q2w were pooled. Patients were excluded if the baseline SJC28 was less than 8 (study inclusion criterion was 8 or more SJC using the 66 joint score). The number of patients in each group is indicated.

(b) Effects of baminercept treatment with either low or high baseline IFN signatures. IFN status was defined as a qPCR IFN score or less than (low) or greater (high) than 1.0. Data were pooled from the 70 and 200 mg q2w cohorts with the same baseline SJC28 greater than 7 requirement as above. The number of patients in this analysis were IFN low placebo 40, baminercept 82 and IFN high placebo 16, baminercept 32. The corresponding TNF-IR study was underpowered for this particular analysis.

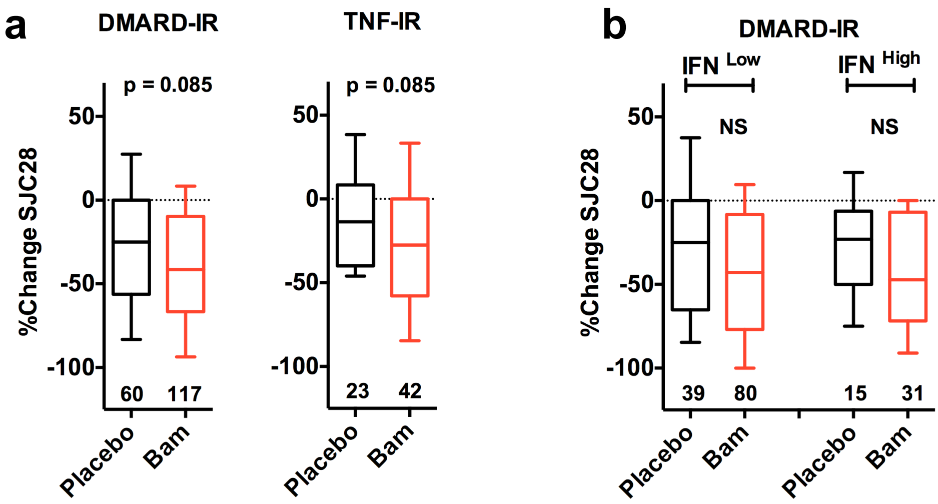

Supplemental Figure 9:

Little effect of baminercept treatment on CRP levels and Erythrocyte Sedimentation Rates (ESR) in the DMARD-IR and TNF-IR studies.

(a) Changes in CRP values following 14 weeks of baminercept treatment. Statistical significance was determined with a Mann-Whitney test. In the DMARD-IR study, patients treated with 70 or 200 mg q2w were pooled and patients with baseline cRP values below 0.1 mg/L were excluded. Given the disruptive effects on percent change of low baseline values, the data were also analyzed for the impact of baminercept on patients with starting values of CRP greater than 1.5 mg/dl. The number of patients in each group is indicated.

(b) Changes in ESR values following 14 weeks of baminercept treatment as assessed as above. High ESR values were greater than 40 mm/hr.

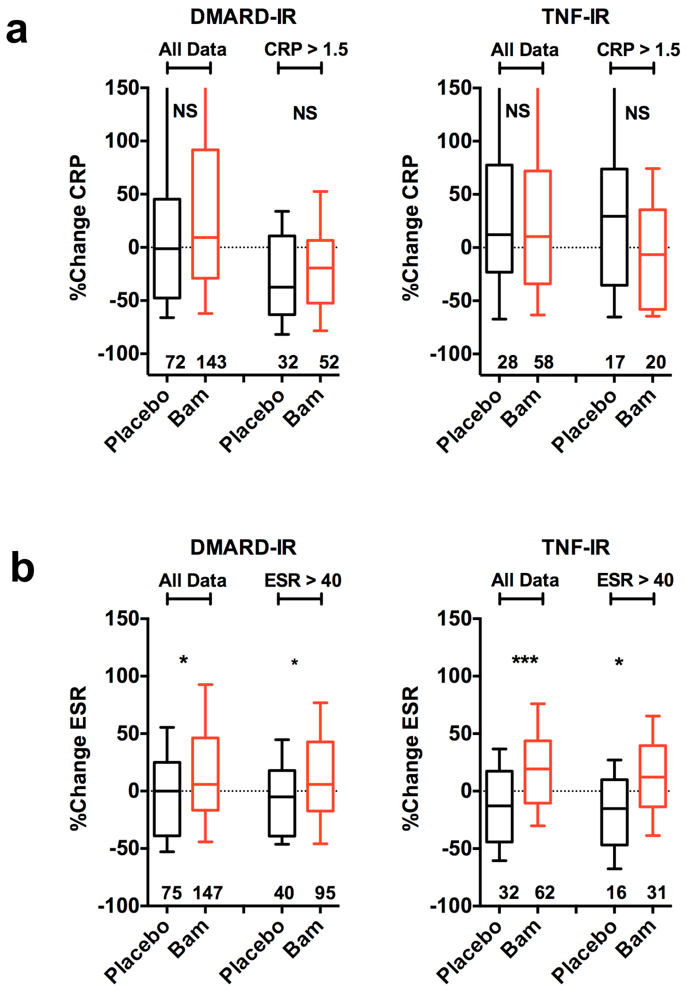

Supplemental Figure 10:

The IFN signature status in RA patients does not correlate with clinical or serological parameters.

The baseline 3 gene qPCR score is plotted vs the baseline clinical parameters of CRP, ESR, SJC28, DAS28 ESR, rheumatoid factor (Rf) titer and anti-CCP status for the patients in the DMARD-IR study (placebo, 70 and 200 mg q2w) and all patients from the TNF-IR study. Results did not change with individual analysis of each study. In this analysis of the DMARD-IR and TNF-IR patients, 80% and 65% (respectively) were CCP antibody positive.

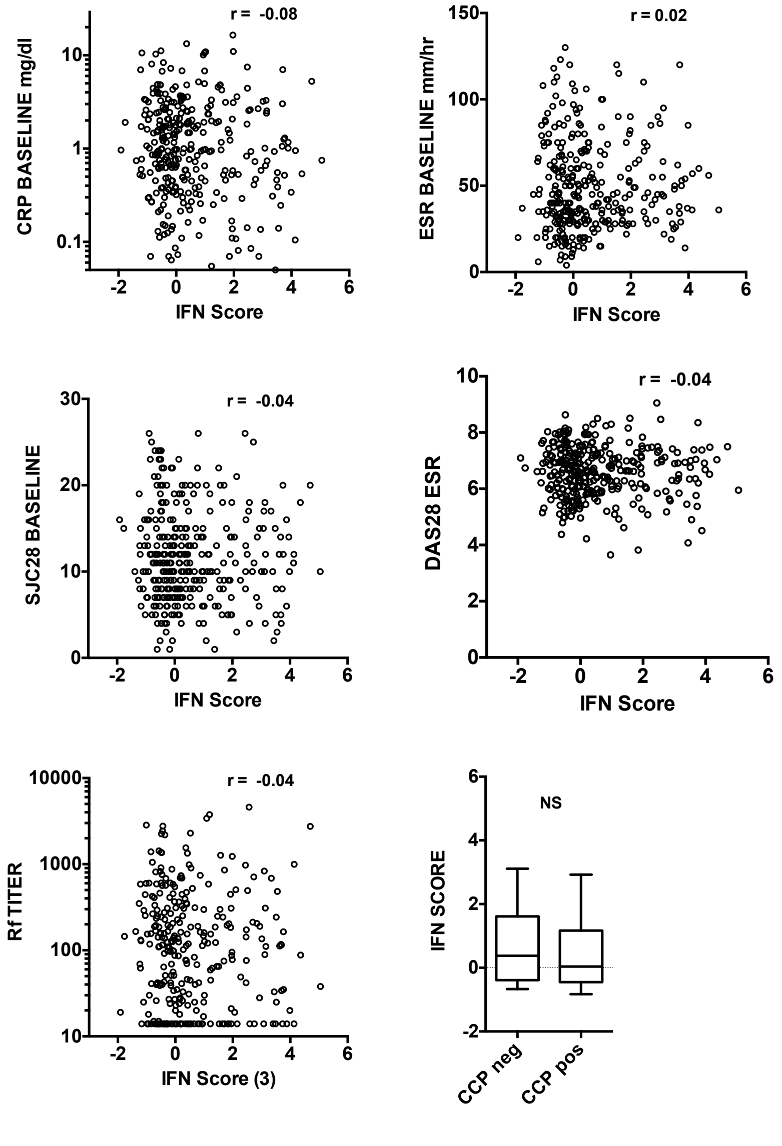

Supplement: File S1 — Supporting files. Supplemental Methods and Investigator Listing. Supplemental Table 1, Demographics of the Patients in the RA studies and the SLE registry. Supplemental Table 2, Measurement of IFN signatures. Supplemental Table 3, Incidence of adverse events by preferred term for the combined placebo-controlled studies. Supplemental Table 4, Lack of an appreciable effect of baminercept treatment on ACR scores as assessed at 14 weeks. Supplemental Figure 1, Comparison of IFN signatures in RA and SLE. Supplemental Figure 2, Elevation of serum LIGHT levels in RA patients following baminercept treatment. Supplemental Figure 3, Characteristics of the IFN signatures observed in this study. Elevation of serum LIGHT levels in RA patients following baminercept treatment. Supplemental Figure 4, Characteristics of baminercept induced lymphocytosis in RA patients. Supplemental Figure 5, Relationship between IFN signature and baminercept treatment on blood neutrophil and monocyte counts. Supplemental Figure 6, FACS analysis of the effects of baminercept treatment on peripheral blood lymphocyte subsets. Supplemental Figure 7, Quantitative PCR analysis on whole blood RNA of the effects of baminercept treatment on gene markers of myeloid subsets. Supplemental Figure 8, Effects of baminercept treatment on the Swollen Joint Count 28 (SJC28) scores in the DMARD-IR and TNF-IR studies. Supplemental Figure 9, Little effect of baminercept treatment on CRP levels and Erythrocyte Sedimentation Rates (ESR) in the DMARD-IR and TNF-IR studies. Supplemental Figure 10, The IFN signature status in RA patients does not correlate with clinical or serological parameters. (PDF) [file pone.0112545.s001.pdf]
