# Supplementary material for: Lymphotoxin-LIGHT Pathway Regulates the Interferon Signature in Rheumatoid Arthritis
Source: PLoS One. 2014 Nov 18;9(11):e112545. doi: 10.1371/journal.pone.0112545 (PMC4236099; doi:10.1371/journal.pone.0112545)
Supplement: Protocol S1 — Trial Protocol. (PDF) [file pone.0112545.s003.pdf]

biogen idec

14 Cambridge Center
Cambridge, MA 02142, USA
[redacted]

5200 Research Place
San Diego, CA 92122, USA
[redacted]

Thames House
Foundation Park
Maidenhead Berkshire SL6 3UD
United Kingdom
[redacted]

PROTOCOL NUMBER: 104RA202

STUDY PHASE: 2b

PROTOCOL TITLE: A Phase 2b, Randomized, Double-Blind, Placebo-Controlled, Multicenter, Dose-Finding Study to Evaluate the Efficacy, Safety, Pharmacokinetics, and Pharmacodynamics of BG9924 When Given in Combination With Methotrexate to Subjects With Active Rheumatoid Arthritis Who Have Had an Inadequate Response to Conventional DMARD Therapy

EUDRA CT NO: 2006-005466-39

DATE: 22 January 2007
Version 2
FINAL

I approve this document in accordance with Biogen Idec's current policies and procedures.

Carmen Bozic MD

Carmen Bozic, VP, Global Head, Drug Safety and Risk Mgt., Biogen Idec Inc.

24 JANUARY 2007
Date

CONFIDENTIAL
The information contained herein may not be used, disclosed, or published without the written consent of Biogen Idec Inc.

TABLE OF CONTENTS

1 CONTACT LIST 7
2 LIST OF ABBREVIATIONS 8
3 SYNOPSIS 10
4 STUDY ACTIVITIES 104RA202 16
5 INTRODUCTION 19
5.1 Overview of Rheumatoid Arthritis 19
5.1.1 Rationale for Use of BG9924 in RA 19
5.2 Profile of Previous Experience 20
5.3 Study Rationale 21
5.4 Dose Rationale 22
6 OBJECTIVES 23
6.1 Primary Objective 23
6.2 Secondary Objectives 23
7 STUDY DESIGN 23
7.1 Study Overview 23
7.2 Overall Study Duration and Follow-Up 24
7.3 Study Stopping Rules 24
8 STUDY POPULATION 25
8.1 Inclusion Criteria 25
8.2 Exclusion Criteria 25
8.3 Screening Log 28
9 ENROLLMENT AND RANDOMIZATION PROCEDURES 28
9.1 Enrollment Procedures 28
9.2 Randomization Procedures 28
9.3 Blinding Procedures 28
10 STUDY TREATMENT DESCRIPTION AND ALLOCATION 29
10.1 BG9924 29
10.2 Comparator 30
10.3 Additional Protocol-Designated Products 30
10.4 Study Treatment Accountability 30
11 TREATMENT 31
11.1 Treatment Schedule 31
11.2 Removal of Subjects from Treatment and/or Study 31
11.2.1 Discontinuation of Treatment 31
11.2.2 Withdrawal of Subjects From the Study 32
11.3 Treatment Compliance 32

11.4 Concomitant Therapy and Procedures 32
11.5 Continuation of Treatment 34
12 EFFICACY, BG9924 CONCENTRATION, AND PHARMACODYNAMIC ASSESSMENTS 34
12.1 Clinical Efficacy Assessments 34
12.2 RA Disease Marker Assessments 36
12.3 BG9924 Concentration Assessments 36
12.4 Pharmacodynamic Assessments 37
12.5 Other Assessments 37
13 SAFETY ASSESSMENTS 38
13.1 Clinical Safety Assessments 38
13.2 Laboratory Safety Assessments 38
14 SCHEDULE OF EVENTS 38
14.1 Subject Management 39
14.2 Tests and Assessments 39
14.2.1 Screening Visit (Within 28 Days Prior to Day 0) 40
14.2.2 Visit 1 (Week 0, Day 0) 40
14.2.3 Visit 1a and 1b (Week 0, Days 2 and 5; visit only applicable to PK sub-study) 41
14.2.4 Visit 2 (Week 1, Day 7 ±2) 42
14.2.5 Visit 3 (Week 2, Day 14 ±2) 42
14.2.6 Visit 3a (Week 3, Day 21; visit only applicable to PK sub-study) 42
14.2.7 Visit 4 (Week 4, Day 28 ±3) 42
14.2.8 Visit 5 (Week 6, Day 42 ±3) 43
14.2.9 Visit 6 (Week 8, Day 56 ±3) 43
14.2.10 Visit 7 (Week 10, Day 70 ±3) 44
14.2.11 Visit 8 (Week 12, Day 84 ±3) 44
14.2.12 Visits 8a, 8b, and 8c (Week 12, Days 86, 89, and 91; visit only applicable to PK sub-study) 44
14.2.13 Visit 9 (Week 14, Day 98 ±3; primary endpoint evaluation, option to enter extension study, and early withdrawal visit [withdrawal prior to Week 14]) 44
14.2.14 Visit 10 (Week 18, Day 126 ±3) 45
14.2.15 Visit 11 (Week 22, Day 154 ±3) 46
14.2.16 Visit 12 (Week 26, Day 182 ±3; late withdrawal visit [withdrawal after Week 14]) 46
15 SAFETY DEFINITIONS, MONITORING, AND REPORTING 47
15.1 Definitions 47
15.1.1 Adverse Event 47

15.1.2 Serious Adverse Event....47
15.1.3 Suspected Unexpected Serious Adverse Reactions....48
15.2 Monitoring and Recording Events....48
15.2.1 Immediate Reporting of Serious Adverse Events....48
15.2.1.1 Death....48
15.3 Safety Classifications....49
15.3.1 Relationship of Events to Study Treatment....49
15.3.2 Severity of Events....50
15.4 Prescheduled or Elective Procedures or Routinely Scheduled Treatments....50
15.5 Procedures for Handling Special Situations....50
15.5.1 Medical Emergency....50
15.5.2 Pregnancy....50
15.5.3 Unblinding....51
15.5.3.1 Accidental Unblinding....51
15.5.3.2 Unblinding for Reporting Purposes....51
15.6 Investigator Responsibilities....51
15.7 Biogen Idec Responsibilities....52
16 STATISTICAL STATEMENT AND ANALYTICAL PLAN....52
16.1 Description of Objectives and Endpoints....52
16.1.1 Primary Objective and Endpoints....52
16.1.2 Secondary Objectives....53
16.1.3 Exploratory Endpoints....53
16.2 Criteria for the Endpoints....53
16.2.1 Demography and Baseline Disease Characteristics....53
16.2.2 Pharmacokinetics....53
16.2.2.1 Analysis Population....53
16.2.2.2 Methods of Analysis....54
16.2.3 Pharmacodynamics....54
16.2.3.1 Analysis Population....54
16.2.3.2 Methods of Analysis....54
16.2.4 Efficacy Data....54
16.2.4.1 Analysis Population....54
16.2.4.2 Methods of Analysis....55
16.2.5 Safety Data....56
16.2.5.1 Analysis Population....56
16.2.5.2 Methods of Analysis....57
16.2.6 Immunogenicity Data....58

16.2.6.1 Analysis Population 58
16.2.6.2 Methods of Analysis 58
16.3 Primary Analyses 58
16.4 Sample Size Considerations 58
17 ETHICAL REQUIREMENTS 59
17.1 Declaration of Helsinki 59
17.2 Ethics Committee 59
17.3 Subject Information and Consent 59
17.4 Subject Data Protection 60
18 ADMINISTRATIVE PROCEDURES 60
18.1 Study Site Initiation 60
18.2 Quality Assurance 60
18.3 Monitoring of the Study 61
18.4 Study Funding 61
19 FURTHER REQUIREMENTS AND GENERAL INFORMATION 61
19.1 External Service Organizations 61
19.1.1 Interactive Voice Response System 61
19.1.2 Remote Data Capture 61
19.1.3 Data Coordinating Center 61
19.1.4 Central Laboratories for Laboratory Assessments 61
19.2 Study Committees 63
19.2.1 Data Monitoring Committee 63
19.3 Changes to Final Study Protocol 63
19.4 Ethics Committee Notification of Study Completion or Termination 63
19.5 Retention of Study Data 63
19.6 Study Report Signatory 63
20 REFERENCES 65
21 SIGNED AGREEMENT OF THE STUDY PROTOCOL 66
22 APPENDIX A: ACR REVISED CRITERIA FOR THE CLASSIFICATION OF FUNCTIONAL CAPACITY IN RA 67
23 APPENDIX B: ACR CORE SET MEASUREMENTS 68
24 APPENDIX C: HEALTH ASSESSMENT QUESTIONNAIRE-DISABILITY INDEX 69
25 APPENDIX D: SHORT FORM 36 QUESTIONNAIRE 71
26 APPENDIX E: FUNCTIONAL ASSESSMENT OF CHRONIC ILLNESS THERAPY-FATIGUE QUESTIONNAIRE (VERSION 4) 75
27 APPENDIX F: OCULAR SURFACE DISEASE INDEX© (OSDI©) 78

28 APPENDIX G: WORLD MEDICAL ASSOCIATION DECLARATION OF HELSINKI 79

LISTING OF FIGURES

Figure 15-1. Reporting Information for SAEs......49

LISTING OF TABLES

Table 16-1. EULAR Criteria of Response......56
Table 16-2. Criteria to Determine Clinically Relevant Abnormalities in Vital Signs......58

1 CONTACT LIST

Biogen Idec Inc.
14 Cambridge Center
Cambridge, MA, 02142
USA

Biogen Idec Ltd.
Thames House
Foundation Park
Maidenhead, Berkshire SL6 3UD
United Kingdom

[redacted]

[redacted]

[redacted]

For SAE Reporting:

[redacted]

[redacted]

2 LIST OF ABBREVIATIONS

ACR	American College of Rheumatology
AUC	area under the curve
AE	adverse event
ALT	alanine aminotransferase
ANA	anti-nuclear antibody
aPTT	activated partial thromboplastin time
AST	aspartate aminotransferase
BAFF	B cell activation factor from the TNF family
CCP-2	cyclic citrullinated peptide-2
CIA	collagen-induced arthritis
Cl	clearance
C_{max}	maximum serum concentration
COMP	cartilage oligomeric matrix protein
CRF	Case Report Form
DAS28	Disease Activity Score, 28-joint version
DHA	Directions for Handling and Administration
DMARD	disease-modifying anti-rheumatic drug
DMC	Data Monitoring Committee
dsDNA	double-stranded DNA
ECG	electrocardiogram
ESR	erythrocyte sedimentation rate
EULAR	European League Against Rheumatism
FACS	fluorescence-activated cell sorter
FACIT-F	Functional Assessment of Chronic Illness Therapy-Fatigue
GC	germinal center
GCP	Good Clinical Practice
HAQ-DI	Health Assessment Questionnaire disability index
HBsAg	hepatitis B surface antigen
HBcAb	hepatitis B core antibody
HBsAb	hepatitis B surface antibody
HCV	hepatitis C virus
HIV	human immunodeficiency virus
HEV	high endothelial venule
hsCRP	high sensitivity C-reactive protein
ICAM	intracellular adhesion molecule
ICF	Informed Consent Form
ICH	International Conference on Harmonisation
IFN-γ	interferon-γ
Ig	immunoglobulin
IgA	immunoglobulin A
IgG	immunoglobulin G
IgG_1	immunoglobulin G type 1

CONFIDENTIAL
The information contained herein may not be used, disclosed, or published without the written consent of
Biogen Idec Inc.

IgM	immunoglobulin M
IL	interleukin
IM	intramuscular
INR	international normalized ratio
IV	intravenous
IVRS	interactive voice response system
LDH	lactate dehydrogenase
LOCF	last observation carried forward
LT	lymphotoxin
LTβR	lymphotoxin beta receptor
mLTβR-Ig	murine lymphotoxin beta receptor fusion protein
MMP3	matrix metalloproteinase 3
MTX	methotrexate
NSAID	non-steroidal anti-inflammatory drug
OSDI	Ocular Surface Disease Index
PD	pharmacodynamics
PHI	protected health information
PK	pharmacokinetics
PPD	purified protein derivative
PT	prothrombin time
QoL	quality of life
RA	rheumatoid arthritis
RBC	red blood cell
RDC	remote data capture
RF	rheumatoid factor
SAE	serious adverse event
SC	subcutaneous
SF-36	Short Form 36
SJC	swollen joint count
SUSAR	suspected unexpected serious adverse reactions
TB	tuberculosis
TJC	tender joint count
t_{max}	time to maximum serum concentration
TNF	tumor necrosis factor
ULN	upper limit of normal
VAS	visual analog scale
V	volume of distribution
WBC	white blood cell

3 SYNOPSIS

Protocol Number:	104RA202
Version Number:	1
Protocol Title:	A Phase 2b, Randomized, Double-Blind, Placebo-Controlled, Multicenter, Dose-Finding Study to Evaluate the Efficacy, Safety, Pharmacokinetics, and Pharmacodynamics of BG9924 When Given in Combination With Methotrexate to Subjects With Active Rheumatoid Arthritis Who Have Had an Inadequate Response to Conventional DMARD Therapy
Study Phase:	2b
Rationale for the Study:	This Phase 2b study is designed to evaluate the efficacy and safety of 5 different BG9924 subcutaneous (SC) dose regimens versus placebo over a 12-week treatment period, including a loading dose. Eligible subjects are those with active rheumatoid arthritis (RA) and who have previously had an inadequate response to treatment with conventional disease-modifying anti-rheumatic drug (DMARD) therapy. This study will assist with dose selection for further planned clinical studies of BG9924.
Study Design:	This is a randomized, double-blind, placebo-controlled, multicenter, dose-finding study.
Study Location:	South America, Europe, Russia, UK, and other countries
Study Objectives:	*Primary*:<br>To evaluate the efficacy of 5 dose regimens of BG9924 when administered in combination with methotrexate (MTX) to subjects with active RA who have had an inadequate response to conventional DMARD therapy<br><br>*Secondary:*<br>• To assess the safety and tolerability of these 5 dose regimens of BG9924 in this patient population.<br>• To assess the pharmacokinetic (PK) and pharmacodynamic (PD) profile of these 5 dose regimens of BG9924 in this patient population.

Number of Planned Subjects:	Approximately 380 subjects
Study Population:	Subjects 18 to 75 years of age, inclusive, with active RA who have had an inadequate response to conventional DMARD therapy.
Treatment Groups:	Subjects will be randomized to receive BG9924 or placebo by SC injection every other week for 12 weeks. All subjects will receive a loading dose as described below. Subjects will be randomized at a ratio of 2:2:2:2:1:1. The following dose regimens will be included:<br>• Placebo every other week (loading dose at Weeks 0 and 1)<br>• BG9924 5 mg every other week (loading dose of 3 mg at Weeks 0 and 1)<br>• BG9924 70 mg every other week (loading dose of 40 mg at Weeks 0 and 1)<br>• BG9924 200 mg every other week (loading dose of 120 mg at Weeks 0 and 1)<br>• BG9924 70 mg monthly (alternates with placebo every other visit, loading dose of 40 mg at Weeks 0 and 2)<br>• BG9924 200 mg monthly (alternates with placebo every other visit, loading dose of 120 mg at Weeks 0 and 2)
Visit Schedule:	Subjects may participate in this study for up to 26 weeks. Over the treatment period (Visits 1 to 8/Weeks 0 to 12), subjects will be dosed every other week for 12 weeks, including a loading dose. Subjects are to return to the clinic for a follow-up visit 2 weeks after the last dose (Visit 9/Week 14).<br><br>Subjects who continue in the study until follow-up (Visit 9/Week 14) will be offered the option to enter a safety extension study (under a separate protocol) at that time.<br><br>Subjects who do not enroll into the safety extension study will be followed for an additional 12 weeks (until

	Visit 12/Week 26) under this protocol.
	Study sites may choose to participate in an optional PK sub-study. Approximately 50 consenting subjects will have 6 additional visits following Visits 1 (Visits 1a and 1b), 3 (Visit 3a), and 8 (Visits 8a, 8b, and 8c). Blood samples for FACS analysis will also be taken from subjects participating in this sub-study.
Efficacy Assessments:	American College of Rheumatology (ACR) Core Set measurements (swollen joint count [SJC], tender joint count [TJC], subject's and Investigator's global assessments of disease activity, Health Assessment Questionnaire disability index [HAQ-DI], subject's assessment of pain on visual analog scale [VAS], high sensitivity C-reactive protein [hsCRP], and erythrocyte sedimentation rate [ESR]); Short Form 36 [SF-36] and Functional Assessment of Chronic Illness Therapy [FACIT-F] questionnaires; and Sjogrens syndrome assessments if applicable (subject-reported dryness including VAS [ocular, vaginal, skin, and oral] and Ocular Surface Disease Severity Index [OSDI]).
Study Treatment Concentration Assessments:	A population PK analysis will be performed. An optional intensive PK sub-study is included to allow for detailed PK evaluation in this patient population.
Pharmacodynamic Assessments:	• Quantitative fluorescence-activated cell sorter (FACS) analysis will be performed only in those subjects taking part in the optional intensive PK sub-study. The following analysis will be performed:<br>o Lymphocytosis: CD3+, CD4+, CD8+ CD19+, CD16+,CD56+, and CD14+<br>o Increased unswitched memory B cells: CD19+, IgD+, IgM+, and CD27+<br>• Cytokine panel (including, but not limited to, interleukin [IL]-1β, IL-6, IL-8, tumor necrosis factor-α [TNF-α] and interferon-γ [IFN-γ])
RA Disease Markers:	Assessments include, but are not limited to:<br>• hsCRP

- ESR
- Rheumatoid factor (RF)
- Serum B cell activation factor from the TNF family (BAFF)
- Cartilage oligomeric matrix protein (COMP)
- Anti-cyclic citrullinated peptide-2 (CCP-2) antibody
- Calgranulin
- Matrix metalloproteinase 3 (MMP3)

Safety Assessments:

Physical examinations, vital signs, electrocardiogram (ECG), hematology, lymphocyte and leukocyte subset analysis (CD3+, CD4+, CD8+, CD19+, CD16+, CD56+, and CD14+), blood chemistry, international normalized ratio (INR; for anticoagulant monitoring), prothrombin time (PT), and activated partial thromboplastin time (aPTT), urinalysis, quantitative immunoglobulins (total, IgA, IgG, IgM), tetanus titers, antibodies to BG9924, antinuclear antibodies (ANA), anti-double stranded DNA (anti-dsDNA), and monitoring for infections, adverse events (AEs), and concomitant therapy use.

Other Assessments:

RNA analysis will be performed in order to measure changes in RNA associated with pathogenesis or response to treatment. RNA analysis may include analysis of specific RNA molecules known to be associated with the etiology or pathogenesis of RA, as well as an analysis of all RNA represented in blood cells.

Statistical Analysis:

Efficacy Analyses

Primary Endpoint:

The proportion of subjects with an ACR50 response at Week 14 (Visit 9).

Secondary Endpoints:

- the proportion of subjects with an ACR20 and ACR70 response at Week 14,

- the change from baseline in Quality of Life (HAQ-DI, SF-36, FACIT-F) at Week 14,
- the change from baseline disease activity score (DAS28) at Week 14,
- the proportion of subjects with a categorical DAS28 response (EULAR [European League Against Rheumatism] response) at Week 14, and
- changes from baseline in the individual ACR core set parameters at Week 14.

Exploratory endpoints:

- ACR responses over time (including ACRn)
- Change in Sjogrens assessments in subjects with Sjogrens syndrome: subject-reported dryness, including VAS (ocular, vaginal, skin, and oral) and OSDI.

Endpoints involving proportions responding will be analyzed using a chi-square test, Fisher's exact test, or logistic regression with treatment group as a term in the model, as appropriate. Change from baseline for DAS28, FACIT-F, SF-36, and HAQ-DI scores will be analyzed using an analysis of covariance with treatment group and baseline as terms in the model. ACR responses over time (including ACRn), and changes from baseline in the individual ACR core set parameters, FACIT-F scores, SF-36 scores (the 8 domain scores and the mental and physical health component scores), and Sjogrens assessments will be summarized using descriptive statistics, as applicable.

Safety analyses

The following will be calculated:

- the incidence of all serious adverse events (SAEs),
- the incidence of all AEs,
- the incidence of all infections,
- the proportion of subjects with laboratory assessment values outside the normal range, and
- the proportion of subjects who tested positive for anti-nuclear or tetanus antibodies.

CONFIDENTIAL
The information contained herein may not be used, disclosed, or published without the written consent of Biogen Idec Inc.

	**PK and PD analyses**<br>To assess the PK profile of BG9924, a population PK analysis will be performed.<br>An intensive PK sub-study is included to allow detailed PK evaluation in this patient population.<br>To assess the PD profile of BG9924, summary statistics of parameters from the quantitative FACS analysis, cytokine panel, and RA disease markers will be performed.<br>**Immunogenicity analysis**<br>The proportion of subjects who developed anti-BG9924 antibodies will be summarized.
Primary Analysis:	An analysis on the primary endpoint will be performed when all subjects reach Week 14.

4 STUDY ACTIVITIES 104RA202

Tests and Evaluations	Screening (within 28 days prior to Day 0)	Visit 1 Wk 0 (Day 0)	Visit 1a[a] Day 2	Visit 1b[a] Day 5	Visit 2 Wk 1 (± 2 d)	Visit 3 Wk 2 (± 2 d)	Visit 3a[a] Wk 3 Day 21	Visit 4 Wk 4 (± 3d)	Visit 5 Wk 6 (± 3 d)	Visit 6 Wk 8 (± 3 d)	Visit 7 Wk 10 (± 3 d)	Visit 8 Wk 12 (± 3 d)	Visit 8a[a] Day 86	Visit 8b[a] Day 89	Visit 8c[a] Day 91	Visit 9 Wk 14[b] (± 3 d)
Medical history	X															
Physical examination	X	X														X
Vital signs[1]	X	X			X	X		X	X	X	X	X				X
ACR Core Set measurements[2]	X	X				X		X	X		X					X
QoL (SF-36 / FACIT-F)		X														X
Sjogrens assessments[3]		X														X
hsCRP, ESR[4]	X	X				X		X	X		X					X
RF/ anti-CCP-2	X	X							X							X
RA disease markers[5]/ Cytokine panel		X							X							X
Quantitative FACS analysis[6]		X							X							X
Serum BG9924 PK		X	X	X	X	X	X	X	X	X	X	X	X	X	X	X
Hematology	X	X				X		X	X		X					X
Quantitative Ig		X							X							X
Blood chemistry	X	X				X		X	X		X					X
INR, PT, and aPTT		X														X
Hep C, Hep B test	X															
ANA / anti-dsDNA antibody assays		X														X
Serum anti-BG9924 antibody assay		X							X							X
Tetanus antibody titers		X														X
Serum pregnancy test	X															
Urinalysis	X	X														X
Urine pregnancy test		X														X
PPD (Mantoux test)[7]	X															
Chest X-ray[8]	X															
ECG	X															X
RNA Analysis		X							X							X
Study treatment administration		X			X	X		X	X	X	X	X				
Monitoring of adverse events	<---------- MONITOR AND RECORD THROUGHOUT THE STUDY AS PER INSTRUCTIONS IN SECTION 15.2 ---------->															
Monitoring of concomitant therapy	<---------- MONITOR AND RECORD THROUGHOUT THE STUDY AS PER INSTRUCTIONS IN SECTION 15.2 ---------->															

CONFIDENTIAL
The information contained herein may not be used, disclosed, or published without the written consent of
Biogen Idec Inc.

Tests and Evaluations	Visit 10 Wk 18 (± 3 d)	Visit 11 Wk 22 (± 3 d)	Visit 12 Wk 26[c] (± 3 d)
Medical history			
Physical examination			X
Vital signs[1]	X	X	X
ACR Core Set measurements[2]	X	X	X
QoL (SF-36 / FACIT-F)			X
Sjogrens assessments[3]			X
hsCRP, ESR[4]	X	X	X
RF/ anti-CCP-2			
RA disease markers[5]/ Cytokine panel			
Quantitative FACS analysis[6]			
Serum BG9924 PK		X	X
Hematology			X
Quantitative Ig			X
Blood chemistry			X
INR, PT, and aPTT			X
Hep C, Hep B test			
ANA / anti-dsDNA antibody assays			X[9]
Serum anti-BG9924 antibody assay			X
Tetanus antibody titers			X
Serum pregnancy test			
Urinalysis			X
Urine pregnancy test			
PPD (Mantoux test)[7]			
Chest X-ray[8]			
ECG			
RNA Analysis			
Study treatment administration			
Monitoring of adverse events	MONITOR AND RECORD THROUGHOUT THE STUDY AS PER INSTRUCTIONS IN SECTION 15.2		
Monitoring of concomitant therapy	MONITOR AND RECORD THROUGHOUT THE STUDY AS PER INSTRUCTIONS IN SECTION 15.2		

CONFIDENTIAL
The information contained herein may not be used, disclosed, or published without the written consent of
Biogen Idec Inc.

[1]Vital signs include temperature, heart rate, respiratory rate, and blood pressure. Blood pressure to be performed supine after 5 minutes rest.

[2]Measurements include: swollen and tender joint counts (66/68), subject/Investigator global assessment of disease activity, HAQ-DI, and subject's assessment of pain (VAS)

[3]Assessments include: subject-reported dryness including VAS (ocular, vaginal, skin, and oral) and OSDI. To be conducted in subjects with Sjogrens syndrome only.

[4]ESR to be performed at local laboratory.

[5]RA disease markers include: serum BAFF, COMP, calgranulin, and MMP3.

[6]Only to be taken from subjects participating in the optional PK sub-study.

[7]PPD test to be read 48-72 hours after planting.

[8]Not required if performed within 3 months prior to Day 0 and was without clinically significant abnormality. However, if PPD induration is ≥5 mm, but <10 mm (size of raised bump, not redness), a chest X-ray is required, regardless of when the last X-ray was taken.

[9]Only to be taken from subjects with positive ANA/anti-dsDNA at Week 14.

[a]Visit only applicable to subjects participating in the optional PK sub-study. Subjects will sign a separate informed consent form.

[b]Option to enter safety data extension study. If subject withdraws from the study prior to Visit 9/Week 14, all Week 14 assessments should be completed at the withdrawal visit.

[c]If subject withdraws from the study during the post-treatment period, Week 26 assessments should be completed at the withdrawal visit.

CONFIDENTIAL
The information contained herein may not be used, disclosed, or published without the written consent of
Biogen Idec Inc.

5 INTRODUCTION

BG9924 is a fusion protein consisting of the extracellular domain of the human lymphotoxin beta receptor (LTβR) fused to the human IgG_1 Fc domain. BG9924 is being developed by Biogen Idec Inc. (Biogen Idec) for the treatment of patients with RA. This Phase 2b study was designed to evaluate the efficacy, safety, pharmacokinetics, and pharmacodynamics of BG9924 when given in combination with MTX to subjects with active RA who have had an inadequate response to conventional DMARD therapy.

5.1 Overview of Rheumatoid Arthritis

RA is a chronic rheumatic disease characterized by symmetrical, inflammatory polyarthritis. It occurs in young adults with an average age of onset between 30 and 50 years and affects approximately 2.1 million individuals within the US or roughly 1% of the adult population [1]. Uncontrolled inflammation transforms the synovial lining into an aggressive tissue called pannus that erodes adjacent cartilage and bone. The result is significant morbidity, decreased quality of life, and sometimes, premature death [2, 3].

The goals of therapy are to alleviate joint pain, swelling, and stiffness and to prevent further structural joint damage and maximize the quality of life. Initially, non-steroidal anti-inflammatory drugs (NSAIDs) are prescribed for symptomatic relief. Oral corticosteroids and periodic intra-articular corticosteroids are also beneficial, but are associated with toxicities over time. Disease-modifying anti-rheumatic drugs (DMARDs), the most common being MTX, are given to control the underlying disease, to reduce reliance on corticosteroids, and to provide symptomatic relief, but in many cases provide only partial relief of symptoms.

The introduction of biologic agents for the treatment of RA has been revolutionary. In patients who have either failed MTX or have had an inadequate response to MTX, the addition of an anti-tumor necrosis factor (anti-TNF) agent has resulted in unprecedented levels of efficacy, although few patients attain true clinical remission. Despite the development of TNF inhibitors, a significant proportion of RA patients only achieve a partial response to anti-TNF therapy, lose responsiveness over time, perhaps due to anti-drug antibody effects, or may have a contraindication to these agents such as congestive heart failure. The need for alternative therapies for this segment of the RA population, who continue to experience progressive disability, continues to exist.

5.1.1 Rationale for Use of BG9924 in RA

The primary targets of the immune system in RA are the synovial membrane, cartilage, and diarthrodial joints. During the pathogenesis of RA, T cells, B cells, plasma cells, macrophages, and dendritic cells accumulate in the synovial layer. Within the synovium, a high percentage of the patients have many plasma cells present and altered vasculature. This vasculature includes both activated flat endothelium and a specialized high endothelium like the high endothelial venules (HEV) found in the lymph nodes. In a smaller subset, the infiltrates display a high degree of ectopic cellular organization

similar to that observed in the secondary lymphoid organs, notably, segregation of T and B cells and the formation of germinal centers. The HEV can support the trafficking of a wider range of cell types including naive T and B cells, plasmacytoid dendritic cells, and potentially plasma cell entry, and as such, may drive a local autoimmune response that exacerbates disease[4].

LTα/β-LTβR signaling is critical for the maintenance of HEV, follicular dendritic networks, and T/B cell positioning in the lymph nodes and spleen, and is believed to play a similar role in the ectopic structures that accompany chronic inflammation [5, 6]. The loss of follicular dendritic cell networks results in abortive germinal center reactions. A second ligand called LIGHT also binds to LTBR, and LIGHT is detected in the synovium and may contribute to bone resorption via induction of osteoclasts [7, 8]. LIGHT is generally believed to play a pro-inflammatory role as overexpression in mice induces autoimmune disease [6].

The therapeutic rationale for treatment of RA with BG9924 is based on the general mode of action where the quality of immune responses will be dampened by way of a number of mechanisms, notably via altered trafficking through the lymph nodes and abortive germinal center reactions. This view is supported experimentally by results obtained in rodent models of arthritis using a murine homologue (mLTβR-Ig). Prophylactic treatment with mLTβR-Ig blocked the induction of collagen-induced arthritis (CIA) in mice and adjuvant arthritis in Lewis rats [9]. In mice with established arthritis, mLTβR-Ig treatment blocked disease progression, reducing the severity of arthritic symptoms and joint damage [9]. Although the underlying mechanism of action is not understood, reduced cell trafficking into the draining lymph nodes and a potential reduction in the quality of the pathogenic anticollagen II antibodies were noted [9]. Human RA synoviocytes were shown to respond to LTβR signaling with chemokine release and increased intracellular adhesion molecule (ICAM) expression, indicating a potentially direct proinflammatory consequence of the LT pathway in the joint [10]. The ongoing Phase 2a clinical study (Study 104-RA-201) has also provided encouraging data, showing significant improvements in both tender and swollen joint counts after 4 weeks of dosing. Therefore, BG9924 is felt to be a promising new therapeutic option for the treatment of RA patients.

5.2 Profile of Previous Experience

BG9924 has been administered as a single dose to 130 healthy subjects in 2 double-blind, placebo-controlled, dose-escalation studies (Studies C-1400 and C-1402). Additionally, BG9924 has been administered to 2 subjects with active Crohn's disease, each receiving 4 once weekly doses (Study C-1401), and to over 32 subjects in an ongoing Phase 2a rheumatoid arthritis study (Study 104-RA-201).

Studies C-1400 and C-1402 were conducted in healthy volunteers to examine the effects of single-dose exposure by intravenous (IV), SC, and intramuscular (IM) (Study C-1400 only) administration. Data from these Phase 1 studies demonstrated linear and dose proportional PK. BG9924 was well tolerated with the exception of some transient mild

to moderate flu-like symptoms (fevers, chills, etc.) observed approximately 6 to 12 hours following administration of BG9924. These symptoms were accompanied by transient increases in white blood cell (WBC) counts and CRP levels, and were most commonly seen in the IV dose groups and were milder in nature in both the SC and IM dose groups. In general, the fevers and headaches responded to acetaminophen and resolved within 24 hours.

Study 104-RA-201 is an ongoing randomized, blinded, placebo-controlled, dose-escalating study of BG9924 administered in 4 weekly SC doses to subjects with RA. Subjects remained on their current stable dose of MTX and any other allowed concomitant therapy for RA (e.g., corticosteroids, NSAIDs, and hydroxychloroquine sulfate) throughout the study. To date, 5 dosing cohorts are complete, each consisting of 6 subjects who received BG9924 and 2 subjects who received placebo. The BG9924 dose escalation in these cohorts ranged from 0.05 mg/kg to 3.0 mg/kg. Data collection is ongoing in the 3.0 mg/kg cohort, and enrollment is ongoing in the final cohort (0.01 mg/kg).

The data available to date show BG9924 to be well tolerated with no severe events occurring in any treatment group. The most commonly reported AE was headache. Transient mild to moderate flu-like symptoms have been observed in a small number of subjects after the first dose of BG9924 and did not usually reoccur after subsequent doses.

An exploratory objective of Study 104-RA-201 is to assess the preliminary efficacy of multiple doses of BG9924. The data available to date show BG9924-treated subjects to demonstrate greater improvement than placebo-treated subjects. Overall, BG9924-treated subjects demonstrated significant reductions in both swollen and tender joint counts when compared to placebo, as well as a trend towards an improvement in several other components of the ACR core set measurements.

5.3 Study Rationale

BG9924 binds to the membrane-bound $LT\alpha_1\beta_2$ ($LT\alpha/\beta$ or LT) ligand expressed on activated lymphocytes and natural killer cells and blocks its interaction with LTβR on target tissues (macrophages, dendritic cells, follicular dendritic cells, and stromal cells, including endothelial cells). The LT pathway is important in maintaining the proper cellular positioning within lymphoid tissues. It is believed that BG9924 provides a novel mechanism of action that will target pathways involved in the immunopathogenesis of RA, thereby potentially improving this disease.

This Phase 2b study is designed to evaluate the efficacy and safety of 5 different BG9924 (SC) dose regimens versus placebo over a 12-week treatment period. Eligible subjects are those with active RA who have previously had an inadequate response to treatment with conventional DMARD therapy. This study will assist with dose selection for further planned clinical studies of BG9924.

5.4 Dose Rationale

Dose selection was based upon an interim assessment of pharmacokinetic, efficacy, and safety data from a dose-ranging Phase 2a study performed in RA patients (Study 104-RA-201). In this study, weekly SC doses of BG9924 ranged from 0.05 mg/kg to 3.0 mg/kg for 4 consecutive weeks. PK, efficacy, and safety data were evaluated from Cohorts 1 to 3 (0.05 mg/kg to 0.3 mg/kg) through Day 77, Cohort 4 (1.0 mg/kg) through Day 28, and SAE safety data for Cohorts 1 to 5 (0.05 mg/kg to 3.0 mg/kg weekly for 4 weeks) through their final study visit (Day 77) were also evaluated.

Tender joint counts (TJC) and swollen joint counts (SJC) were monitored, and blood samples were collected at various times after dosing for determination of BG9924 concentration. A correlation was established between the BG9924 AUC and reduction of TJC values. An exposure that correlated to an ED_{50} for TJC reduction at the end of 4 weeks dosing was selected as a target, with additional exposures selected above and below that target to define the study range. A dose of 0.35 mg/kg/week was estimated from the relationship as an ED_{50} for TJC reduction. Pharmacokinetic modeling suggested that equivalent exposure could be achieved with dosing 0.7 mg/kg every other week, or 1.4 mg/kg once a month. The 4-week TJC reduction data suggested that a maximum effect was achieved with a dose of 1.0 mg/kg/week, or 2.0 mg/kg/week every other week, and that a dose of 0.05 mg/kg/week was effective in some subjects. Fixed doses of 5 mg, 70 mg, and 200 mg every other week, and 70 mg and 200 mg once a month, were selected based on these data and historical body weight data in RA patients. A low dose of 5 mg every other week was selected to explore whether activity can be maintained.

A weight restriction of 50 kg has been included within this protocol. Some study subjects given a 200 mg dose could receive an equivalent of 4 mg/kg SC. Although this dose has not been previously tested in humans by the SC route, a 3 mg/kg IV dose has been given to healthy volunteers. Dosing 4 mg/kg SC every other week to steady-state can be expected to produce a mean maximum concentration (C_{max}) of 56.0 μg/mL. Previous administrations of 3 mg/kg IV resulted in mean C_{max} values of 66.0 μg/mL and were considered well tolerated. Additionally, comparisons to preclinical observations have suggested a safety margin of 46 for a 3 mg/kg IV dose. A 200 mg dose should have a safety margin no less than 34, based upon exposure. A 200 mg dose is expected to be well tolerated in this study.

A loading dose strategy will be given to all study subjects for all dosing groups. The loading dose will allow more rapid attainment of steady-state concentrations, and possibly a more rapid response in the relief of symptoms. To achieve steady-state more rapidly, the loading dose regimen will consist of 2 doses, together equal to approximately 1.2 times than the steady-state doses, and will be split, such that one-half of the dose is given on the first visit and the second half on either the second or third visit. For every other week regimens, one-half of the loading dose will be given on Week 0 (Visit 1) and the second half on Week 1 (Visit 2). For monthly regimens, one-half of the loading dose will be given on Week 0 (Visit 1) and the second half on Week 2 (Visit 3).

CONFIDENTIAL
The information contained herein may not be used, disclosed, or published without the written consent of Biogen Idec Inc.

6 OBJECTIVES

6.1 Primary Objective

The primary objective of the study is to evaluate the efficacy of 5 dose regimens of BG9924 when administered in combination with MTX to subjects with active RA who have had an inadequate response to conventional DMARD therapy. The primary efficacy endpoint is the proportion of subjects with ACR50 at Week 14. To achieve ACR50 response, a 50% improvement compared to baseline is required for both swollen and tender joint counts, as well as 3 out of 5 additional parameters: subject's global assessment of disease activity, Investigator's global assessment of disease activity, subject's assessment of pain, HAQ-DI, and hsCRP or ESR.

6.2 Secondary Objectives

Secondary objectives of this study are as follows:

- To assess the safety and tolerability of these 5 dose regimens of BG9924 in this patient population.
- To assess the PK and PD profile of these 5 dose regimens of BG9924 in this patient population.

7 STUDY DESIGN

7.1 Study Overview

This is a randomized, double-blind, placebo-controlled, multicenter, dose-finding study of BG9924 in subjects with active RA who have had an inadequate response to conventional DMARD therapy. Approximately 380 subjects will be enrolled at approximately 60 sites. Subjects may participate in this study for up to 26 weeks. Over the treatment period, subjects will receive BG9924 (1 of 5 dosing regimens) or placebo by SC injection, every other week for 12 weeks (including a loading dose, with the first half given at Week 0 and second half at Weeks 1 or 2), with a follow-up visit 2 weeks after the last dose (Visit 9/Week 14). Subjects who continue in the study until Week 14 will be offered the option to enter a safety extension study (under a separate protocol). Subjects who do not enroll in the extension study will be followed for safety assessments for an additional 12 weeks (until Visit 12/ Week 26) under this protocol.

Study sites may choose to participate in an optional PK sub-study. Approximately 50 consenting subjects will have 6 additional visits for blood draws following Visits 1 (Visits 1a and 1b), 3 (Visit 3a), and 8 (Visits 8a, 8b, and 8c). Blood samples for FACS analysis will also be taken from subjects participating in this sub-study.

7.2 Overall Study Duration and Follow-Up

The study period will consist of Screening, treatment period (Visits 1 to 8/Weeks 0 to 12), and post-treatment period (Visit 9/Week 14 only, or Visit 9/Week 14 to Visit 12/Week 26, depending on whether subjects choose to enroll in the safety extension study).

Screening

Subject eligibility for the study will be determined within 28 days prior to study enrollment.

Treatment Period

Eligible subjects will report to the study site according to the table of study activities (Section 4) and the Schedule of Events (Section 14) during the treatment period. Over this treatment period (Visits 1 to 8/Weeks 0 to 12), subjects will be dosed every other week for 12 weeks, including a loading dose, with the first half given at Week 0 and second half at Weeks 1 or 2. Subjects participating in the PK sub-study will have additional visits for blood draws.

Post-Treatment Period

Subjects are to return to the study site for a follow-up visit at Week 14 (Visit 9, 2 weeks after the last dose) for primary endpoint evaluations. At this visit, subjects will be offered the option to enter a safety extension study, under a separate protocol. Subjects who do not enroll into the safety extension study will be followed for an additional 12 weeks (until Visit 12/Week 26) under this protocol. Therefore, the final visit will be either Visit 9/Week 14 or Visit 12/Week 26, depending on whether subjects choose to enroll in the safety extension study.

End of Study

The end of the study is the date on which the last subject has his or her last visit. The last visit will be either Visit 9/Week 14 (primary endpoint evaluation and option to enter a safety extension study) or Visit 12/Week 26.

7.3 Study Stopping Rules

Biogen Idec, in conjunction with the Data Monitoring Committee (DMC), may terminate this study, after informing Investigators, at any time. Investigators will be notified by Biogen Idec or its designee if the study is placed on hold, completed, or closed.

CONFIDENTIAL
The information contained herein may not be used, disclosed, or published without the written consent of Biogen Idec Inc.

8 STUDY POPULATION

8.1 Inclusion Criteria

Unless otherwise specified, to be eligible to participate in this study, candidates must meet the following eligibility criteria at Day 0 (Visit 1/Week 0):

1. Must give written informed consent and any authorizations required by local law (e.g., Protected Health Information [PHI]).
2. Aged 18 to 75 years old, inclusive, at the time of informed consent.
3. Must have a diagnosis of adult onset RA according to the 1987 Revised American Rheumatism Association Criteria for the Classification of Rheumatoid Arthritis (Functional Class I – III) (Appendix A) for at least 6 months prior to Day 0.
4. Must have been treated with, and be tolerating, MTX (≥10 mg/week to ≤ 25 mg/week) for at least 3 months immediately prior to Day 0. The dose of MTX must be stable for at least 4 weeks prior to Day 0.
5. Must have had an inadequate response to at least 1 conventional DMARD therapy (i.e., MTX, leflunomide, sulfasalazine, etc.) due to inadequate efficacy or toxicity.
6. Must have a Swollen Joint Count (SJC) ≥8 and a Tender Joint Count (TJC) ≥8 (66/68 joint count at Screening).
7. Must have elevated hsCRP ≥0.8 mg/dL at Screening.
8. Must be willing to receive oral folate (≥5 mg/week) for the duration of the study.

8.2 Exclusion Criteria

Unless otherwise specified, candidates will be excluded from study entry if any of the following exclusion criteria exist at Day 0 (Visit 1/Week 0):

MEDICAL HISTORY

1. Subjects with a body weight <50 kg.
2. Subjects with a history of a malignancy or carcinoma *in situ* (subjects with a history of excised or treated basal cell carcinoma are eligible to participate in this study).
3. Subjects with a mole or lesion currently undiagnosed, but suspicious for malignancy. Any suspicious skin lesion should be evaluated and excised by a dermatologist prior to inclusion in the study.
4. History of clinically significant (as determined by the Investigator) cardiac, allergic, endocrinologic, pulmonary, neurologic, psychiatric, hepatic, renal or hematologic insufficiency, **or** any major disease that could affect any of the efficacy assessments, in particular, joint pain and swelling (e.g., Parkinsons disease, cerebral palsy, diabetic neuropathy).

CONFIDENTIAL
The information contained herein may not be used, disclosed, or published without the written consent of Biogen Idec Inc.

5. Subjects with rheumatic autoimmune disease other than RA, or significant systemic involvement secondary to RA (including, but not limited to vasculitis, pulmonary fibrosis, or Felty's syndrome). Secondary Sjogren's syndrome or secondary limited cutaneous vasculitis within RA is permitted.
6. Serious local infection (e.g., cellulitis, abscess) or systemic infection (e.g., pneumonia, septicemia) within 3 months prior to Day 0 (Visit 1/Week 0).
7. History of *recurrent* infections requiring oral or parenteral anti-infective drug treatment.
8. Primary or secondary immunodeficiency (history of or currently active), including known history of HIV infection.
9. History of tuberculosis or positive purified protein derivative (PPD; positive Mantoux test defined as ≥10 mm of induration [size of raised lump, not redness], or equivalent positive tuberculosis (TB) test result as per country clinical standards) during the screening period. When the PPD induration is ≥5 mm, but <10 mm the subject is eligible for the study if they have had a negative chest x-ray during the screening period. There must be no other clinical evidence of TB on physical examination of the subject.
10. Fever (body temperature >38°C) or symptomatic viral infection or bacterial infection within 2 weeks prior to Day 0.
11. Receipt of live vaccine within 4 weeks prior to Day 0.
12. Clinically significant chest x-ray abnormality at Screening (Note: chest x-ray is not required if one was performed within 3 months of Day 0 and was without clinical abnormality).

<u>LABORATORY TESTS</u>

13. Subjects with any laboratory test result at Screening considered clinically significant (as determined by the Investigator) or
    - Aspartate aminotransferase (AST) or alanine aminotransferase (ALT) >1.5 times than the upper limit of normal (ULN) established by the central laboratory.
    - Platelet count <150,000/µL
    - Hemoglobin <8.5 g/dL
    - Neutrophils <1.5 x 10^3/µL.
14. Positive for hepatitis C antibody or hepatitis B [HBsAg, HbcAb, or HBsAb] at Screening.

<u>TREATMENT HISTORY</u>

15. Previous treatment with anti-TNF therapy or any other non-TNF inhibitor biologic or prosorba column for the treatment of RA.

16. If subjects have previously received cell-depleting therapies, relevant cell counts must have returned to within normal range.
17. Treatment with another investigational drug within the 3 months prior to Day 0 or within 5 half-lives of the agent, whichever is longer.
18. Previous exposure to BG9924.
19. Subjects treated with the following concomitant medications:
    - Any oral steroid exceeding 10 mg/day of prednisone or equivalent and/or not administered at a stable dose for at least 4 weeks prior to Day 0.
    - Leflunomide ≤8 weeks prior to Day 0.
    - Cyclosporin A ≤6 months prior to Day 0.
    - Azathioprine or 6-MP ≤28 days prior to Day 0.
    - Hydroxychloroquine sulfate or sulfasalazine (or equivalents), or any other allowed concomitant DMARDs, at doses greater than the recommended therapeutic dose and/or not administered at a stable dose for 4 weeks prior to Day 0.
    - Any NSAIDS not at a stable dose for least 2 weeks prior to Day 0.
    - Intra-articular corticosteroid injections given within 4 weeks prior to Day 0.
20. Subjects who underwent any surgical procedure, including bone/joint surgery/synovectomy (including joint fusion or replacement), within 12 weeks prior to Day 0 or who are planning an unapproved (by Biogen Idec) procedure within 24 weeks of Day 0.

<u>MISCELLANEOUS</u>

21. Nursing mothers, pregnant women, or women who are planning to become pregnant while in the study.
22. Male and female subjects of childbearing potential not willing to practice effective birth control for the duration of the study. Female subjects must be: (1) postmenopausal for at least 12 months, (2) surgically sterile, or (3) willing to use 2 documented forms of birth control (e.g., barrier and spermicide, intrauterine device and barrier or spermicide, or birth control pill and barrier or spermicide).
23. Blood donation (1 unit or more) within 1 month prior to Screening.
24. History of drug or alcohol abuse (as determined by the Investigator) within the 1 year prior to Day 0.
25. Current enrollment in any other investigational drug study.
26. Previous participation in this study.

27. Subjects who are expected to be unavailable for the duration of the trial, likely to be noncompliant with the protocol, or who are felt to be unsuitable by the Investigator/Sponsor for any other reason.

8.3 Screening Log

Participating study sites are required to document all screened candidates initially considered for inclusion in this study. If a subject is excluded from the study, the reasons for exclusion will be documented in the subject's source documents and on the screening log.

9 ENROLLMENT AND RANDOMIZATION PROCEDURES

9.1 Enrollment Procedures

Subjects will be enrolled at Day 0 (Visit 1/Week 0), after all baseline assessments have been completed. No subject may begin treatment prior to assignment of a unique subject identification number. Any subject identification numbers that are assigned will not be reused even if the subject does not receive treatment.

9.2 Randomization Procedures

Subjects will be randomized after the Investigator has verified that they are eligible per criteria in Section 8.1 and Section 8.2. Randomization will be stratified by screening anti-CCP-2 status. Subjects will be randomized to receive placebo or BG9924 (1 of 5 dosing regimens) in a 2:2:2:2:1:1 ratio. Subjects withdrawn from the study may possibly be replaced.

Randomization will take place across all study sites using a centralized Interactive Voice Response System (IVRS; see Section 19.1.1). At randomization, the IVRS will assign a unique 6-digit subject identification number to each subject (the first segment of the number represents the study site and the second segment of the number represents the subject at that study site). The subject's identification number will be used on all of that subject's Case Report Forms (CRFs), test results, etc.

9.3 Blinding Procedures

This is a randomized, multicenter, double-blinded, placebo-controlled study. The randomization will be computer generated and will be prepared by Biogen Idec by an unblinded statistician not involved with the operations of the study, prior to the start of the study. All study staff will be blinded to the subject treatment assignments, with the exception of the Pharmacist or designee, who is responsible for preparing the study treatment (BG9924 or placebo), and the Pharmacy Monitor. DMC members and the statistician providing data to the DMC will also be unblinded. To maintain the study blind, it is imperative that subject treatment assignments are not shared with the subjects, their families, or any member of the study team at the study site, Biogen Idec, [redacted]

A matching placebo (0.9% Sodium Chloride for Injection) for BG9924 will be used in this study. The placebo is to be provided by the Investigational Sites. The precautions below are to be taken to ensure that blinding is adequately maintained throughout the study.

Dose-related effects of BG9924 on lymphocytes were noted in previous studies, so a potential exists for the Investigator to identify whether a subject is receiving BG9924 or placebo. To avoid compromising the blind, the sites will not receive the results of the lymphocyte counts from any visit after Screening, but will receive all other laboratory results.

To prevent potential blind breaks due to observed efficacy or laboratory changes, a “dual assessor” approach will be used to evaluate efficacy and safety. The Efficacy Assessor will only have access to the clinical efficacy data. The Safety Assessor will have access to both safety and efficacy data. This Investigator will make all treatment decisions based on the subject’s clinical response and laboratory parameters.

10 STUDY TREATMENT DESCRIPTION AND ALLOCATION

Study treatment must be stored in a secure location. Accountability for study treatment is the responsibility of the Investigator. More details concerning this responsibility are included in Section 10.4.

Study treatment must only be dispensed by a Pharmacist or medically qualified staff. Study treatment is to be dispensed only to subjects enrolled in this study. Once study treatment is prepared for a subject, it can only be administered to that subject. Study treatment vials are for *one-time use only*; any study treatment remaining in the vial should not be used for another subject.

Study site staff should refer to the Directions for Handling and Administration (DHA) located in the Pharmacy Manual and the Study Reference Guide for specific instructions on the handling and administration of the study treatment. Any changes to the DHA will be communicated to the study sites by updates to the DHA document and would supersede instructions provided in this protocol and all other references (e.g., Investigator Brochure).

10.1 BG9924

[redacted]

10.2 Comparator

The placebo (control agent) to be used in this study will be sterile normal saline (0.9% Sodium Chloride for Injection). Sterile normal saline will be supplied by the investigational site. The manufacturer's directions for material storage and handling are to be followed, as are standard clinical practices for ensuring sterility of the material.

10.3 Additional Protocol-Designated Products

Methotrexate

Subjects must continue on MTX (≥10 mg/week to ≤ 25 mg/week) through Week 14 of the study (primary endpoint analysis), as prescribed by their rheumatologist or general care physician. The MTX treatment regimen should remain stable and the route of administration should not be changed through Week 14 (and through Week 26 if possible). The dose may be decreased if required due to toxicity. The dose should not be increased prior to Week 14. [redacted]

Oral Folate

All subjects must agree to take oral folate (≥5 mg/week) for the duration of the study (as per the inclusion criteria) to counter the inhibitory effect of MTX on the folic acid metabolic pathway.

10.4 Study Treatment Accountability

The study site must maintain accurate records demonstrating dates and amount of study treatment received, to whom dispensed (subject-by-subject accounting), and accounts of any study treatment accidentally or deliberately destroyed.

Unless otherwise notified, all vials both used and unused, must be saved for study treatment accountability. At the end of the study, reconciliation must be made between the amount of study treatment supplied, dispensed, and subsequently returned to Biogen Idec. A written explanation will be provided for any discrepancies.

After all investigational drug is accounted for, the Investigator must return all unused vials of study treatment as instructed by Biogen Idec, unless permission is granted by Biogen Idec to destroy drug on site.

If any study treatment supplies are to be destroyed at the site, the institution/Principal Investigator(s) must obtain prior approval by Biogen Idec. After such destruction, the institution/Principal Investigator(s) must notify Biogen Idec, in writing, of the method of destruction, the date of destruction, and the location of destruction.

CONFIDENTIAL
The information contained herein may not be used, disclosed, or published without the written consent of Biogen Idec Inc.

11 TREATMENT

11.1 Treatment Schedule

Subjects will receive BG9924 or placebo via SC injection by a healthcare provider at the study site every other week for 12 weeks. All subjects will receive a loading dose as described below. Subjects will be randomized to one of the following dose groups:

- Placebo every other week (loading dose at Weeks 0 and 1)
- BG9924 5 mg every other week (loading dose of 3 mg at Weeks 0 and 1)
- BG9924 70 mg every other week (loading dose of 40 mg at Weeks 0 and 1)
- BG9924 200 mg every other week (loading dose of 120 mg at Weeks 0 and 1)
- BG9924 70 mg monthly (alternates with placebo every other visit, loading dose of 40 mg at Weeks 0 and 2)
- BG9924 200 mg monthly (alternates with placebo every other visit, loading dose of 120 mg at Weeks 0 and 2)

11.2 Removal of Subjects from Treatment and/or Study

11.2.1 Discontinuation of Treatment

Unless otherwise indicated, a subject *must* permanently discontinue BG9924 for any of the following reasons:

- Occurrence of severe (in the opinion of the Investigator) local injection site reaction or hypersensitivity reaction.
- Occurrence of any clinically significant (in the opinion of the Investigator) opportunistic infection or other serious infection.
- Occurrence of any non-cutaneous malignancies or melanoma.
- The subject becomes pregnant. Treatment must be discontinued *immediately*. Report the pregnancy according to the instructions in Section 15.5.2.
- The subject desires to discontinue study treatment under this protocol.
- The subject experiences a medical emergency that necessitates permanent discontinuation of treatment.
- The subject experiences a medical emergency that necessitates unblinding of the subject's treatment assignment.
- The Investigator determines discontinuing the study treatment is in the best interest of the subject.

Subjects who prematurely discontinue treatment will be permanently withdrawn from treatment, and every effort must be made to encourage them to complete all follow-up

CONFIDENTIAL
The information contained herein may not be used, disclosed, or published without the written consent of Biogen Idec Inc.

visits from Visit 9/Week 14 to Visit 12/Week 26. Subjects who prematurely discontinue may possibly be replaced in the accrual scheme. The reason(s) for discontinuation of the study treatment must be recorded in the subject's CRF.

11.2.2 Withdrawal of Subjects From the Study

Subjects must be withdrawn from the study for any one of the following reasons:

- The subject desires to discontinue participation in this study.
- The subject is unwilling or unable to comply with the protocol.
- At the discretion of the Investigator or Sponsor

Subjects who prematurely withdraw from the study during the treatment period (prior to Visit 9/Week 14) should be encouraged to complete the tests and evaluations for Visit 9/Week 14 (primary endpoint evaluation), if possible. Subjects who prematurely withdraw from the study during the post-treatment period should be encouraged to complete the tests and evaluations for Visit 12/Week 26. Subjects who prematurely withdraw may be replaced in the accrual scheme. The reasons for the subject's withdrawal from the study must be recorded in the subject's CRF.

11.3 Treatment Compliance

Compliance with treatment dosing is to be monitored and recorded by site staff. Missed doses cannot be made up.

11.4 Concomitant Therapy and Procedures

A concomitant therapy is any drug or substance administered from Screening until the end of the study. This includes any preventative vaccines (e.g., flu vaccines) that the subject may receive during the course of this study. The dose of allowed concomitant therapy may be decreased if required due to toxicity. The dose should not be increased.

A concomitant procedure is any therapeutic intervention (e.g., surgery/biopsy, physical therapy) or diagnostic assessment (e.g., blood gas measurement, bacterial cultures) performed from Screening until the end of the study.

11.4.1 Allowed Concomitant DMARD Therapy

All subjects must be receiving MTX in the dose range ≥10 mg/week to ≤ 25 mg/week at Day 0, and the dose must have been stable for at least 4 weeks prior to Day 0. The dose of MTX must remain stable through to Week 14 (primary endpoint analysis), and be recorded in the CRF according to the CRF instructions.

It is possible that certain AEs that are commonly associated with MTX treatment may occur. Therefore, in order to minimize MTX toxicity, all subjects will receive folate or equivalent at a stable dose of ≥5 mg/week. This can either be as single dose given

weekly or as a divided weekly dose. It is the Investigator's decision as to which dosing regimen is used.

Concomitant use of corticosteroids for the treatment of RA is permitted if the dose is stable for at least 4 weeks prior to Day 0 and remains consistent through to Week 14. The dose must not exceed 10 mg/kg of prednisone or equivalent. No corticosteroid administration by the intra-articular route is permitted during the study.

Concomitant use of NSAIDs is permitted if the dose is stable for at least 2 weeks prior to Day 0 and remains consistent through to Week 14.

Subjects are to remain on their current stable dose of other DMARDs (hydroxychloroquine sulfate or sulfasalazine [or equivalents], or any other DMARDs unless explicitly disallowed in Section 11.4.2) through to Week 14. Doses must have been stable for 4 weeks prior to Day 0.

11.4.2 Disallowed Concomitant Therapy

At no time during study participation may subjects receive treatment with:

- anti-TNF therapy (e.g., etanercept, infliximab, or adalimumab) or any other biologics (e.g., abatacept or rituximab),
- prosorba column,
- cyclosporin A,
- leflunomide,
- azathioprine,
- 6-MP,
- any other investigational drug or approved therapy for investigational use, or
- live or live attenuated vaccines (subjects may receive flu vaccines)

Subjects should be instructed not to start taking any new medications or therapies, including non-prescribed drugs, unless they have received permission from the Investigator.

From the start of study treatment through to Week 14, the following will be considered a rescue intervention and the subject will be considered a treatment failure:

- If the dose of any protocol-allowed concomitant DMARD therapy (including MTX) needs to be increased or
- If the administration of any new protocol-allowed or disallowed DMARD therapy is needed for the medical management of the subject.

However, it is important to note that the dose of protocol-allowed DMARD therapy can be decreased when clinically indicated for managing associated toxicity, and the subject will not be considered a treatment failure.

Any medication and any non-drug therapy or procedure used from signing of the Informed Consent Form (ICF) until the completion of the study must be recorded in the subject's CRF, according to the instructions for CRF completion. AEs related to administration of these therapies or procedures must be documented on the appropriate CRF.

11.5 Continuation of Treatment

Subjects who continue in the study until Visit 9/Week 14 will be offered the option to enter a safety extension study (under a separate protocol) at that time. Subjects who do not enter the safety extension study are encouraged to complete all post-treatment visits to Visit 12/Week 26.

12 EFFICACY, BG9924 CONCENTRATION, AND PHARMACODYNAMIC ASSESSMENTS

12.1 Clinical Efficacy Assessments

A written, signed ICF must be obtained prior to performing any tests or evaluations under this protocol.

The following clinical tests/assessments will be performed to assess the efficacy of BG9924:

- ACR Core Set measurements (SJC, TJC, subject's and Investigator's global assessments, HAQ-DI, subject's assessment of pain on VAS, hsCRP, and ESR [Appendix B, C])
- Quality of Life (QoL) questionnaires (SF-36, FACIT-F; Appendices D, E)
- Sjogrens Assessments in subjects who have Sjogrens syndrome (subject-reported dryness including VAS [ocular, vaginal, skin, and oral] and OSDI [Appendix F])

Every effort should be made to ensure the same physician completes the ACR Core Set measurements and other efficacy assessments at each visit for any one subject.

To prevent potential unblinding due to observed efficacy or laboratory changes, a "dual assessor" approach will be used to evaluate efficacy and safety.

The Efficacy Assessor should be a rheumatologist or skilled arthritis assessor. The Efficacy Assessor will only have access to the clinical efficacy data.

The Safety Assessor should be a rheumatologist and will have access to both safety and efficacy data. The Safety Assessor will make all treatment decisions based on the subject's clinical response and laboratory parameters (see below).

Every effort should be made to have the assessments completed by the subject and the Efficacy Assessor ***before*** *those of the Safety Assessor. Every effort should be made to conduct the assessments at the same time of day, in the following order:*

- Subject-based assessments: Pain, global assessment, HAQ-DI, SF-36, and FACIT-F (and Sjogrens assessments, if applicable)
- Efficacy Assessor: Joint counts, Investigator's global assessment, and Sjogrens assessments, if applicable
- Safety Assessor: Safety assessments (AEs, concomitant therapy, and review of laboratory data)

The following study assessments will be performed according to the schedules in Sections 4 and 14.2.

- Subject's assessment of pain: The subject's assessment of the subject's current level of pain on a 100 mm horizontal VAS. The left-hand extreme of the line should be described as "no pain" and the right-hand extreme as "unbearable pain."
- Subject's global assessment of disease activity: The subject's overall assessment of their current disease activity on a 100 mm horizontal VAS. The left-hand extreme of the line should be described as "no disease activity" (symptom-free and no arthritis symptoms) and the right-hand extreme as "maximum disease activity" (maximum arthritis disease activity).
- HAQ-DI: The Stanford Health Assessment Questionnaire disability index is a subject-completed questionnaire specific for RA (Appendix C). It consists of 20 questions referring to 8 component sets: dressing/grooming, arising, eating, walking, hygiene, reach, grip, and activities. The questionnaire will be provided in validated translation into the local languages at the participating sites and will be scored based on the instructions from the Stanford University Medical Center.
- SF-36 (Version 2): The Short Form 36 questionnaire is used to compare health outcomes among groups of patients, to screen individuals for a variety of health concerns, and to monitor health outcomes over time (Appendix D). It is a scientifically validated questionnaire that captures patient reported outcomes and will be provided in validated translations into the local languages at the participating sites and will be scored based on the instructions from Quality Metric™.
- FACIT-F: The Functional Assessment of Chronic Illness Therapy- Fatigue (FACIT-F), Version 4, will be used (see Appendix E). This is a 13-item questionnaire, in which patients are requested to score each question on a 0-4 scale. The assessment was originally developed for chronic illnesses and is now validated for patients with RA.

CONFIDENTIAL
The information contained herein may not be used, disclosed, or published without the written consent of Biogen Idec Inc.

- Swollen/tender joint count: An assessment of 66 joints for swelling and 68 joints for tenderness will be made. Joints will be assessed and classified as swollen/not swollen and tender/not tender by pressure and joint manipulation on physical examination. Joint prosthesis, arthrodesis, or fused joints will not be assessed for swelling or tenderness. The joints to be assessed for swelling and tenderness are given in Appendix B. This should be completed by the Efficacy Assessor.
- Investigator's global assessment of disease activity: The Investigator's assessment of the subject's current disease activity on a 100 mm horizontal VAS. The left-hand extreme of the line should be described as "no disease activity" (symptom-free and no arthritis symptoms) and the right-hand extreme as "maximum disease activity" (maximum arthritis disease activity). This should be completed by the Efficacy Assessor.
- hsCRP or ESR: The hsCRP samples will be analyzed by a central laboratory. ESR will be analyzed by a local laboratory. Either hsCRP or ESR will be used for determination of ACR response rate.

12.2 RA Disease Marker Assessments

The following laboratory tests/assessments to assess the efficacy and PD of BG9924 will be performed by the central laboratory.

- hsCRP (high sensitivity C-reactive protein)
- RF (rheumatoid factor)
- Anti-CCP-2 (cyclic citrullinated peptide-2) antibody

The following assessments will be performed by Biogen Idec or a specialized laboratory selected by Biogen Idec and include, but are not limited to:

- Serum BAFF (B cell activation factor from the TNF family)
- COMP (cartilage oligomeric matrix protein)
- Calgranulin
- MMP3 (matrix metalloproteinase 3)

Blood samples for these assessments may be stored for up to 10 years prior to analysis.

ESR (erythrocyte sedimentation rate) will be performed at a local laboratory.

12.3 BG9924 Concentration Assessments

BG9924 concentrations will be measured to support the PK determinations in this study.

12.4 Pharmacodynamic Assessments

The following tests to assess the PD properties of BG9924 will be performed:

- Quantitative FACS analysis (only in those subjects taking part in the optional intensive PK sub-study):
    - Lymphocytosis: CD3+, CD4+, CD8+, CD19+, CD16+, CD56+, and CD14+
    - Increased unswitched memory B cells: CD19+, IgD+, IgM+, and CD27+

The FACS analysis will be performed by a specialized laboratory selected by Biogen Idec.

- Cytokine panel (including but not limited to IL-1β, IL-6, IL-8, TNF-α, and IFN-γ)

The cytokine analysis will be performed by Biogen Idec or a specialized laboratory selected by Biogen Idec. Blood samples may be stored for up to 10 years prior to analysis.

12.5 Other Assessments

Additional blood samples for RNA analysis will be taken at the timepoints outlined in Section 4. These samples will be used to identify dynamic biomarkers that will help us better understand the pathogenesis and response to treatment of patients with RA.

The blood samples taken for RNA analysis will be used only for measuring changes in RNA associated with pathogenesis or response to treatment. RNA analysis may include analysis of specific RNA molecules known to be associated with the etiology or pathogenesis of RA, as well as an analysis of all RNA represented in blood cells.

These samples will be used only for research in RA and will not be used for genotyping or DNA analysis. DNA will not be extracted from this sample and will therefore not be used to study disease susceptibility.

Sampling for the assessments described will be approved at the discretion of each site's Ethics Committee. If a site's Ethics Committee does not approve the sampling for the exploratory assessments, this section will not be applicable to that site.

Blood samples may be stored for up to 10 years prior to analysis.

CONFIDENTIAL
The information contained herein may not be used, disclosed, or published without the written consent of Biogen Idec Inc.

13 SAFETY ASSESSMENTS

13.1 Clinical Safety Assessments

The following clinical assessments will be performed to assess the safety profile of BG9924:

- Physical examination
- Vital signs
- ECG
- Monitoring of AEs
- Monitoring of concomitant therapy

13.2 Laboratory Safety Assessments

The following laboratory tests will be performed by the central laboratory to assess the safety profile of BG9924:

- International normalized ratio (for anticoagulant monitoring [INR]), prothrombin time (PT), and activated partial thromboplastin time (aPTT)
- Urinalysis (protein, glucose, ketones, occult blood, and WBCs by dipstick and microscopic examination, if indicated)
- Blood chemistry (AST, ALT, alkaline phosphatase, total protein, albumin, cholesterol, total bilirubin, blood urea nitrogen, uric acid, creatinine, lactate dehydrogenase [LDH], potassium, sodium, chloride, calcium, phosphorous, bicarbonate, and glucose)
- Hematology (hemoglobin, hematocrit, red blood cell [RBC] count, WBC count [absolute and differential counts], and platelet count)
- ANA and anti-dsDNA antibody assays
- Quantitative Ig (total, IgA, IgG, and IgM)
- Serum anti-BG9924 antibody assay
- Leukocyte subset analysis: CD3+, CD4+, CD8+, CD19+, CD16+, CD56+, and CD14+

To preserve the study blind, lymphocyte counts will not be sent to the investigational sites.

14 SCHEDULE OF EVENTS

A written, signed ICF and all authorizations required by local law (e.g., PHI in North America) must be obtained prior to performing any tests or assessments under this protocol.

CONFIDENTIAL
The information contained herein may not be used, disclosed, or published without the written consent of Biogen Idec Inc.

14.1 Subject Management

Immunization with a live vaccine is specifically excluded within 4 weeks prior to Day 0 and throughout the study.

Subjects must avoid donating blood during the study.

Subjects should be observed for up to 1 hour after each administration of study treatment.

Subjects must maintain their stable MTX treatment regimen in the dose range ≥10 mg/week to ≤25 mg/week throughout the study. Subjects must also maintain their other allowed concomitant medication at a stable dose, if applicable (see Section 11.4.1).

Male subjects and female subjects of childbearing potential must practice effective contraception during the study and continue contraception for 3 months after their last dose of study treatment. Female subjects are exempt from contraception requirements if they are post-menopausal for at least 1 year before enrollment or are surgically sterile, i.e., no uterus or no ovaries. Males who have undergone vasectomy or females who have undergone tubal ligation are not exempt from contraception requirements during the conduct of the study.

Due to the way it affects the immune system, it is possible that BG9924 might increase a subject’s chance of infection during the study, including infections with viruses or tuberculosis. Non-clinical data has shown that animals infected with tuberculosis or other infectious agents and given a drug similar to BG9924 had an impaired ability to respond to infection. Therefore, subjects may have an increased risk of infection when coming into contact with people who are infected. Prior to foreign travel during the study, subjects should consult the Investigator for appropriate preventative treatments, as well as avoid travel to countries or regions where unusual infections, such as tuberculosis, are known to be a significant risk.

14.2 Tests and Assessments

Due to the “dual assessor” approach to evaluate efficacy and safety, the efficacy assessments are to be performed prior to the safety assessments as per Section 12.1, whenever possible.

The sequence of assessments at each visit will be standardized as follows at visits as required by the schedule of tests and evaluations:

- Subject-reported assessments (subject’s global assessment of disease activity [VAS]; pain [VAS]; HAQ-DI; FACIT-F; SF-36; Sjogrens assessments, if applicable)
- Efficacy assessments (tender and swollen joint counts; Investigator’s global assessment of disease activity [VAS])
- Safety assessments

CONFIDENTIAL
The information contained herein may not be used, disclosed, or published without the written consent of Biogen Idec Inc.

For the subject-reported assessments, data may only be recorded on the source documents on behalf of the subject by the study nurse/Investigator if the subject has difficulty writing during the visit or is unable to read. This must be documented clearly in the subject notes.

All assessments at each visit should be conducted prior to study treatment administration.

14.2.1 Screening Visit (Within 28 Days Prior to Day 0)

The following tests and evaluations will be performed at the Screening Visit:

- Complete medical history
- Physical examination
- Vital signs: temperature, heart rate, respiratory rate, and supine blood pressure (after 5 minutes' rest)
- ACR core set measurements (see Section 12.1 for list of measurements)
- Blood samples collected for:
    - hsCRP and ESR (ESR to be performed at local laboratory)
    - RF and anti-CCP-2 antibody
    - Hematology (see Section 13.2 for list of variables)
    - Blood chemistry (see Section 13.2 for list of variables)
    - HCV, HBsAg, HBsAb, and HBcAb
    - Serum pregnancy test
- Urinalysis (see Section 13.2 for list of variables)
- PPD (Subject must return to clinic 48 – 72 hours after PPD planting to have results of Mantoux test read.)
- Chest X-ray (not required if chest X-ray was performed within 3 months prior to Day 0 and was without clinically significant abnormality). However, if PPD induration is ≥ 5 mm, but <10 mm (size of raised bump, not redness), a chest X-ray is required during the screening period, regardless of when the last X-ray was taken.
- ECG
- Monitoring of SAEs (see Section 15.2)
- Monitoring of concomitant therapy

14.2.2 Visit 1 (Week 0, Day 0)

- Physical examination

CONFIDENTIAL
The information contained herein may not be used, disclosed, or published without the written consent of Biogen Idec Inc.

- Vital signs: temperature, heart rate, respiratory rate, and supine blood pressure (after 5 minutes' rest)
- ACR core set measurements (see Section 12.1 for list of measurements)
- QoL (SF-36, FACIT-F)
- Sjogrens Assessments (see Section 12.1 for assessments), if applicable
- Blood samples collected for:
    - hsCRP and ESR (ESR to be performed at local laboratory)
    - RF and anti-CCP-2 antibody
    - RA Disease Markers (serum BAFF, COMP, calgranulin, and MMP3)
    - Quantitative FACS analysis, only in subjects participating in the optional PK sub-study (see Section 12.4 for list of variables)
    - Cytokine panel (see Section 12.4 for list of variables)
    - Serum BG9924 PK concentration
    - Hematology (see Section 13.2 for list of variables)
    - Quantitative Ig (IgA, IgG, and IgM)
    - Blood chemistry (see Section 13.2 for list of variables)
    - INR, PT, and aPTT
    - ANA and anti-dsDNA antibody assays
    - Serum anti-BG9924 antibody assay
    - Tetanus antibody titers
    - RNA Analysis
- Urinalysis (see Section 13.2 for list of variables)
- Urine pregnancy test
- Study treatment administration
- Monitoring of AEs/SAEs (see Section 15.2)
- Monitoring of concomitant therapy

14.2.3 Visit 1a and 1b (Week 0, Days 2 and 5; visit only applicable to PK sub-study)

- Blood collected for serum BG9924 PK concentration
- Monitoring of AEs/SAEs (see Section 15.2)
- Monitoring of concomitant therapy

CONFIDENTIAL
The information contained herein may not be used, disclosed, or published without the written consent of Biogen Idec Inc.

14.2.4 Visit 2 (Week 1, Day 7 ±2)

- Vital signs: temperature, heart rate, respiratory rate, and supine blood pressure (after 5 minutes' rest)
- Blood samples collected for serum BG9924 PK concentration
- Study treatment administration

14.2.5 Visit 3 (Week 2, Day 14 ±2)

- Vital signs: temperature, heart rate, respiratory rate, and supine blood pressure (after 5 minutes' rest)
- ACR core set measurements (see Section 12.1 for list of measurements)
- Blood samples collected for:
    - hsCRP and ESR (ESR to be performed at local laboratory)
    - Serum BG9924 PK concentration
    - Hematology (see Section 13.2 for list of variables)
    - Blood chemistry (see Section 13.2 for list of variables)
- Study treatment administration
- Monitoring of AEs/SAEs (see Section 15.2)
- Monitoring of concomitant therapy

14.2.6 Visit 3a (Week 3, Day 21; visit only applicable to PK sub-study)

- Blood sample collected for serum BG9924 PK concentration
- Monitoring of AEs/SAEs (see Section 15.2)
- Monitoring of concomitant therapy

14.2.7 Visit 4 (Week 4, Day 28 ±3)

- Vital signs: temperature, heart rate, respiratory rate, and supine blood pressure (after 5 minutes' rest)
- ACR core set measurements (see Section 12.1 for list of measurements)
- Blood samples collected for:
    - hsCRP and ESR (ESR to be performed at local laboratory)
    - Serum BG9924 PK concentration
    - Hematology (see Section 13.2 for list of variables)
    - Blood chemistry (see Section 13.2 for list of variables)

CONFIDENTIAL
The information contained herein may not be used, disclosed, or published without the written consent of Biogen Idec Inc.

- Study treatment administration
- Monitoring of AEs/SAEs (see Section 15.2)
- Monitoring of concomitant therapy

14.2.8 Visit 5 (Week 6, Day 42 ±3)

- Vital signs: temperature, heart rate, respiratory rate, and supine blood pressure (after 5 minutes' rest)
- ACR core set measurements (see Section 12.1 for list of measurements)
- Blood samples collected for:
    - hsCRP and ESR (ESR to be performed at local laboratory)
    - RF and anti-CCP-2 antibody
    - RA Disease Markers (serum BAFF, COMP, calgranulin, and MMP3)
    - Quantitative FACS analysis, only in subjects participating in the optional PK sub-study (see Section 12.4 for list of variables)
    - Cytokine panel (see Section 12.4 for list of variables)
    - Serum BG9924 PK concentration
    - Hematology (see Section 13.2 for list of variables)
    - Quantitative Ig (IgA, IgG, and IgM)
    - Blood chemistry (see Section 13.2 for list of variables)
    - Serum anti-BG9924 antibody assay
    - RNA Analysis
- Study treatment administration
- Monitoring of AEs/SAEs (see Section 15.2)
- Monitoring of concomitant therapy

14.2.9 Visit 6 (Week 8, Day 56 ±3)

- Vital signs: temperature, heart rate, respiratory rate, and supine blood pressure (after 5 minutes' rest)
- Blood sample collected for serum BG9924 PK concentration
- Study treatment administration
- Monitoring of AEs/SAEs (see Section 15.2)
- Monitoring of concomitant therapy

14.2.10 Visit 7 (Week 10, Day 70 ±3)

- Vital signs: temperature, heart rate, respiratory rate, and supine blood pressure (after 5 minutes' rest)
- ACR core set measurements (see Section 12.1 for list of measurements)
- Blood samples collected for:
    - hsCRP and ESR (ESR to be performed at local laboratory)
    - Serum BG9924 PK assay
    - Hematology (see Section 13.2 for list of variables)
    - Blood chemistry (see Section 13.2 for list of variables)
- Study treatment administration
- Monitoring of AEs/SAEs (see Section 15.2)
- Monitoring of concomitant therapy

14.2.11 Visit 8 (Week 12, Day 84 ±3)

- Vital signs: temperature, heart rate, respiratory rate, and supine blood pressure (after 5 minutes' rest)
- Blood sample collected for serum BG9924 PK concentration
- Study treatment administration
- Monitoring of AEs/SAEs (see Section 15.2)
- Monitoring of concomitant therapy

14.2.12 Visits 8a, 8b, and 8c (Week 12, Days 86, 89, and 91; visit only applicable to PK sub-study)

- Blood sample collected for serum BG9924 PK concentration
- Monitoring of AEs/SAEs (see Section 15.2)
- Monitoring of concomitant therapy

14.2.13 Visit 9 (Week 14, Day 98 ±3; primary endpoint evaluation, option to enter extension study, and early withdrawal visit [withdrawal prior to Week 14])

- Physical examination
- Vital signs: temperature, heart rate, respiratory rate, and supine blood pressure (after 5 minutes' rest)
- ACR core set measurements (see Section 12.1 for list of measurements)

- QoL (SF-36, FACIT-F)
- Sjogrens assessments (see Section 12.1 for assessments), if applicable
- Blood samples collected for:
    - hsCRP and ESR (ESR to be performed at local laboratory)
    - RF and anti-CCP-2 antibody
    - RA Disease Markers (serum BAFF, COMP, calgranulin, and MMP3)
    - Quantitative FACS analysis, only in subjects participating in the optional PK sub-study (see Section 12.4 for list of variables)
    - Cytokine panel (see Section 12.4 for list of variables)
    - Serum BG9924 PK assay
    - Hematology (see Section 13.2 for list of variables)
    - Quantitative Ig (IgA, IgG, and IgM)
    - Blood chemistry (see Section 13.2 for list of variables)
    - INR, PT, and aPTT
    - ANA and anti-dsDNA antibody assays
    - Serum anti-BG9924 antibody assay
    - Tetanus antibody titers
    - RNA Analysis
- Urinalysis (see Section 13.2 for list of variables)
- Urine pregnancy test
- ECG
- Monitoring of AEs/SAEs (see Section 15.2)
- Monitoring of concomitant therapy

Visit 9 (Week 14) evaluations will be completed for all subjects who complete the treatment period or who withdraw from the study prior to Visit 9/Week 14; this will be the last visit for subjects who choose to enter the safety extension study.

14.2.14 Visit 10 (Week 18, Day 126 ±3)

- Vital signs: temperature, heart rate, respiratory rate, and supine blood pressure (after 5 minutes' rest)
- ACR core set measurements (see Section 12.1 for list of measurements)
- Blood sample collected for hsCRP and ESR (ESR to be performed at local laboratory)
- Monitoring of AEs/SAEs (see Section 15.2)

- Monitoring of concomitant therapy

14.2.15 Visit 11 (Week 22, Day 154 ±3)

- Vital signs: temperature, heart rate, respiratory rate, and supine blood pressure (after 5 minutes' rest)
- ACR core set measurements (see Section 12.1 for list of measurements)
- Blood samples collected for:
    - hsCRP and ESR (ESR to be performed at local laboratory)
    - Serum BG9924 PK concentration
- Monitoring of AEs/SAEs (see Section 15.2)
- Monitoring of concomitant therapy

14.2.16 Visit 12 (Week 26, Day 182 ±3; late withdrawal visit [withdrawal after Week 14])

- Physical examination
- Vital signs: temperature, heart rate, respiratory rate, and supine blood pressure (after 5 minutes' rest)
- ACR core set measurements (see Section 12.1 for list of measurements)
- QoL (SF-36, FACIT-F)
- Sjogrens assessments (see Section 12.1 for assessments), if applicable
- Blood samples collected for:
    - hsCRP and ESR (ESR to be performed at local laboratory)
    - Serum BG9924 PK concentration
    - Hematology (see Section 13.2 for list of variables)
    - Quantitative Ig (IgA, IgG, and IgM)
    - Blood chemistry (see Section 13.2 for list of variables)
    - INR, PT, and aPTT
    - ANA and anti-dsDNA antibody assays (only from subjects with positive ANA/anti-dsDNA at Week 14)
    - Serum anti-BG9924 antibody assay
    - Tetanus antibody titers
- Urinalysis (see Section 13.2 for list of variables)
- Monitoring of AEs/SAEs (see Section 15.2)

- Monitoring of concomitant therapy

Visit 12 (Week 26) evaluations will be completed for all subjects who complete the treatment and post-treatment period, or who withdraw during the post-treatment period.

15 SAFETY DEFINITIONS, MONITORING, AND REPORTING

Throughout the course of the study, every effort must be made to remain alert to possible AEs. If an AE occurs, the first concern should be for the safety of the subject. If necessary, appropriate medical intervention should be provided.

At the signing of the ICF, each subject must be given the names and telephone numbers of study site staff for reporting AEs and medical emergencies.

15.1 Definitions

15.1.1 Adverse Event

An AE is any untoward medical occurrence in a patient or clinical investigation subject administered a pharmaceutical product and that does not necessarily have a causal relationship with this treatment. An AE can therefore be any unfavorable and unintended sign (including an abnormal laboratory finding), symptom, or disease temporally associated with the use of a medicinal (investigational) product, whether or not related to the medicinal (investigational) product.

15.1.2 Serious Adverse Event

A serious adverse event (SAE) is any untoward medical occurrence that at any dose:

- results in death
- in the view of the Investigator, places the subject at immediate risk of death (a life-threatening event); however, this does not include an event that, had it occurred in a more severe form, might have caused death
- requires inpatient hospitalization or prolongation of existing hospitalization
- results in persistent or significant disability/incapacity, or
- results in a congenital anomaly/birth defect.

An SAE may also be any other medically important event that, in the opinion of the Investigator, may jeopardize the subject or may require intervention to prevent one of the other outcomes listed in the definition above. (Examples of such medical events include allergic bronchospasm requiring intensive treatment in an emergency room or convulsions occurring at home that do not require an inpatient hospitalization.)

CONFIDENTIAL
The information contained herein may not be used, disclosed, or published without the written consent of Biogen Idec Inc.

15.1.3 Suspected Unexpected Serious Adverse Reactions

Suspected Unexpected Serious Adverse Reactions (SUSARs) are SAEs that are unexpected and judged by appropriate personnel at Biogen Idec and/or its designee to be related to the investigational product administered. SUSARs will be unblinded for reporting to the appropriate authorities by appropriate personnel at Biogen Idec DSRM or its designee. However, the Investigator and personnel involved in the analysis of the study will remain blinded. Expectedness of an adverse reaction will be determined according to the Investigators' Brochure for this study.

15.2 Monitoring and Recording Events

Any SAE experienced by the subject from the time the subject signs the ICF until the End of Study Visit, and any non-serious AE experienced by the subject after time of first dose until the End of Study Visit (see Section 7.2), is to be recorded on the CRF, regardless of the severity of the event or its relationship to study treatment. Instructions will be provided separately in the CRF Completion Guidelines.

Any AE or SAE ongoing when the subject completes the study or discontinues from the study will be followed by the Investigator until the event has resolved, stabilized, or returned to baseline status.

All events must be assessed to determine the following:

- If the event meets the criteria for an SAE as defined in Section 15.1.2.
- The relationship of the event to study treatment as defined in Section 15.3.1.
- The severity of the event as defined in Section 15.3.2.

15.2.1 Immediate Reporting of Serious Adverse Events

In order to adhere to all applicable laws and regulations for reporting an SAE, the study site must formally notify [redacted] within 24 hours of the study site staff becoming aware of the SAE. It is the Investigator's responsibility to ensure that the SAE reporting information and procedures described in Figure 15-1 are used and followed appropriately.

15.2.1.1 Death

The death must be recorded on the appropriate CRF. All causes of death must be reported as SAEs. The Investigator should make every effort to obtain and send death certificates and autopsy reports to [redacted]

If a subject's treatment assignment is unblinded for a medical emergency that ultimately leads to death, the Investigator must also document the reasons for the unblinding in the subject's CRF. *The Investigator is strongly advised not to divulge the subject's treatment*

CONFIDENTIAL
The information contained herein may not be used, disclosed, or published without the written consent of Biogen Idec Inc.

assignment to any individual not directly involved in managing the medical emergency nor to personnel involved with the analysis and conduct of the study.

Figure 15-1. Reporting Information for SAEs

Any SAE that occurs after the subject signs the ICF until Visit 12/Week 26 must be reported to [redacted] within 24 hours of the study site staff becoming aware of the event. This report must be submitted regardless of whether or not the subject has undergone any study-related procedures or received study treatment and *regardless of severity or relationship to study treatment.*

To report initial or follow-up SAE information, fax a completed SAE form to the following:

[redacted]

15.3 Safety Classifications

15.3.1 Relationship of Events to Study Treatment

The following definitions should be considered when evaluating the relationship of AEs and SAEs to the study treatment:

Relationship of Event to Study Treatment	
Unrelated	Any event that does not follow a reasonable temporal sequence from administration of study treatment *AND* that is likely to have been produced by the subject's clinical state or other modes of therapy administered to the subject.
Unlikely	Any event that does not follow a reasonable temporal sequence from administration of study treatment *OR* that is likely to have been produced by the subject's clinical state or other modes of therapy administered to the subject.
Possibly	Any reaction that follows a reasonable temporal sequence from administration of study treatment *OR* that follows a known response pattern to the suspected drug *AND* that could not be reasonably explained by the known characteristics of the subject's clinical state or other modes of therapy administered to the subject.
Related	Any reaction that follows a reasonable temporal sequence from administration of study treatment *AND* that follows a known response pattern to the suspected drug *AND* that recurs with re-challenge, *AND/OR* is improved by stopping the drug or reducing the dose.

15.3.2 Severity of Events

The following definitions should be considered when evaluating the severity of AEs and SAEs:

Severity of Event	
Mild	Symptom(s) barely noticeable to subject or does not make subject uncomfortable; does not influence performance or functioning; prescription drug not ordinarily needed for relief of symptom(s) but may be given because of personality of subject.
Moderate	Symptom(s) of a sufficient severity to make subject uncomfortable; performance of daily activity is influenced; subject is able to continue in study; treatment for symptom(s) may be needed.
Severe	Symptom(s) cause severe discomfort; symptoms cause incapacitation or significant impact on subject's daily life; severity may cause cessation of treatment with study treatment; treatment for symptom(s) may be given and/or subject hospitalized.

15.4 Prescheduled or Elective Procedures or Routinely Scheduled Treatments

A prescheduled or elective procedure or a routinely scheduled treatment shall not be considered an SAE, even if the subject is hospitalized, provided the site stipulates the following:

- The prescheduled or elective procedure or routinely scheduled treatment was scheduled (or was on a waiting list to be scheduled) prior to obtaining the subject's consent to participate in the study.
- The condition requiring the prescheduled or elective procedure or routinely scheduled treatment was present before and did not worsen or progress between the subject's consent to participate in the study and the time of the procedure or treatment.
- The prescheduled or elective procedure or routinely scheduled treatment is the sole reason for the intervention or hospital admission.

15.5 Procedures for Handling Special Situations

15.5.1 Medical Emergency

In a medical emergency requiring immediate attention, study site staff will apply appropriate medical intervention, according to current standards of care, and contact the [redacted]. In all cases, the [redacted] must be informed when a subject's treatment code has been unblinded.

15.5.2 Pregnancy

If a female subject becomes pregnant, study treatment must be discontinued *immediately*.

The Investigator must report the pregnancy by faxing the appropriate form to [redacted] within 24 hours of the study site staff becoming aware of the

pregnancy, [redacted] The Investigator or study site staff must report the outcome of the pregnancy to [redacted]

15.5.3 Unblinding

In this study, emergency decoding will be made available to the Investigator and designated personnel at Biogen Idec [redacted] through IVRS.

In a medical emergency when knowledge of the subject's treatment assignment may possibly influence the subject's clinical care, the Investigator may access the subject's treatment assignment via IVRS. However, prior to unblinding, the Investigator should attempt to contact the [redacted] to discuss the emergency whenever possible.

The Investigator must document the reasons for unblinding in the subject's source documents. *The Investigator is strongly advised not to divulge the subject's treatment assignment to any individual not directly involved in managing the medical emergency nor to personnel involved with the analysis and conduct of the study.*

15.5.3.1 Accidental Unblinding

If a subject's treatment assignment is unblinded, the subject must immediately discontinue study treatment. The subject should remain in the study and complete the study-specific follow-up tests and assessments (Week 14 to Week 26).

15.5.3.2 Unblinding for Reporting Purposes

Upon receipt of an unexpected SAE considered to be related or possibly related to the study treatment by the Investigator or the Sponsor (see Section 15.1.3), Biogen Idec DSRM or its designee will unblind the subject's treatment assignment. Regulatory authorities and central ethics committees will be notified of the event(s) when applicable, in accordance with local laws and regulations.

15.6 Investigator Responsibilities

The Investigator's responsibilities include the following:

- Monitor and record all AEs, which includes SAEs, regardless of the severity or relationship to study treatment.
- Determine the seriousness, relationship, and severity of each event.
- Determine the onset and resolution dates of each event.
- Monitor and record all pregnancies and follow-up on the outcome of the pregnancy and status of the infant.

- Complete an SAE form for each SAE and fax it to [redacted] within 24 hours of the study site staff becoming aware of the event.
- Pursue SAE follow-up information actively and persistently. Follow-up information must be reported to [redacted] within 24 hours of the study site staff becoming aware of new information.
- Ensure all AE and SAE reports are supported by documentation in the subjects' medical records.
- For sites using a local ethics committee, notify the ethics committee as per ethics committee requirements, of any SAEs. A copy of ethics committee notification must also be sent to your Clinical Monitor in a timely manner.

15.7 Biogen Idec Responsibilities

Biogen Idec's responsibilities include the following:

- Before study site activation and subject enrollment, the Biogen Idec or designee is responsible for reviewing with study site staff the definitions of AE and SAE, as well as the instructions for monitoring, recording, and reporting AEs and SAEs.
- Biogen Idec or designee is to notify all appropriate regulatory authorities, central ECs, and Investigators, of SAEs as necessary, within the required time frames and in accordance with local regulations. Biogen Idec is to complete written safety reports, with the information provided by the Investigator and study site staff, as needed, for submission to regulatory authorities and central ECs.

16 STATISTICAL STATEMENT AND ANALYTICAL PLAN

16.1 Description of Objectives and Endpoints

16.1.1 Primary Objective and Endpoints

The primary objective of the study is to evaluate the efficacy of 4 dose regimens of BG9924 when administered in combination with MTX to subjects with active RA who have had an inadequate response to conventional DMARD therapy.

The primary efficacy endpoint is the proportion of subjects with ACR50 at Week 14.

The secondary efficacy endpoints will be:

- the proportion of subjects with an ACR20 and ACR70 at Week 14,
- the change from baseline in Quality of Life (HAQ-DI, SF-36, FACIT-F) at Week 14,
- the change from baseline DAS28 at Week 14,
- the proportion of subjects with a categorical DAS28 response (EULAR response) at Week 14, and

- changes from baseline in the individual ACR core set parameters at Week 14.

16.1.2 Secondary Objectives

Secondary objectives of this study are as follows:

- To assess the safety and tolerability of these 4 dose regimens of BG9924 in this patient population.
- To assess the PK and PD profile of these 4 dose regimens of BG9924 in this patient population.

16.1.3 Exploratory Endpoints

The exploratory endpoints are:

- ACR responses over time (including ACRn)
- Change in Sjogrens assessments in subjects with Sjogrens syndrome: subject-reported dryness including VAS (ocular, vaginal, skin, and oral) and OSDI.

16.2 Criteria for the Endpoints

All statistical tests will be 2-sided with a type I error rate of 5%. Formal statistical comparisons for efficacy endpoints will be made between each BG9924 dose group versus placebo. No adjustments will be made for multiple comparisons.

Subjects with missing efficacy measurements at the visit being analyzed will be considered treatment failures for that visit. Subjects who require rescue interventions (Section 11.4) will be considered a non-responder for all analysis time points subsequent to usage of the rescue medication. Missing values for continuous endpoint values will be replaced using last observation carried forward (LOCF).

16.2.1 Demography and Baseline Disease Characteristics

Demographics and baseline data will be summarized for each treatment group by presenting basic summary statistics (mean, standard deviation, median, and range) and/or frequency distributions.

16.2.2 Pharmacokinetics

16.2.2.1 Analysis Population

The PK population will be defined as all subjects who received at least one dose of study treatment and have at least one measurable drug concentration.

16.2.2.2 Methods of Analysis

A population PK approach will be taken to determine the PK behavior of BG9924. A PK compartmental model that best describes the drug concentration vs. time data will be determined using the concentration, dose, and times of drug administration and sampling as the parameters that distinguish the model. Typical PK parameters needed to describe these data, including volume of distribution (V) and clearance (Cl), will be determined. Secondary parameters, including half-life and area under the curve (AUC), will also be reported. The maximum concentrations (C_{max}) and times to maximum concentrations (t_{max}) will be documented. Parameter estimates for the compartmental modeling will be generated for each subject using a post-hoc estimation method.

A subpopulation of subjects will have additional samples collected to provide a more definitive basis for the proper PK compartmental model. This basis of definition will be used to describe BG9924 disposition in all study subjects.

The PK will be evaluated for the possible influence of factors that may influence the disposition of BG9924. This would include, but is not limited to, subject demographics, laboratory tests indicative of hepatic or renal function, and presence of anti-drug antibodies.

16.2.3 Pharmacodynamics

16.2.3.1 Analysis Population

The pharmacodynamics population will be defined as all subjects who received at least one dose of study treatment and have an adequate number of biomarker data points.

16.2.3.2 Methods of Analysis

Pharmacodynamic measurements, including disease markers, as well as markers for drug activity, will be reviewed for the existence of patterns with time and drug exposure. If applicable, the maximum or minimum values and times to achieve those maximum and minimum values will be documented and reported.

Summary statistics and graphical presentations will be presented for each pharmacodynamic assessment, as appropriate.

16.2.4 Efficacy Data

16.2.4.1 Analysis Population

The efficacy population will be defined as all subjects who received at least one dose of study treatment. Additional analyses may be performed on subjects compliant with the dosing regimen.

16.2.4.2 Methods of Analysis

Primary endpoint: Proportion of subjects with an ACR50

The primary endpoint will be analyzed using a chi-square test, Fisher's exact test, or logistic regression with treatment group as a term in the model, as appropriate.

Secondary endpoints:

Proportion of subjects with an ACR20 and ACR70 at Week 14

The endpoints, ACR20 and ACR70 at Week 14, will be analyzed using a chi-square test, Fisher's exact test, or logistic regression with treatment group as a term in the model, as appropriate.

Change from baseline in Quality of Life (HAQ-DI, SF-36, FACIT-F) at Week 14

Change from baseline for HAQ-DI, FACIT-F scores, and SF-36 scores (mental and physical health components) will be analyzed using an analysis of covariance with treatment group and baseline as terms in the model. If the data are not normally distributed, a nonparametric analysis of covariance will be performed.

Change from baseline disease activity score (DAS28) at Week 14

The DAS28 score is a measure of the subject's disease activity and is calculated as follows:

DAS28 = 0.56 * sqrt(TJC 28) + 0.28 * sqrt(SJC 28) + 0.70 * ln (ESR) + 0.014 * GH (TJC = tender joint count; SJC = swollen joint count; ESR = erythrocyte sedimentation rate; GH = subject's global assessment)

and/or

DAS28 = 0.56 * sqrt(TJC 28) + 0.28 * sqrt(SJC 28) + 0.36 * ln (CRP+1) + 0.014 * GH + 0.96

Change from baseline disease activity score (DAS28) will be analyzed using an analysis of covariance with treatment group and baseline as terms in the model. If the data are not normally distributed, a nonparametric analysis of covariance will be performed.

Proportion of subjects with a categorical DAS28 EULAR response at Week 14

The EULAR response categories of good, moderate, and no response are defined as shown in Table 16-1 below.

CONFIDENTIAL
The information contained herein may not be used, disclosed, or published without the written consent of Biogen Idec Inc.

Table 16-1. EULAR Criteria of Response

Post-Baseline DAS28 Attained	Decrease (Improvement) in DAS28 From Baseline		
	>1.2	≤1.2 and ≥0.6	<0.6
<3.2	Good Response	Moderate Response	No Response
≥3.2 and ≤5.1	Moderate Response	Moderate Response	No Response
>5.1	Moderate Response	No Response	No Response

The proportion of subjects with a EULAR response at Week 14 will be analyzed using a chi-square test, Fisher's exact test, or logistic regression with treatment group as a term in the model, as appropriate.

Changes from baseline in the individual ACR core set parameters at Week 14

Summary statistics of change from baseline in the individual ACR core set parameters will be presented.

Exploratory endpoints:

ACR responses over time (including ACRn)

To achieve an ACRn response, a n% improvement compared to baseline is required for both swollen and tender joint counts, as well as 3 out of 5 additional parameters: subject's global assessment of disease activity, Investigator's global assessment of disease activity, subject's assessment of pain, HAQ-DI, and hsCRP or ESR.

Summary statistics of ACR responses over time (including ACRn) will be presented.

Change in Sjogrens assessments

Summary statistics of subject-reported dryness including VAS (ocular, vaginal, skin, and oral) and OSDI will be presented for subjects with Sjogrens syndrome.

Other analyses:

Rheumatoid arthritis disease markers

Summary statistics of markers of disease activity will be presented.

16.2.5 Safety Data

16.2.5.1 Analysis Population

The safety population will be defined as all subjects who received at least one dose of study treatment.

16.2.5.2 Methods of Analysis

All AEs, laboratory abnormalities, ECG, vital signs, and physical examinations will be evaluated for safety.

Clinical Adverse Events

Only treatment-emergent AEs will be presented in the summary tables. Treatment-emergent is defined as having onset date that is on or after start of study treatment, or that is worsened after the start of study treatment.

Incidence of treatment-emergent AEs will be summarized by treatment groups, overall, by severity, and by relationship to study treatment. The summary tables will include incidence estimates for overall system organ class as well as for preferred terms within each system organ class.

Laboratory Data

Laboratory data will be summarized using shift tables. Shifts from baseline to high/low status for hematology, coagulation, and blood chemistry, and shifts from baseline to high/positive status for urinalysis will be presented. In addition, the shift from baseline to the maximum post-baseline value and the shift from baseline to the minimum post-baseline status will be presented for each laboratory test by treatment group. Summary statistics may be presented for selected laboratory parameters.

Antibody Data

The proportion of subjects who developed ANA or tetanus antibodies will be summarized.

ECG Data

Changes from baseline in ECG will be summarized using shift tables. The number and percent of subjects with shifts to categorical values (abnormal, not adverse event or abnormal, adverse event) will be summarized by treatment group.

Vital Sign Data

The analysis of vital signs will focus on the incidence of clinically relevant abnormalities. The number of subjects evaluated and the number and percentage of subjects with clinically relevant post-baseline abnormalities will be presented by treatment group. The criteria for clinically relevant post-baseline abnormalities are shown in Table 16-2.

CONFIDENTIAL
The information contained herein may not be used, disclosed, or published without the written consent of Biogen Idec Inc.

Table 16-2. Criteria to Determine Clinically Relevant Abnormalities in Vital Signs

Vital Sign	Criteria for Abnormalities
Temperature	>38°C and an increase from pre-dosing of at least 1°C
Pulse	>120 beats per minute and an increase from pre-dosing of more than 20 beats per minute, or<br><50 beats per minute and a decrease from pre-dosing of more than 20 beats per minute
Systolic Blood Pressure	>180 mmHg and an increase from pre-dosing of more than 40 mmHg, or<br><90 mmHg and a decrease from pre-dosing of more than 30 mmHg
Diastolic Blood Pressure	>105 mmHg and an increase from pre-dosing of more than 30 mmHg, or<br><50 mmHg and a decrease from pre-dosing of more than 20 mmHg

Physical Examination Data

Physical examination changes will be presented using shift tables.

16.2.6 Immunogenicity Data

16.2.6.1 Analysis Population

The analysis population will be defined as all subjects who received at least one dose of study treatment and have at least one post-baseline assessment.

16.2.6.2 Methods of Analysis

The proportion of subjects who developed anti-BG9924 antibodies will be summarized.

The relationship between the antibody response and selected efficacy and safety data will be explored.

16.3 Primary Analyses

Analyses of efficacy and safety data will be performed when all subjects reach Week 14.

16.4 Sample Size Considerations

A total of 380 subjects will be randomized at a ratio of 2:2:2:2:1:1 providing 76 subjects per treatment group for every other week dosing and 38 subjects per treatment group for monthly dosing. Assuming 15% dropout, a Type I error rate of 5%, Week 14 ACR50

responses of a single BG9924 treatment group (42%) and placebo (15%), there is 90% power for a comparison of an every other week BG9924 treatment group versus placebo and 74% power for a comparison of a monthly BG9924 treatment group versus placebo by using Fisher's exact test.

17 ETHICAL REQUIREMENTS

Biogen Idec, [redacted] and the Investigator must comply with all instructions, regulations, and agreements in this protocol and applicable International Conference on Harmonisation (ICH) and Good Clinical Practice (GCP) guidelines and conduct the study according to local regulations.

17.1 Declaration of Helsinki

The Investigator must follow the recommendations contained in the Declaration of Helsinki, amended at the 52nd General Assembly in Edinburgh, Scotland, dated October 2000, with Notes of Clarification in 2002 (Washington) and 2004 (Tokyo) (Appendix G).

17.2 Ethics Committee

The Sponsor for the centralized ethics committee or the Investigator for local ethics committee must obtain ethics committee approval of the protocol, ICF, and other required study documents prior to starting the study.

If the Investigator makes any changes to the model ICF, Biogen Idec must approve the changes before the ICF is submitted to the ethics committee. A copy of the approved ICF must be provided to Biogen Idec. After approval, the ICF must not be altered without the agreement of the relevant ethics committee and Biogen Idec.

It is the responsibility of the Principal Investigator(s) to ensure that all aspects of institutional review are conducted in accordance with current governmental regulations.

Biogen Idec must receive a letter documenting ethics committee approval, which specifically identifies the protocol, protocol number and version date, and ICF version date, prior to the initiation of the study. Protocol amendments will be subject to the same requirements as the original protocol.

A progress report will be submitted to the ethics committee at required intervals and not less than annually.

At the completion or termination of the study, the investigational site must submit a close-out letter to the ethics committee and Biogen Idec.

17.3 Subject Information and Consent

Prior to any testing under this protocol, including screening tests and assessments, written informed consent with the approved ICF must be obtained from the subject in accordance

with local practice and regulations. Written informed consent in the subject's native language must be obtained from all subjects participating in a clinical study conducted by Biogen Idec.

The background of the proposed study, the procedures, and the benefits and risks of the study must be explained to the subject. The subject must be given sufficient time to consider whether to participate in the study.

A copy of the ICF, signed and dated by the subject, must be given to the subject. Confirmation of a subject's informed consent must also be documented in the subject's medical record prior to any testing under this protocol, including screening tests and assessments.

Each consent form should contain an authorization allowing the Principal Investigator(s) and Biogen Idec to use and disclose PHI (i.e., subject-identifiable health information) in compliance with local law.

The signed consent form will be retained with the study records.

17.4 Subject Data Protection

Prior to any testing under this protocol, including screening tests and assessments, candidates must also provide all authorizations required by local law (e.g., PHI authorization in North America).

The subject will not be identified by name in the CRF or in any study reports, and these reports will be used for research purposes only. Biogen Idec, its partner(s) and designee(s), and various government health agencies may inspect the records of this study. Every effort will be made to keep the subject's personal medical data confidential.

18 ADMINISTRATIVE PROCEDURES

18.1 Study Site Initiation

The Investigator must not enroll any subjects prior to completion of a study initiation visit conducted by Biogen Idec [redacted] This initiation visit will include a detailed review of the protocol and study procedures.

18.2 Quality Assurance

During and/or after completion of the study, quality assurance officers named by Biogen Idec or the regulatory authorities may wish to perform on-site audits. The Investigator will be expected to cooperate with any audit and to provide assistance and documentation (including source data) as requested.

18.3 Monitoring of the Study

The Principal Investigator(s) must permit study-related monitoring by providing direct access to source data and to the subjects' medical histories.

The Clinical Monitor(s) will visit the Investigator(s) at regular intervals during the course of the study and, as appropriate, after the study has completed.

During these visits, CRFs and supporting documentation related to the study will be reviewed and any discrepancies or omissions will be resolved.

The monitoring visits must be conducted according to the applicable ICH and GCP guidelines to ensure subject safety, protocol adherence, quality of data, study treatment accountability, compliance with regulatory requirements, and continued adequacy of the investigational site and its facilities.

18.4 Study Funding

All financial details are provided in the separate contract between the institution/Investigator and Biogen Idec.

19 FURTHER REQUIREMENTS AND GENERAL INFORMATION

19.1 External Service Organizations

19.1.1 Interactive Voice Response System

For this study, IVRS will be supplied by [redacted] Before subjects are screened or enrolled, [redacted] will provide each study site with appropriate training and a user manual.

19.1.2 Remote Data Capture

Subject information will be captured and managed by study sites on electronic CRFs via remote data capture (RDC) [redacted]

19.1.3 Data Coordinating Center

Biogen Idec and [redacted] will be responsible for all administrative aspects of this study including, but not limited to, study initiation, monitoring, management of SAEs, and data management. Prior to enrollment of the first subject at each study site, Biogen Idec [redacted] will review study responsibilities with the Investigators and other study site staff, as appropriate.

19.1.4 Central Laboratories for Laboratory Assessments

ESR will be analyzed by a local laboratory.

CONFIDENTIAL
The information contained herein may not be used, disclosed, or published without the written consent of Biogen Idec Inc.

[redacted] has been selected as the central laboratory to perform the following assessments:

- hsCRP assay
- RF
- Anti-CCP-2 antibody
- Hematology
- Blood chemistry
- Urinalysis
- Serum pregnancy
- Quantitative Ig
- INR, PT, and aPTT
- HCV, HBsAg, HBsAb, and HBcAb
- ANA
- Anti-dsDNA antibody
- Tetanus antibody titers

The following assessments will be performed by Biogen Idec or a specialized laboratory selected by Biogen Idec:

- Serum BAFF
- COMP
- Calgranulin
- MMP3
- Cytokines
- Quantitative FACS (specialized laboratory)
- Serum anti-BG9924 antibody (specialized laboratory)
- Serum PK drug concentrations (specialized laboratory)
- RNA analysis

CONFIDENTIAL
The information contained herein may not be used, disclosed, or published without the written consent of Biogen Idec Inc.

19.2 Study Committees

19.2.1 Data Monitoring Committee

An independent Data Monitoring Committee (DMC) will be formed to review interim safety data. Members of the DMC will not be allowed to participate as Investigators in this study and will not be affiliated in any way with Biogen Idec.

The DMC will review unblinded ongoing safety data on a quarterly basis to ensure the safe and proper treatment of a subject, or if the severity of an AE(s), if study treatment related, would lead to early termination of the study. The DMC will advise on the continuing safety of the study, along with the continuing validity and scientific merit. A DMC Charter will provide full guidance on the function and practices to be followed by the Committee. Investigational sites will be notified of any relevant safety findings that may jeopardize subject safety.

19.3 Changes to Final Study Protocol

All protocol amendments must be submitted to the ethics committee. Protocol modifications that affect subject safety, the scope of the investigation, or the scientific quality of the study must be approved by the ethics committee before implementation of such modifications to the conduct of the study. If required by local law, such modifications must also be approved by the appropriate regulatory agency prior to implementation.

However, Biogen Idec may, at any time, amend this protocol to eliminate an apparent immediate hazard to a subject. In this case, the appropriate regulatory authorities will be notified subsequent to the modification.

In the event of a protocol modification, the subject consent form may require similar modifications (see Sections 17.2 and 17.3).

19.4 Ethics Committee Notification of Study Completion or Termination

The Health Authority and the ethics committee in each country must be notified within 90 days of the completion of the study or within 15 days if the study is terminated early.

19.5 Retention of Study Data

The Principal Investigator(s) must maintain all Essential Documents (as listed in the ICH Guideline for GCP) until notified by Biogen Idec and in accordance with all local laws regarding retention of records.

19.6 Study Report Signatory

Biogen Idec will designate one of the participating Study Investigators as a signatory for the study report. This determination will be made by several factors, including, but not limited to, the Investigator's experience and reputation in the studied indication, the

Investigator's contribution to the study in terms of design, management, and/or subject enrollment, or by other factors determined to be relevant by Biogen Idec.

20 REFERENCES

1. Lawrence RC, Helmick CG, Arnett FC, Deyo RA, Felson DT, Giannini EH, et al. Estimates of the prevalence of arthritis and selected musculoskeletal disorders in the United States. Arthritis & Rheumatism 1998;41(5):778-99.

2. Pincus T, Brooks RH, Callahan LF. Prediction of long-term mortality in patients with rheumatoid-arthritis according to simple questionnaire and joint count measures. Ann Intern Med 1994;120(1):26-34.

3. Maradit-Kremers H, Nicola PJ, Crowson CS, Ballman KV, Gabriel SE. Cardiovascular death in rheumatoid arthritis: A population-based study. Arthritis & Rheumatism 2005;52(3):722-32.

4. Aloisi F, Pujol-Borrell R. Lymphoid neogenesis in chronic inflammatory diseases. Nat Rev Immunol 2006;6(3):205-17.

5. Browning JL, Allaire N, Ngam-Ek A, Notidis E, Hunt J, Perrin S, et al. Lymphotoxin-beta receptor signaling is required for the homeostatic control of HEV differentiation and function. Immunity 2005;23(5):539-50.

6. Gommerman JL, Browning JL. Lymphotoxin/light, lymphoid microenvironments and autoimmune disease. Nat Rev Immunol 2003;3(8):642-55.

7. Kim WJ, Kang YJ, Koh EM, Ahn KS, Cha HS, Lee WH. LIGHT is involved in the pathogenesis of rheumatoid arthritis by inducing the expression of pro-inflammatory cytokines and MMP-9 in macrophages. Immunology 2005;114(2):272-9.

8. Edwards JR, Sun SG, Locklin R, Shipman CM, Adamopoulos IE, Athanasou NA, et al. LIGHT (TNFSF14), a novel mediator of bone resorption, is elevated in rheumatoid arthritis. Arthritis Rheum 2006;54(5):1451-62.

9. Fava RA, Notidis E, Hunt J, Szanya V, Ratcliffe N, Ngam-ek A, et al. A role for the lymphotoxin/LIGHT axis in the pathogenesis of murine collagen-induced arthritis. J Immunol 2003;171(1):115-26.

10. Braun A, Takemura S, Vallejo AN, Goronzy JJ, Weyand CM. Lymphotoxin beta-mediated stimulation of synoviocytes in rheumatoid arthritis. Arthritis & Rheumatism 2004;50(7):2140-50.

CONFIDENTIAL
The information contained herein may not be used, disclosed, or published without the written consent of Biogen Idec Inc.

21 SIGNED AGREEMENT OF THE STUDY PROTOCOL

I have read the foregoing protocol, "A Phase 2b, Randomized, Double-Blind, Placebo-Controlled, Multicenter, Dose-Finding Study to Evaluate the Efficacy, Safety, Pharmacokinetics, and Pharmacodynamics of BG9924 When Given in Combination With Methotrexate to Subjects With Active Rheumatoid Arthritis Who Have Had an Inadequate Response to Conventional DMARD Therapy," Version 2, and agree to conduct the study according to the protocol and the applicable ICH guidelines and GCP regulations, and to inform all who assist me in the conduct of this study of their responsibilities and obligations.

____
Investigator's Signature Date

____
Investigator's Name (Print)

____
Study Site (Print)

CONFIDENTIAL
The information contained herein may not be used, disclosed, or published without the written consent of Biogen Idec Inc.

22 APPENDIX A: ACR REVISED CRITERIA FOR THE CLASSIFICATION OF FUNCTIONAL CAPACITY IN RA

American College of Rheumatology revised criteria for classification of functional status in rheumatoid arthritis†

Class I	Completely able to perform usual activities of daily living (self-care, vocational, and avocational)
Class II	Able to perform usual self-care and vocational activities, but limited in avocational activities
Class III	Able to perform usual self-care activities, but limited in vocational and avocational activities
Class IV	Limited in ability to perform usual self-care, vocational, and avocational activities

† Usual self-care activities include dressing, feeding, bathing, grooming, and toileting. Avocational (recreational and/or leisure) and vocational (work, school, homemaking) activities are patient-desired and age- and sex-specific.

23 APPENDIX B: ACR CORE SET MEASUREMENTS

ACR Core Set Measurements:

- Tender joint count
- Swollen joint count
- Global disease activity assessed by observer
- Global disease activity assessed by patient
- Patient assessment of pain
- Physical disability score (like health assessment questionnaire)
- Acute phase response (CRP measurement or ESR)

Joints to be Assessed for Swelling and Tenderness

The joints to be assessed for tenderness (68 joints) and swelling (66 joints) consist of the following:

- Temporomandibular joint
- Sternoclavicular joint
- Acromioclavicular joint
- Shoulders*
- Elbows*
- Wrists*
- Interphalangeal on digit 1*
- Distal interphalangeal joints on digits 2 – 5
- Proximal interphalangeal joints on digits 2 – 5*
- Metacarpophalangeal joints on digits 1 – 5*
- Hips (tenderness only)
- Knees*
- Ankles
- Metatarsals
- Interphalangeal joints on toes 1 – 5
- Metatarsophalangeal joints on toes 1 – 5

Joints assessed for swelling are the same, with the exception of the hips, which are excluded.

* The 28 joints used to calculate the DAS28.

CONFIDENTIAL
The information contained herein may not be used, disclosed, or published without the written consent of Biogen Idec Inc.

24 APPENDIX C: HEALTH ASSESSMENT QUESTIONNAIRE-DISABILITY INDEX

In this section we want to learn how your illness affects your ability to function in daily life.

Please check the response which best describes your usual abilities OVER THE PAST WEEK:

	Without ANY Difficulty	With SOME Difficulty	With MUCH Difficulty	UNABLE to Do
**DRESSING AND GROOMING**				
Are you able to:				
Dress yourself, including tying shoelaces and doing buttons?	☐	☐	☐	☐
Shampoo your hair?	☐	☐	☐	☐
**ARISING**				
Are you able to:				
Stand up from a straight chair?	☐	☐	☐	☐
Get in and out of bed?	☐	☐	☐	☐
**EATING**				
Are you able to:				
Cut your meat?	☐	☐	☐	☐
Lift a full cup or glass to your mouth?	☐	☐	☐	☐
Open a new milk carton?	☐	☐	☐	☐
**WALKING**				
Are you able to:				
Walk outdoors on flat ground?	☐	☐	☐	☐
Climb up five steps?	☐	☐	☐	☐

Please check if you use AIDS OR DEVICES for any of these activities:

- ☐ Cane
- ☐ Walker
- ☐ Crutches
- ☐ Wheelchair
- ☐ Devices used for dressing (button hook, zipper pull, long-handled shoe horn, etc.)
- ☐ Built up or special utensils
- ☐ Special or built up chair
- ☐ Other (Specify: ____________________)

Please check any categories for which you usually need HELP FROM ANOTHER PERSON:

- ☐ Dressing and Grooming
- ☐ Arising
- ☐ Eating
- ☐ Walking

CONFIDENTIAL
The information contained herein may not be used, disclosed, or published without the written consent of Biogen Idec Inc.

Please check the response which best describes your usual abilities OVER THE PAST WEEK:

	Without ANY Difficulty	With SOME Difficulty	With MUCH Difficulty	UNABLE to Do
**HYGIENE**				
Are you able to:				
Wash and dry your body?	☐	☐	☐	☐
Take a tub bath?	☐	☐	☐	☐
Get on and off the toilet?	☐	☐	☐	☐
**REACH**				
Are you able to:				
Reach and get down a 5 pound object (such as a bag of sugar) from just above your head?	☐	☐	☐	☐
Bend down to pick up clothing from the floor?	☐	☐	☐	☐
**GRIP**				
Are you able to:				
Open car doors?	☐	☐	☐	☐
Open jars which have been previously opened?	☐	☐	☐	☐
Turn faucets on and off?	☐	☐	☐	☐
**ACTIVITY**				
Are you able to:				
Run errands and shop?	☐	☐	☐	☐
Get in and out of a car?	☐	☐	☐	☐
Do chores such as vacuuming or yardwork?	☐	☐	☐	☐

Please check if you usually use AIDS OR DEVICES for any of these activities:

☐ Raised toilet seat

☐ Bathtub seat

☐ Jar opener (for jars previously opened)

☐ Bathtub bar

☐ Long-handled appliances for reach

☐ Long-handled appliances in bathroom

☐ Other (Specify: ________________________)

Please check any categories for which you usually need HELP FROM ANOTHER PERSON:

☐ Hygiene

☐ Reach

☐ Gripping and opening things

☐ Errands and chores

25 APPENDIX D: SHORT FORM 36 QUESTIONNAIRE

Your Health and Well-Being

This survey asks for your views about your health. This information will help keep track of how you feel and how well you are able to do your usual activities. *Thank you for completing this survey!*

For each of the following questions, please mark an "X" in the one box (☐) that best describes your answer.

1. In general, would you say your health is:

**Excellent**	**Very good**	**Good**	**Fair**	**Poor**
☐	☐	☐	☐	☐

2. Compared to one year ago, how would you rate your health in general now?

**Much better now than one year ago**	**Somewhat better now than one year ago**	**About the same as one year ago**	**Somewhat worse now than one year ago**	**Much worse now than one year ago**
☐	☐	☐	☐	☐

3. The following questions are about activities you might do during a typical day. Does your health now limit you in these activities? If so, how much?

	**Yes, limited a lot**	**Yes, limited a little**	**No, not limited at all**
Vigorous activities, such as running, lifting heavy objects, participating in strenuous sports	☐	☐	☐
Moderate activities, such as moving a table, pushing a vacuum cleaner, bowling, or playing golf	☐	☐	☐
Lifting or carrying groceries	☐	☐	☐
Climbing several flights of stairs	☐	☐	☐
Climbing one flight of stairs	☐	☐	☐
Bending, kneeling, or stooping	☐	☐	☐
Walking more than a mile	☐	☐	☐
Walking several hundred yards	☐	☐	☐
Walking one hundred yards	☐	☐	☐
Bathing or dressing yourself	☐	☐	☐

4. During the past 4 weeks, how much of the time have you had any of the following problems with your work or other regular daily activities as a result of your physical health?

	All of the time	Most of the time	Some of the time	A little of the time	None of the time
Cut down on the amount of time you spent on work or other activities	☐	☐	☐	☐	☐
Accomplished less than you would like	☐	☐	☐	☐	☐
Were limited in the kind of work or other activities	☐	☐	☐	☐	☐
Had difficulty performing the work or other activities (for example, it took extra effort)	☐	☐	☐	☐	☐

5. During the past 4 weeks, how much of the time have you had any of the following problems with your work or other regular daily activities as a result of any emotional problems (such as feeling depressed or anxious)?

	All of the time	Most of the time	Some of the time	A little of the time	None of the time
Cut down on the amount of time you spent on work or other activities	☐	☐	☐	☐	☐
Accomplished less than you would like	☐	☐	☐	☐	☐
Did work or other activities less carefully than usual	☐	☐	☐	☐	☐

6. During the past 4 weeks, to what extent has your physical health or emotional problems interfered with your normal social activities with family, friends, neighbors, or groups?

Not at all	Slightly	Moderately	Quite a bit	Extremely
☐	☐	☐	☐	☐

7. How much bodily pain have you had during the past 4 weeks?

None	Very mild	Mild	Moderate	Severe	Very Severe
☐	☐	☐	☐	☐	☐

8. During the past 4 weeks, how much did pain interfere with your normal work (including both work outside the home and housework)?

Not at all	A little bit	Moderately	Quite a bit	Extremely
☐	☐	☐	☐	☐

9. These questions are about how you feel and how things have been with you during the past 4 weeks. For each question, please give the one answer that comes closest to the way you have been feeling. How much of the time during the past 4 weeks…

	All of the time	Most of the time	Some of the time	A little of the time	None of the time
Did you feel full of life?	☐	☐	☐	☐	☐
Have you been very nervous?	☐	☐	☐	☐	☐
Have you felt so down in the dumps that nothing could cheer you up?	☐	☐	☐	☐	☐
Have you felt calm and peaceful?	☐	☐	☐	☐	☐
Did you have a lot of energy?	☐	☐	☐	☐	☐
Have you felt downhearted and depressed?	☐	☐	☐	☐	☐
Did you feel worn out?	☐	☐	☐	☐	☐
Have you been happy?	☐	☐	☐	☐	☐
Did you feel tired?	☐	☐	☐	☐	☐

10. During the past 4 weeks, how much of the time has your physical health or emotional problems interfered with your social activities (like visiting friends, relatives, etc.)?

All of the time	Most of the time	Some of the time	A little of the time	None of the time
☐	☐	☐	☐	☐

11. How TRUE or FALSE is each of the following statements for you?

	**Definitely true**	**Mostly true**	**Don't know**	**Mostly false**	**Definitely false**
I seem to get sick a little easier than other people	☐	☐	☐	☐	☐
I am as healthy as anybody I know	☐	☐	☐	☐	☐
I expect my health to get worse	☐	☐	☐	☐	☐
My health is excellent	☐	☐	☐	☐	☐

THANK YOU FOR COMPLETING THESE QUESTIONS!

SF-36v2™ Health Survey ©1996, 2000 by QualityMetric Incorporated and Medical Outcomes Trust. All Rights Reserved.
SF-36® is a registered trademark of Medical Outcomes Trusts.
(SF-36v2 Standard, US Version 2.0)

26 APPENDIX E: FUNCTIONAL ASSESSMENT OF CHRONIC ILLNESS THERAPY-FATIGUE QUESTIONNAIRE (VERSION 4)

Below is a list of statements that other people with your illness have said are important. **By circling one (1) number per line, please indicate how true each statement has been for you <u>during the past 7 days.</u>**

<u>PHYSICAL WELL-BEING</u>

		Not at all	A little	Some what	Quite a bit	Very much
1.	I have a lack of energy.	0	1	2	3	4
2.	I have nausea.	0	1	2	3	4
3.	Because of my physical condition, I have trouble meeting the needs of my family.	0	1	2	3	4
4.	I have pain.	0	1	2	3	4
5.	I am bothered by side effects of treatment.	0	1	2	3	4
6.	I feel ill.	0	1	2	3	4
7.	I am forced to spend time in bed.	0	1	2	3	4

<u>SOCIAL/FAMILY WELL-BEING</u>

		Not at all	A little	Some what	Quite a bit	Very much
1.	I feel close to my friends.	0	1	2	3	4
2.	I get emotional support from my family.	0	1	2	3	4
3.	I get support from my friends.	0	1	2	3	4
4.	My family has accepted my illness.	0	1	2	3	4
5.	I am satisfied with family communication about my illness.	0	1	2	3	4
6.	I feel close to my partner (or the person who is my main support).	0	1	2	3	4

CONFIDENTIAL
The information contained herein may not be used, disclosed, or published without the written consent of Biogen Idec Inc.

Regardless of your current level of sexual activity, Please answer the following question. If you prefer not to answer it, please check this box ☐ and go on to the next section.

7.	I am satisfied with my sex life.	0	1	2	3	4

EMOTIONAL WELL-BEING

		Not at all	A little	Some what	Quite a bit	Very much
1.	I feel sad.	0	1	2	3	4
2.	I am satisfied with how I am coping with my illness.	0	1	2	3	4
3.	I am losing hope in the fight against my illness.	0	1	2	3	4
4.	I feel nervous.	0	1	2	3	4
5.	I worry about dying.	0	1	2	3	4
6.	I worry that my condition will get worse.	0	1	2	3	4

FUNCTIONAL WELL-BEING

		Not at all	A little	Some what	Quite a bit	Very much
1.	I am able to work (include work at home).	0	1	2	3	4
2.	My work (include work at home) is fulfilling.	0	1	2	3	4
3.	I am able to enjoy life.	0	1	2	3	4
4.	I have accepted my illness.	0	1	2	3	4
5.	I am sleeping well.	0	1	2	3	4
6.	I am enjoying the things I usually do for fun.	0	1	2	3	4
7.	I am content with the quality of my life right now.	0	1	2	3	4

CONFIDENTIAL
The information contained herein may not be used, disclosed, or published without the written consent of Biogen Idec Inc.

By circling one (1) number per line, please indicate how true each statement has been for you during the past 7 days.

ADDITIONAL CONCERNS

	Not at all	A little	Some what	Quite a bit	Very much
1. I feel fatigued.	0	1	2	3	4
2. I feel weak all over.	0	1	2	3	4
3. I feel listless (“washed out”).	0	1	2	3	4
4. I feel tired.	0	1	2	3	4
5. I have trouble starting things because I am tired.	0	1	2	3	4
6. I have trouble finishing things because I am tired.	0	1	2	3	4
7. I have energy.	0	1	2	3	4
8. I am able to do my usual activities.	0	1	2	3	4
9. I need to sleep during the day.	0	1	2	3	4
10. I am too tired to eat.	0	1	2	3	4
11. I need help doing my usual activities.	0	1	2	3	4
12. I am frustrated by being too tired to do the things I want to do.	0	1	2	3	4
13. I have to limit my social activity because I am tired.	0	1	2	3	4

US English
Copyright 1987, 1997 1/22/2007

27 APPENDIX F: OCULAR SURFACE DISEASE INDEX© (OSDI©)

Ask your patient the following 12 questions, and circle the number in the box that best represents each answer.

Have you experienced any of the following DURING THE LAST WEEK:

	All of the time	Most of the time	Half of the time	Some of the time	None of the time
1. Eyes that are sensitive to light?	4	3	2	1	0
2. Eyes that feel gritty?	4	3	2	1	0
3. Painful or sore eyes?	4	3	2	1	0
4. Blurred vision?	4	3	2	1	0
5. Poor vision?	4	3	2	1	0

Have problems with your eyes limited you in performing any of the following DURING THE LAST WEEK:

	All of the time	Most of the time	Half of the time	Some of the time	None of the time	
6. Reading?	4	3	2	1	0	N/A
7. Driving at night?	4	3	2	1	0	N/A
8. Working with a computer or bank machine (ATM)?	4	3	2	1	0	N/A
9. Watching TV?	4	3	2	1	0	N/A

Have your eyes felt uncomfortable in any of the following situations DURING THE LAST WEEK:

	All of the time	Most of the time	Half of the time	Some of the time	None of the time	
10. Windy conditions?	4	3	2	1	0	N/A
11. Places or areas with low humidity (very dry)?	4	3	2	1	0	N/A
12. Areas that are air conditioned?	4	3	2	1	0	N/A

Reference: 1. Schiffman RM, Christianson MD, Jacobsen G, Hirsch JD, Reis BL. Reliability and validity of the Ocular Surface Disease Index. Arch Ophthalmol. 2000; 118:615-621.

28 APPENDIX G: WORLD MEDICAL ASSOCIATION DECLARATION OF HELSINKI

Policy

WORLD MEDICAL ASSOCIATION DECLARATION OF HELSINKI
Ethical Principles for Medical Research Involving Human Subjects

Adopted by the 18th WMA General Assembly, Helsinki, Finland, June 1964, and amended by the
29th WMA General Assembly, Tokyo, Japan, October 1975
35th WMA General Assembly, Venice, Italy, October 1983
41st WMA General Assembly, Hong Kong, September 1989
48th WMA General Assembly, Somerset West, Republic of South Africa, October 1996
and the 52nd WMA General Assembly, Edinburgh, Scotland, October 2000
Note of Clarification on Paragraph 29 added by the WMA General Assembly, Washington 2002
Note of Clarification on Paragraph 30 added by the WMA General Assembly, Tokyo 2004

A. INTRODUCTION

1. The World Medical Association has developed the Declaration of Helsinki as a statement of ethical principles to provide guidance to physicians and other participants in medical research involving human subjects. Medical research involving human subjects includes research on identifiable human material or identifiable data.

2. It is the duty of the physician to promote and safeguard the health of the people. The physician's knowledge and conscience are dedicated to the fulfillment of this duty.

3. The Declaration of Geneva of the World Medical Association binds the physician with the words, "The health of my patient will be my first consideration," and the International Code of Medical Ethics declares that, "A physician shall act only in the patient's interest when providing medical care which might have the effect of weakening the physical and mental condition of the patient."

4. Medical progress is based on research which ultimately must rest in part on experimentation involving human subjects.

5. In medical research on human subjects, considerations related to the well-being of the human subject should take precedence over the interests of science and society.

6. The primary purpose of medical research involving human subjects is to improve prophylactic, diagnostic and therapeutic procedures and the understanding of the aetiology and pathogenesis of disease. Even the best proven prophylactic, diagnostic, and therapeutic methods must continuously be challenged through research for their effectiveness, efficiency, accessibility and quality.

7. In current medical practice and in medical research, most prophylactic, diagnostic and therapeutic procedures involve risks and burdens.

8. Medical research is subject to ethical standards that promote respect for all human beings and protect their health and rights. Some research populations are vulnerable and need special protection. The particular needs of the economically and medically disadvantaged must be recognized. Special attention is also required for those who cannot give or refuse consent for themselves, for those who may be subject to giving consent under duress, for those who will not benefit personally from the research and for those for whom the research is combined with care.

9. Research Investigators should be aware of the ethical, legal and regulatory requirements for research on human subjects in their own countries as well as applicable international requirements. No national ethical, legal or regulatory requirement should be allowed to reduce or eliminate any of the protections for human subjects set forth in this Declaration.

B. BASIC PRINCIPLES FOR ALL MEDICAL RESEARCH

10. It is the duty of the physician in medical research to protect the life, health, privacy, and dignity of the human subject.

11. Medical research involving human subjects must conform to generally accepted scientific principles, be based on a thorough knowledge of the scientific literature, other relevant sources of information, and on adequate laboratory and, where appropriate, animal experimentation.

12. Appropriate caution must be exercised in the conduct of research which may affect the environment, and the welfare of animals used for research must be respected.

13. The design and performance of each experimental procedure involving human subjects should be clearly formulated in an experimental protocol. This protocol should be submitted for consideration, comment, guidance, and where appropriate, approval to a specially appointed ethical review committee, which must be independent of the investigator, the sponsor or any other kind of undue influence. This independent committee should be in conformity with the laws and regulations of the country in which the research experiment is performed. The committee has the right to monitor ongoing trials. The researcher has the obligation to provide monitoring information to the committee, especially any serious adverse events. The researcher should also submit to the committee, for review, information regarding funding, sponsors, institutional affiliations, other potential conflicts of interest and incentives for subjects.

14. The research protocol should always contain a statement of the ethical considerations involved and should indicate that there is compliance with the principles enunciated in this Declaration.

15. Medical research involving human subjects should be conducted only by scientifically qualified persons and under the supervision of a clinically competent medical person. The responsibility for the human subject must always rest with a medically qualified person and never rest on the subject of the research, even though the subject has given consent.

16. Every medical research project involving human subjects should be preceded by careful assessment of predictable risks and burdens in comparison with foreseeable benefits to the subject or to others. This does not preclude the participation of healthy volunteers in medical research. The design of all studies should be publicly available.

17. Physicians should abstain from engaging in research projects involving human subjects unless they are confident that the risks involved have been adequately assessed and can be satisfactorily managed. Physicians should cease any investigation if the risks are found to outweigh the potential benefits or if there is conclusive proof of positive and beneficial results.

18. Medical research involving human subjects should only be conducted if the importance of the objective outweighs the inherent risks and burdens to the subject. This is especially important when the human subjects are healthy volunteers.

19. Medical research is only justified if there is a reasonable likelihood that the populations in which the research is carried out stand to benefit from the results of the research.

20. The subjects must be volunteers and informed participants in the research project.

21. The right of research subjects to safeguard their integrity must always be respected. Every precaution should be taken to respect the privacy of the subject, the confidentiality of the patient's information and to minimize the impact of the study on the subject's physical and mental integrity and on the personality of the subject.

22. In any research on human beings, each potential subject must be adequately informed of the aims, methods, sources of funding, any possible conflicts of interest, institutional affiliations of the researcher, the anticipated benefits and potential risks of the study and the discomfort it may entail. The subject should be informed of the right to abstain from participation in the study or to withdraw consent to participate at any time without reprisal. After ensuring that the subject has understood the information, the physician should then obtain the subject's freely-given informed consent, preferably in writing. If the consent cannot be obtained in writing, the non-written consent must be formally documented and witnessed.

23. When obtaining informed consent for the research project the physician should be particularly cautious if the subject is in a dependent relationship with the physician or may consent under duress. In that case the informed consent should be obtained by a well-informed physician who is not engaged in the investigation and who is completely independent of this relationship.

24. For a research subject who is legally incompetent, physically or mentally incapable of giving consent or is a legally incompetent minor, the investigator must obtain informed consent from the legally authorized representative in

accordance with applicable law. These groups should not be included in research unless the research is necessary to promote the health of the population represented and this research cannot instead be performed on legally competent persons.

25. When a subject deemed legally incompetent, such as a minor child, is able to give assent to decisions about participation in research, the investigator must obtain that assent in addition to the consent of the legally authorized representative.

26. Research on individuals from whom it is not possible to obtain consent, including proxy or advance consent, should be done only if the physical/mental condition that prevents obtaining informed consent is a necessary characteristic of the research population. The specific reasons for involving research subjects with a condition that renders them unable to give informed consent should be stated in the experimental protocol for consideration and approval of the review committee. The protocol should state that consent to remain in the research should be obtained as soon as possible from the individual or a legally authorized surrogate.

27. Both authors and publishers have ethical obligations. In publication of the results of research, the investigators are obliged to preserve the accuracy of the results. Negative as well as positive results should be published or otherwise publicly available. Sources of funding, institutional affiliations and any possible conflicts of interest should be declared in the publication. Reports of experimentation not in accordance with the principles laid down in this Declaration should not be accepted for publication.

C. ADDITIONAL PRINCIPLES FOR MEDICAL RESEARCH COMBINED WITH MEDICAL CARE

28. The physician may combine medical research with medical care, only to the extent that the research is justified by its potential prophylactic, diagnostic or therapeutic value. When medical research is combined with medical care, additional standards apply to protect the patients who are research subjects.

29. The benefits, risks, burdens and effectiveness of a new method should be tested against those of the best current prophylactic, diagnostic, and therapeutic methods. This does not exclude the use of placebo, or no treatment, in studies where no proven prophylactic, diagnostic or therapeutic method exists.[1]

30. At the conclusion of the study, every patient entered into the study should be assured of access to the best proven prophylactic, diagnostic and therapeutic methods identified by the study.[2]

31. The physician should fully inform the patient which aspects of the care are related to the research. The refusal of a patient to participate in a study must never interfere with the patient-physician relationship.

32. In the treatment of a patient, where proven prophylactic, diagnostic and therapeutic methods do not exist or have been ineffective, the physician, with informed consent from the patient, must be free to use unproven or new prophylactic, diagnostic and therapeutic measures, if in the physician's judgement it offers hope of saving life, re-establishing health or alleviating suffering. Where possible, these measures should be made the object of research, designed to evaluate their safety and efficacy. In all cases, new information should be recorded and, where appropriate, published. The other relevant guidelines of this Declaration should be followed.

---

[1] **Note of clarification on paragraph 29 of the WMA Declaration of Helsinki**

The WMA hereby reaffirms its position that extreme care must be taken in making use of a placebo-controlled trial and that in general this methodology should only be used in the absence of existing proven therapy. However, a placebo-controlled trial may be ethically acceptable, even if proven therapy is available, under the following circumstances:

- Where for compelling and scientifically sound methodological reasons its use is necessary to determine the efficacy or safety of a prophylactic, diagnostic or therapeutic method; or

- Where a prophylactic, diagnostic or therapeutic method is being investigated for a minor condition and the patients who receive placebo will not be subject to any additional risk of serious or irreversible harm.

All other provisions of the Declaration of Helsinki must be adhered to, especially the need for appropriate ethical and scientific review.

[2] **Note of clarification on paragraph 30 of the WMA Declaration of Helsinki**

The WMA hereby reaffirms its position that it is necessary during the study planning process to identify post-trial access by study participants to prophylactic, diagnostic and therapeutic procedures identified as beneficial in the study or access to other appropriate care. Post-trial access arrangements or other care must be described in the study protocol so the ethical review committee may consider such arrangements during its review.

9.10.2004
